# Low temperature and mTOR inhibition favor stem cell maintenance in human keratinocyte cultures

**DOI:** 10.15252/embr.202255439

**Published:** 2023-05-04

**Authors:** Daisuke Nanba, Jun‐Ichi Sakabe, Johannes Mosig, Michel Brouard, Fujio Toki, Mariko Shimokawa, Mako Kamiya, Thomas Braschler, Fahd Azzabi, Stéphanie Droz‐Georget Lathion, Kai Johnsson, Keya Roy, Christoph D Schmid, Jean‐Baptiste Bureau, Ariane Rochat, Yann Barrandon

**Affiliations:** ^1^ Laboratory of Stem Cell Dynamics, School of Life Sciences École Polytechnique Fédérale de Lausanne Lausanne Switzerland; ^2^ Department of Experimental Surgery Lausanne University Hospital Lausanne Switzerland; ^3^ Division of Aging and Regeneration, The Institute of Medical Science The University of Tokyo Tokyo Japan; ^4^ Duke‐NUS Medical School Singapore City Singapore; ^5^ Department of Plastic, Reconstructive and Aesthetic Surgery Singapore General Hospital and A*STAR Skin Research Labs Singapore City Singapore; ^6^ Department of Pathology and Immunology University of Geneva Geneva Switzerland; ^7^ Institute of Chemical Sciences and Engineering École Polytechnique Fédérale de Lausanne Lausanne Switzerland; ^8^ Friedrich Miescher Institute for Biomedical Research Basel Switzerland; ^9^ Present address: School of Life Science and Technology Tokyo Institute of Technology Yokohama Japan; ^10^ Present address: Max Planck Institute for Medical Research Heidelberg Germany

**Keywords:** keratinocyte stem cells, microenvironment, mTOR, temperature, TRP channels, Methods & Resources, Signal Transduction, Stem Cells & Regenerative Medicine

## Abstract

Adult autologous human epidermal stem cells can be extensively expanded *ex vivo* for cell and gene therapy. Identifying the mechanisms involved in stem cell maintenance and defining culture conditions to maintain stemness is critical, because an inadequate environment can result in the rapid conversion of stem cells into progenitors/transient amplifying cells (clonal conversion), with deleterious consequences on the quality of the transplants and their ability to engraft. Here, we demonstrate that cultured human epidermal stem cells respond to a small drop in temperature through thermoTRP channels via mTOR signaling. Exposure of cells to rapamycin or a small drop in temperature induces the nuclear translocation of mTOR with an impact on gene expression. We also demonstrate by single‐cell analysis that long‐term inhibition of mTORC1 reduces clonal conversion and favors the maintenance of stemness. Taken together, our results demonstrate that human keratinocyte stem cells can adapt to environmental changes (e.g., small variations in temperature) through mTOR signaling and constant inhibition of mTORC1 favors stem cell maintenance, a finding of high importance for regenerative medicine applications.

## Graphical abstract

Cultured human epidermal keratinocyte stem cells respond to a small drop in temperature through thermoTRP channels connected to mTORC1 signaling. mTORC1 inhibition favors the maintenance of a stem cell phenotype in human keratinocyte cultures.

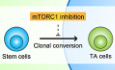

## Introduction

The epidermis, the outer layer of the skin, is a stratified epithelium mainly composed of keratinocytes that are responsible for the structure, cohesion, and barrier function of the epithelium. The epidermis is constantly renewed, and its basal layer contains stem cells and progenitors/transient amplifying cells whose controlled multiplication balances the loss of anucleated terminally differentiated cells (squames) that are continuously sloughed off (Barrandon & Green, 1987; Gambardella & Barrandon, 2003; Clayton *et al*, 2007; Barrandon *et al*, 2012; Mascre *et al*, 2012; Rompolas *et al*, 2016). Owing to their location at the interface with the external environment, stem cells of the epidermis are particularly exposed to environmental variations such as fluctuating temperature and pH and the availability of nutrients and oxygen. For instance, human epidermal stem cells located on the surface of the skin reside in a cooler environment than hair follicle stem cells located deeper in the vascularized dermis (Kobayashi *et al*, 1993; Rochat *et al*, 1994; Oshima *et al*, 2001). However, little is known about how temperature affects stem cell metabolism and behavior, even though the temperature is used in therapy, for instance, to treat alopecia areata (Lei *et al*, 1991; Atanaskova Mesinkovska, 2018) or to prevent hair loss following chemotherapy (Nangia *et al*, 2017; Rugo *et al*, 2017).

There is accumulating evidence that keratinocytes sense their environment through the activation of ion channels with sensory properties (Lumpkin & Caterina, 2007; Venkatachalam & Montell, 2007; Blaydon & Kelsell, 2014), most of which belong to the transient receptor potential (TRP) ion channel superfamily. The mammalian TRP channel superfamily comprises 28 members and is subdivided into six subfamilies, including ankyrin (TRPA), canonical (TRPC), melastatin (TRPM), mucolipin (TRPML), polycystin (TRPP), and vanilloid (TRPV; Pedersen *et al*, 2005). Temperature‐sensitive TRP channels, referred to as thermoTRP channels, are the central components of thermoception. When thermoTRP channels are activated by temperature, they open to allow the permeation of cations into cells. Mouse keratinocytes have been found to functionally express TRPV3 and TRPV4 (Güler *et al*, 2002; Peier *et al*, 2002). TRPV3 null mice have curly whiskers and impaired barrier function due to dysregulation of TGF‐α/EGFR signaling (Cheng *et al*, 2010); TRPV3 is also involved in the formation of corneocytes from keratinocytes of the upper stratum granulosum (Matsui *et al*, 2021). Conditional ablation of TRPV3 in the skin impairs thermosensation (Moqrich *et al*, 2005). Moreover, a gain‐of‐function mutation in TRPV3 (Gly573Ser substitution) is associated with hairlessness and atopic dermatitis (Asakawa *et al*, 2006; Yoshioka *et al*, 2009). Mutations in TRPV3 have also been linked to Olmsted syndrome and erythromelalgia (Lin *et al*, 2012; Duchatelet *et al*, 2014). TRPV4 null mice also exhibit an abnormal epidermal barrier (Sokabe *et al*, 2010). In addition, epidermal TRPM8 is involved in keratinocyte proliferation and epidermal homeostasis (Denda *et al*, 2010; Bidaux *et al*, 2015). Taken together, these findings strongly suggest that thermoTRP channels are implicated in hair and epidermal renewal and that temperature is an important component of keratinocyte stem cell microenvironment.

Mechanistic (originally “mammalian”) target of rapamycin (mTOR) signaling axis is critical for stem cells because it allows cells to adapt to environmental changes and regulates major cellular functions such as growth, proliferation, and survival (Shimobayashi & Hall, 2014; Liu & Sabatini, 2020). mTOR is a highly conserved serine/threonine kinase that interacts with several companion proteins to form two independent complexes: mTORC1 and mTORC2. Upon activation, mTORC1 increases the production of proteins, lipids, nucleotides, and ATP, whereas mTORC2 controls the actin cytoskeleton network and cellular metabolism (Liu & Sabatini, 2020). Rapamycin specifically binds to the rapamycin‐binding protein FKBP12 to form a complex that is associated with mTORC1 and not mTORC2 (Wullschleger *et al*, 2006) and inhibits mTORC1 allosterically by binding to the FBR domain in mTOR (Benjamin *et al*, 2011). However, prolonged exposure to rapamycin may reduce mTORC2 activity (Phung *et al*, 2006; Sarbassov *et al*, 2006).

The human epidermis contains a population of clonogenic keratinocyte stem cells (Rheinwald & Green, 1975; Barrandon & Green, 1985) that can be extensively cultured and transplanted to regenerate the epidermis, a property used in regenerative medicine to treat extensive third‐degree burn wounds (O'Connor *et al*, 1981; Gallico *et al*, 1984; Pellegrini *et al*, 1999; Ronfard *et al*, 2000; Green, 2008) and for *ex vivo* gene therapy of disabling hereditary skin diseases (Mavilio *et al*, 2006; Droz‐Georget Lathion *et al*, 2015; Hirsch *et al*, 2017). Human clonogenic keratinocytes have different growth capacities in culture and can be classified by clonal analysis as holoclones, meroclones, or paraclones (Barrandon & Green, 1987). It is widely accepted that holoclones are stem cells, whereas meroclones and paraclones are progenitor/transient amplifying cells (Blanpain *et al*, 2007; Barrandon *et al*, 2012). Holoclones have a growth capacity of at least 180 divisions in culture and can generate progeny large enough to reconstruct the entire epidermis of an adult human (1.73 m^2^; Barrandon & Green, 1987; Droz‐Georget Lathion *et al*, 2015; Mathor *et al*, 1996; Rochat *et al*, 1994). However, holoclones can be rapidly lost through clonal conversion, a phenomenon that affects stem cell maintenance and is independent of replicative senescence (Barrandon *et al*, 2012; Nanba *et al*, 2013). Clonal conversion results from the conversion of holoclones (stem cells) to meroclones and paraclones in response to a deleterious environment such as inadequate culture conditions. Consequently, the loss of holoclones substantially affects the quality and efficacy of the transplants produced for *ex vivo* autologous cell and gene therapy (Nanba, 2019). Hence, it is paramount to minimize clonal conversion when keratinocyte stem cells are expanded for cell therapy.

Here, we demonstrated that cultured keratinocyte stem cells sense and respond to minor drops in temperature through thermoTRP channels and inhibition of mTORC1 signaling, which in turn favors stem cell maintenance.

## Results

### Keratinocyte stem cells are exposed to temperature variations

**Figure EV1:**
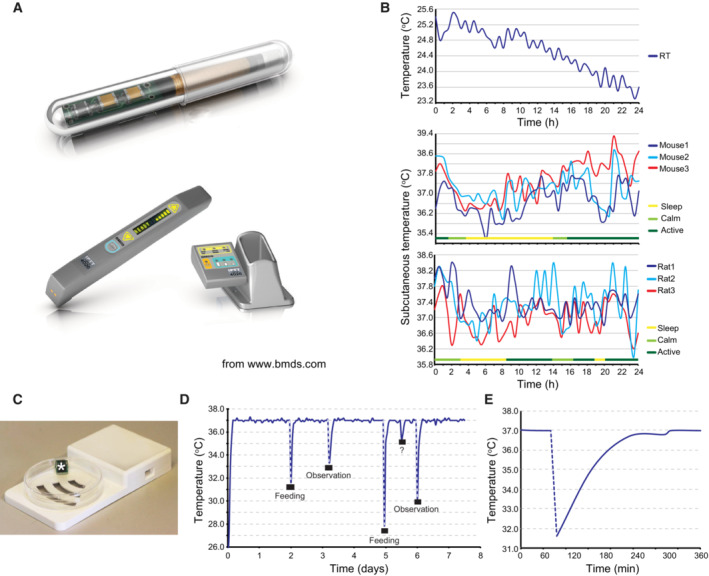
Keratinocyte stem cells are constantly exposed to temperature variations *in vivo* and *in vitro* Implantable IPTT‐300 transponder and its DAS‐7006/7 s reader system from BioMedic Data Systems, Seaford DE, USA (www.bmds.com). Actual size of the implantable transponder: length 14 mm and diameter 2 mm.Subcutaneous temperature of mice and rats constantly fluctuates. Three mice and three rats were each implanted subcutaneously with an IPTT‐300 transponder (BioMedic Data Systems); the temperature and the behavior (active, calm, sleep) of each animal were then monitored over a 24 h. Room temperature (RT) was simultaneously monitored with a nonimplanted transponder.Picture of an EPFL‐designed submersible RFID temperature sensor and its reading base station (Laboratory of Microengineering for Manufacturing). The culture vessel was a 100 mm size Petri dish.Cultured human keratinocytes were subjected to sharp temperature fluctuations when removal from the culture incubator (Nuaire 8700E) for medium change (feeding) or microscopic observation. Temperature of the culture medium was recorded over a week using immersed temperature sensors. Note that an unknown event (opening of the incubator door?) occurred between the fifth and sixth day of culture.Zoom of the temperature curve at the first medium change shown in B demonstrates that recovery to the set temperature (37°C) takes more than 2 h.

We have previously demonstrated that the subcutaneous temperature of a pig constantly fluctuates and is always below core body temperature (Merli *et al*, 2012). To confirm these observations, we monitored the subcutaneous temperature of individual mice and rats every half an hour for 24 h using small (14 mm length and 2 mm diameter), sensitive (± 0.1°C), and individually identifiable radio‐frequency identification (RFID) transponders implanted under the skin in the neck region (Fig EV1A). The temperature of the room in which the animals were housed was measured in parallel using a nonimplanted transponder. The activities (sleep, calm, and active) of the animals were also documented (Fig EV1B). Measurements were initiated 2 weeks after the implantation of the transponders to exclude temperature variations related to wound healing. These experiments revealed that the subcutaneous temperature of mice and rats continuously oscillated with variations ranging from 2°C in rats to 3°C in mice (Fig EV1B). These experiments demonstrate that temperature is an important component of the keratinocyte stem cells microenvironment. For ethical reasons, it is impossible to thoroughly investigate the impact of minor variations in temperature on human keratinocyte stem cells *in vivo*; nevertheless, cultured human keratinocytes are used in regenerative medicine. Therefore, we focused our investigation on cultured stem cells. RFID temperature sensor cells were glued to the side of 100 mm Petri dishes containing sufficient culture medium so that the sensors were immersed (Fig EV1C). Cells were then cultured as described previously (Rochat *et al*, 1994) in culture incubators with temperature set at 37°C. The temperature of the culture medium was then continuously recorded for 7 days. As expected, the temperature of the medium remained constant when the cultures were maintained in the incubator. However, each manipulation of the culture, such as cell feeding or microscopic observation, resulted in a dramatic temperature drop (1‐5°C; Fig EV1D). When cultures were returned to the incubators, the temperature of the medium slowly increased back to 37°C, and this process could take up to 3 h (Fig EV1E). These experiments demonstrated that cultured cells were regularly exposed to significant temperature variations.

**Figure 1:**
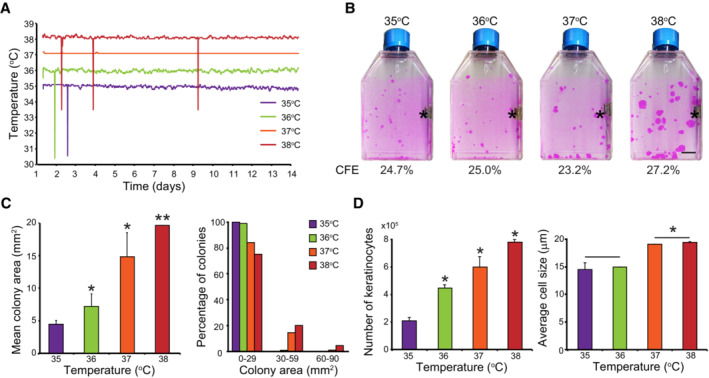
Temperature impacts long‐term growth of keratinocyte stem cells A, BOne hundred human keratinocytes (YF29, passage V) were cultured in duplicate for 12 days in individual Thermo Scientific Heracell 150 incubators with the temperature set at 35, 36, 37, or 38°C. Temperature of each incubator was monitored with independent thermo probes (see Fig EV1). (A) In parallel, the temperature of the culture medium was recorded every 10 s using immerged RFID temperature sensors (white stars in panel B; see also Fig EV1C). Sharp spikes resulted from wireless communication artifacts. (B) Cells were fixed and stained with 1% Rhodamine B. Temperature did not affect colony‐forming efficiency (CFE) but impacted colony size; colonies were smaller when temperatures were below 37°C and larger at 38°C. Asterisks indicate thermo probes for monitoring the temperature. Scale bar, 10 mm.C, DHuman keratinocytes (YF29) were cultured in mass for 7 days at different temperatures before the cultures were dissociated. (C) Colony size was quantified using an image processing software (ImageJ). (D) The number and the size of the cells were determined using a Tali™ cytometer. Temperature impacted both cell number and size. Data information: In (C and D), data are presented as the mean ± SD (*n* = 4 biological replicates). *P < 0.05, **P < 0.01 (two‐tailed Student's *t*‐test). The data shown were confirmed in two technical replicates.

### Temperature impacts human keratinocyte stem cell behavior

To evaluate the influence of temperature on cultured human keratinocyte stem cells, we determined colony‐forming efficiency (CFE) at four different temperatures: 35, 36, 37, and 38°C. Keratinocyte stem cells (strain YF29) were plated at clonal density in conditions comparable to those of human cell therapy (Ronfard *et al*, 2000) and cultured for 14 days. The temperature was measured every 10 min during the entire experiment using specific RFID thermosensors immersed in the culture medium (Fig 1A). Cultures were fixed and stained, and the number and area of colonies were calculated using image processing software (ImageJ). The temperature did not affect colony‐forming efficiency; however, the colony size was temperature‐dependent, as the colony mean area was significantly larger with each temperature increment (Fig 1B). Interestingly, the proportion of small colonies (smaller than 30 mm^2^) was fairly similar under all conditions, suggesting that only cells with high proliferative potential were influenced by temperature (Fig 1C). We then examined whether the difference in colony size was related to differences in proliferation or variations in cell size. YF29 keratinocytes were cultured for 7 days at different temperatures (35, 36, 37, and 38°C) dissociated and analyzed with a Tali™ hemocytometer. These experiments demonstrated that the number of cells increased with increasing temperature and that the cell size was significantly smaller at the lowest temperature (Fig 1D). Cell size is closely associated with the differentiation and proliferative potential of human keratinocytes (Watt & Green, 1981; Barrandon & Green, 1985), which strongly suggests that temperature impacts human keratinocyte stem cell maintenance.

### Brief temperature fluctuation activates calcium‐permissive thermoTRP channels

**Figure 2:**
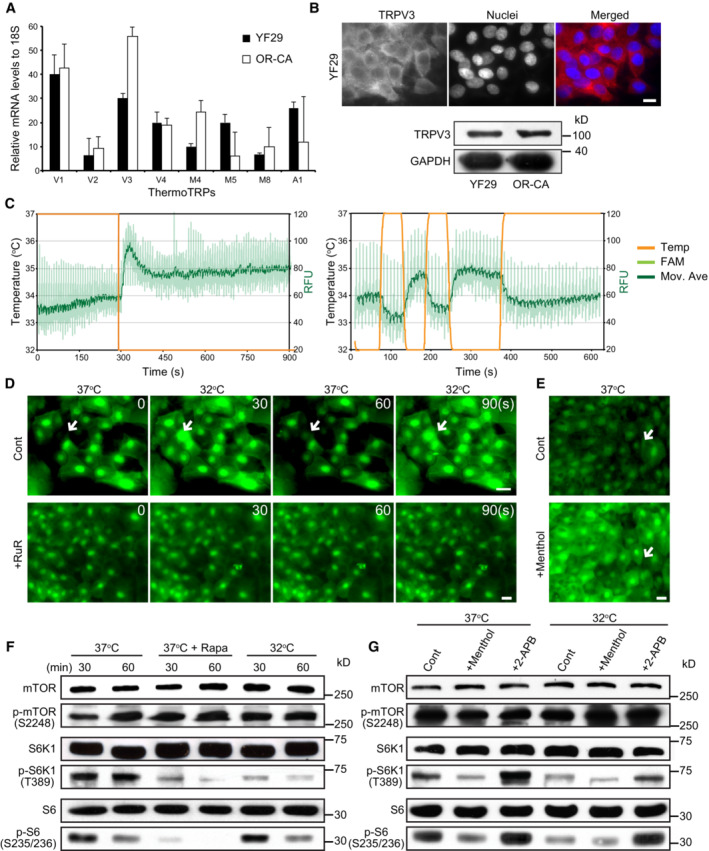
Cultured human keratinocytes express functional thermoTRP channels that regulate mTORC1 kinase activity AThe transcripts of eight thermoTRP channels were quantified by qPCR in two different strains of human diploid keratinocytes (YF29 and OR‐CA). Data are presented as the mean ± SEM (*n* = 3 biological replicates).BTRPV3 protein was detected in both strains using immunocytochemistry and western blotting. Scale bar = 10 μm.CThermoTRP functionality was assessed as described (Reubish *et al*, 2009). Briefly, cultured human keratinocytes were labeled with a fluorescent calcium dye (Fluo‐4) and exposed to brief temperature fluctuations using a RT–PCR machine (32–37°C and back to 32°C in 1‐min cycle). Two representative experiments demonstrate a significant increase in Fluo‐4 fluorescence when the temperature decreases.D, ELive imaging of cultured keratinocytes (YF29) labeled with Fluo‐4 in the presence of a TRPV antagonist (Ruthenium red (RuR) 500 μM) or agonist (menthol 500 μM) and subjected to brief temperature fluctuations from 37 to 32°C. The heating/cooling device and its calibration are shown in Fig EV2. The device was placed in a Zeiss inverted microscope equipped with a culture chamber with temperature set at 37°C and monitored with an independent thermo probe (SK‐L200 Datalogger Sato Japan). (D) Temperature change induced intracellular flux of calcium, which was blocked by treatment with RuR. Scale bars, 20 μm. The temperature was switched between 32 and 37°C every 30 s. (E) Menthol induces an intracellular flux of calcium. RuR and menthol were added to the keratinocyte cultures 1 h before starting the temperature changes. Arrows indicate keratinocytes in which the fluorescent signals significantly changed. Scale bar, 20 μm.F, GYF29 cells were cultured for 7 days at 37°C in a Nuaire 8700E culture incubator. Cells were then maintained at 37°C or switched to an adjacent incubator with temperature set at 32°C. Temperature was monitored with independent thermo probes (see Fig EV1) in the presence or absence of rapamycin (100 nM), or a cold agonist (menthol—500 μM), a warm agonist (2‐APB—200 μM) and the vehicle (control—ethanol 70%) for 30 and 60 min. (F) mTORC1 kinase activity decreases in the presence of rapamycin and when the temperature is switched from 37 to 32°C. (G) mTORC1 kinase activity decreases in the presence of menthol and increases in presence of 2‐APB, indicating that ThermoTRP and mTOR signaling are connected. Rapamycin (Rapa), menthol, and 2‐APB were added in the keratinocyte cultures 1 h before starting the temperature changes. Data information: The data shown were confirmed in two technical replicates. Source data are available online for this figure.

**Figure EV2:**
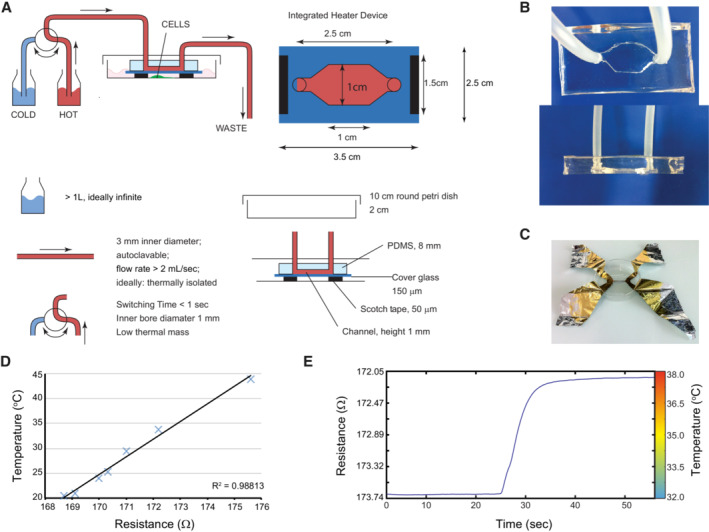
Custom‐made in‐house device to control temperature while imaging cultured keratinocytes in real‐time Schematic representation of the heating/cooling system. The device was designed to perfectly fit a 100 mm Petri dish and consists of a transparent polydimethylsiloxane (PDMS) chamber cast on a microscope slide cover glass. Two holes are pierced at each extremity of the chamber to allow connection of the chamber with the help of tubing to two separate bottles filled with water, immersed in individual water baths set at different temperatures. An external valve allows temperature variation in the chamber by switching the water flow between water baths.Top and side views of the PDMS chamber.Four‐point measurement of the electrical resistance of a thin aluminum foil was used to calibrate the device.The relation between temperature and resistance was always linear in the experimental temperature range.Example of the calibration curve of the device used for the imaging experiments is presented in Fig 2D and E.

Next, we determined whether cultured human keratinocytes could sense temperature through thermoTRP channels. All thermoTRP channels were transcribed to a certain extent by cultured human keratinocytes (YF29 and OR‐CA) when analyzed by quantitative PCR (Fig 2A). Additionally, we confirmed the expression of TRPV3 in cultured human keratinocytes by western blotting and immunocytochemistry (Fig 2B) TRPV3 is activated at approximately 37°C, the temperature of standard cell cultures (Peier *et al*, 2002; Moqrich *et al*, 2005; Venkatachalam & Montell, 2007). The functional activity of thermoTRP channels, which are calcium‐permissive channels, is commonly evaluated by measuring the fluorescence intensity of calcium‐sensitive dyes, such as Fluo‐4. We reasoned that a real‐time PCR (RT–PCR) machine designed to read out the fluorescence emission of laser‐excited fluorophores, together with a high level of temperature control, could be a valuable tool for determining thermoTRP activity (Reubish *et al*, 2009). YF29 cells were grown for 7 days and trypsinized. The suspended cells were loaded with Fluo‐4 dye before incubation into a 96‐well plate (10^5^ cells/well). The response of the cells to thermal stimulation was analyzed using RT–PCR machine. We observed that the amount of fluorescence, which reflects the intracellular calcium concentration, slightly decreased when the temperature quickly increased from 32 to 37°C, whereas it significantly increased when the temperature quickly dropped from 37 to 32°C (Fig 2C). Since these experiments did not provide information on the behavior of individual cells, we developed a custom‐made device that allowed real‐time imaging of thermally induced calcium flux in cultured cells (Fig EV2A–E). These experiments confirmed the results obtained by RT–PCR machine. Interestingly, the cell's response to temperature change was more homogenous after the second thermal stimulation (Fig 2D) suggesting sensitization of the channels. This phenomenon was driven by thermoTRP channels, because the addition of Ruthenium Red (RuR), a broad thermoTRP channel antagonist, prevented calcium influx (Fig 2D). In addition, the strong calcium influx observed after a decrease in temperature was reproduced using menthol, an agonist of “cool” thermoTRP channel TRPM8 at a 500 μM concentration (Fig 2E). Taken together, these experiments demonstrate that brief temperature fluctuations impacted on the intracellular calcium concentration of cultured human keratinocytes. These experiments confirmed that cultured human keratinocytes express functional thermo‐sensitive TRP channels and that modulation of the activity of these channels causes changes in the intracellular calcium concentration.

### ThermoTRP channel activation is connected to mTORC1 signaling

Calcium is involved in various intracellular signaling pathways; therefore, we investigated whether particular signaling pathways are activated or inactivated by temperature. We reasoned that mTORC1 signaling might be involved in the keratinocyte stem cell response to thermal stimuli because mTORC1 signaling integrates environmental cues, including pH, nutrients, and oxygen tension, and in turn, controls cell growth and proliferation (Liu & Sabatini, 2020). As shown in Fig 1, regulation of cell size and proliferation by temperature in human keratinocytes strongly suggests that mTORC1 signaling is involved in the temperature‐dependent processes. To test this hypothesis, we assessed the kinase activity of mTOR after thermal or chemical stimulation of thermoTRP channels. YF29 keratinocytes were cultured for 7 days at 37°C. On Day 7, the cells were moved from 37°C to 32°C for 30 or 60 min or exposed to the mTORC1 inhibitor rapamycin (100 nM) for the same time. As expected, short treatment with 100 nM rapamycin significantly decreased the phosphorylation levels of phosphorylated S6 kinase 1 (S6K1) and ribosomal protein S6 (S6), which are downstream targets of mTORC1 (Fig 2F). We also found that levels of phosphorylated S6K1 and S6 were significantly decreased when cells were moved from 37 to 32°C for 30 or 60 min, indicating that the temperature drop inhibited mTORC1 kinase activity (Fig 2F). Phosphorylated mTOR levels remained constant, regardless of the stimulus. Next, the cells were treated with 500 μM menthol (a TRPM8 agonist) for 1 h at 37 and 32°C. This resulted in a significant decrease in the levels of phosphorylated S6K1 and S6 under all conditions (Fig 2G). In contrast, treatment with 200 μM 2‐aminoethoxydiphenyl borate (2‐APB), an agonist of “hot” thermoTRP channels (TRPV1, 2, and 3), for 1 h at 37 and 32°C increased the phosphorylation of S6K1 and S6. Once again, the phosphorylation status of mTOR remained constant independently of the stimulus. The finding that treatment with 2‐APB increased mTORC1 kinase activity even at 32°C indicates that lower temperatures do not reduce the activity of chemical reaction but regulate mTORC1 activity through thermoTRP channels. Therefore, these experiments support our hypothesis that thermoTRP channels are connected to the mTOR signaling pathway through calcium influx.

**Figure EV3:**
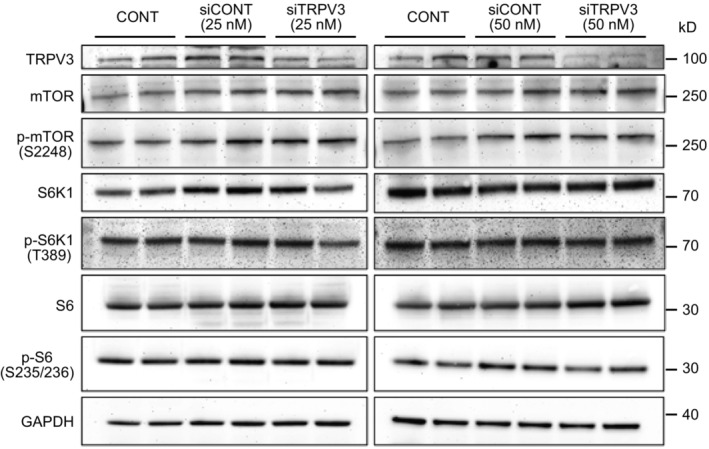
Downregulation of *TRPV3* gene expression does not affect mTORC1 kinase activity Human keratinocytes were treated with siRNA targeting *TRPV3* or control siRNA, and the expression and phosphorylation of mTOR, S6K1, and S6 were examined by western blotting. Keratinocytes were independently transfected with 25 or 50 nM siRNA targeting *TRPV3* and decreased expression of TRPV3 was also confirmed by western blotting. Notably, decreased expression of TRPV3 did not affect the mTORC1 kinase activity.Source data are available online for this figure.

Next, we examined whether the activity of TRPV3 is directly involved in mTORC1 signaling, as TRPV3 is activated at 33–39°C (Venkatachalam & Montell, 2007). Human keratinocytes were treated with siRNA targeting *TRPV3* and cultured at 37°C, and the mTORC1 signaling activity was examined by analyzing the phosphorylation levels of S6K1 and S6. Western blotting analysis revealed no changes in the phosphorylation levels of these proteins, although the expression of TRPV3 was significantly decreased (Fig EV3). This result strongly suggests that TRPV3 activity does not mainly contribute to the temperature‐dependent regulation of mTORC1 signaling and that the temperature drop cooperatively sensed through several types of thermoTRP channels inhibits mTORC1 signaling similar to rapamycin treatment.

**Figure 3:**
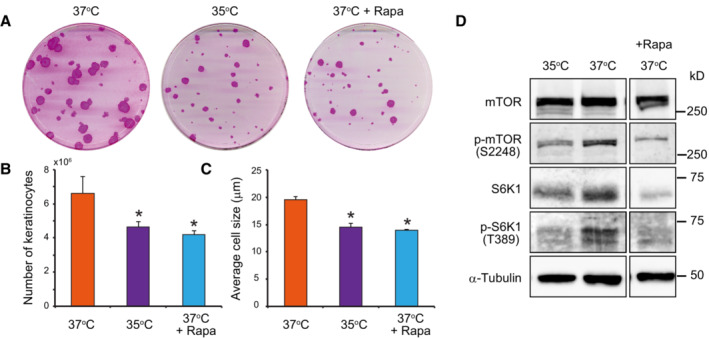
Lower temperatures and rapamycin decrease human keratinocyte stem cell growth and proliferation Colony‐forming efficiency (CFE) of keratinocytes was unaltered when keratinocytes were cultured at 35°C or in the presence of 100 nM rapamycin. However, the mean area of colonies was smaller when compared to cells cultured at 37°C.Culture at 35°C and treatment with rapamycin decreased keratinocyte proliferation as shown by the lower total number of cells after 7 days of culture compared with in the control condition.Culture at 35°C and treatment with rapamycin also reduced cell growth as shown by a smaller mean diameter of keratinocytes compared with that in the control condition after 7 days of culture.Long‐term exposure to temperatures below 37°C decreased mTORC1 kinase activity. Data information: In (B and C), data are presented as the mean ± SD (*n* = 4 biological replicates). *P < 0.05 (two‐tailed Student's *t*‐test). The data shown were confirmed in two technical replicates. Source data are available online for this figure.

### Lower temperature affects human keratinocyte stem cell growth and proliferation and decreases mTORC1 activity, similar to rapamycin

**Figure EV4:**
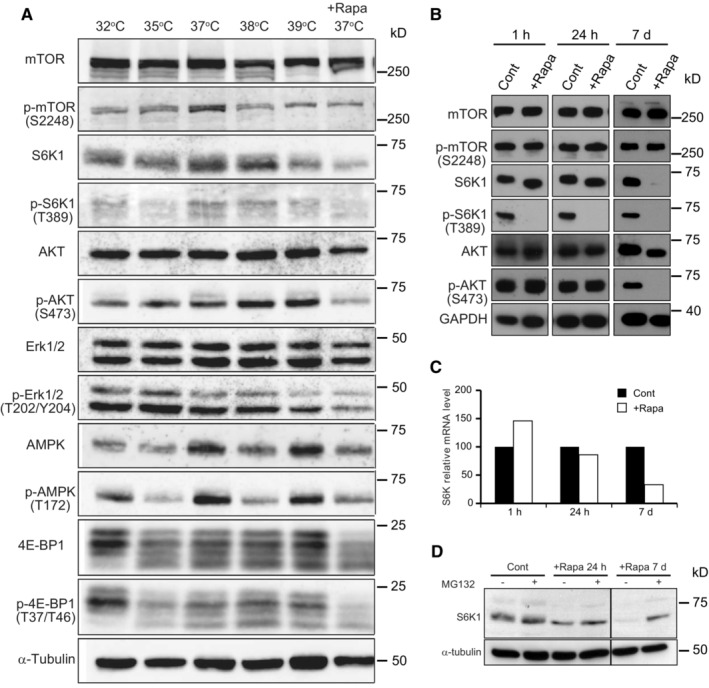
Long‐term exposure of human keratinocytes to temperatures below 37°C decreases mTORC1 kinase activity AYF29 keratinocytes were cultured for 7 days at 32, 35, 37, and 37°C in the presence of rapamycin (100 nM). Note that phosphorylation levels of ERK are completely independent of temperature, demonstrating that the decrease in phosphorylated S6K1 is not linked to a general effect of temperature on enzyme activity. Thermo Scientific Heracell 150 incubators with temperatures set at 32, 35, 36, 37, and 38°C.B–DYF29 keratinocytes were cultured for 7 days before proteins were extracted and expression of S6K1 was analyzed by western blotting and qPCR. Rapamycin (100 nM) was added either for the entire duration of the culture (7 days) or the last 24 h, or for the last 1 h. (B) As expected, a short rapamycin exposure inhibited S6K1 phosphorylation without affecting S6K1, whereas a long rapamycin exposure (7 days) significantly affected the detection of S6K1. (C) qPCR experiments indicate that the level of expression of S6K1 mRNAs decreases with long rapamycin exposure. (D) MG132 (1 μM), a specific proteasome inhibitor, was added to YF29 cells grown in the absence for the presence of rapamycin for a day (24 h) or for 7 days (7 d) before proteins were extracted. These experiments demonstrate that a decrease in expression and increase in protein degradation are responsible for the low levels of S6K1 after 7 days of rapamycin exposure to rapamycin. Source data are available online for this figure.

Next, we examined the effects of rapamycin on growth and proliferation of human keratinocyte stem cells. Human keratinocytes were cultured for 12 days at 35, 37°C, or in the presence of 100 nM rapamycin at 37°C. Keratinocytes cultured at 35°C and those cultures with rapamycin at 37°C formed smaller colonies than keratinocytes cultured at 37°C (Fig 3A). We also confirmed that the smaller size of the colonies was due to a decrease in both cell number and size using a Tali™ hemocytometer (Fig 3B and C). We further examined the effects of constant exposure to temperatures below 37°C on mTORC1 kinase activity. Human keratinocytes (YF29) were cultured for a week at 35°C and 37°C and harvested for western blot analysis. Phosphorylation of mTOR and S6K1 decreased at 35°C to levels comparable to those observed in rapamycin‐treated cells (Fig 3D). In addition, phosphorylation of Erk1/2 was not proportional to temperature, indicating that the decrease in mTORC1 activity was not related to a general reduction in cell activity (Fig EV4A). Furthermore, S6K1 and pS6K1 were barely detectable by western blot after 7 days of rapamycin exposure (Fig EV4B). Although the expression of S6K1 mRNAs was downregulated (Fig EV4C), the addition of MG132, a proteasome inhibitor, prevented the degradation of S6K1 (Fig EV4D), indicating that long‐term rapamycin treatment can affect the turnover of downstream targets of mTORC1 in keratinocyte stem cells. Altogether, these experiments demonstrated that a variation in temperature as small as two degrees Celsius impacted stem cell behavior through mTOR signaling.

**Figure 4:**
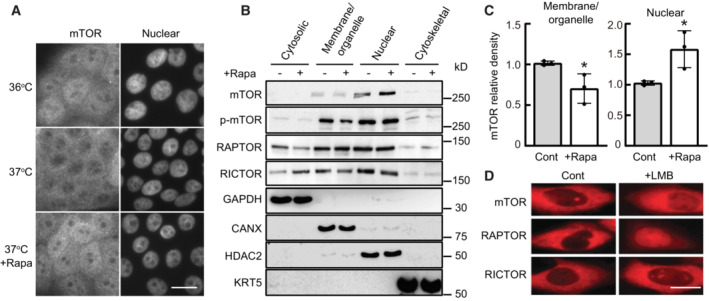
mTOR can translocate to the nucleus of human keratinocytes in response to rapamycin or a small drop in temperature Human keratinocytes (YF29) were cultured in individual Thermo Scientific Heracell 150 incubators with the temperature set at 36 or 37°C for 4 days and incubated with rapamycin (100 nM) for 4 h before they were immunostained with a primary rabbit polyclonal mTOR antibody (T2949 Sigma‐Aldrich) and with an Alexa Fluor 568 secondary antibody. mTOR nuclear signal was visible after rapamycin treatment or exposure to 36°C. Scale bar, 20 μm.Proteins were extracted from different YF29 subcellular components with ProteoExtract™ Subcellular Proteome Extraction kit (Calbiochem). The purity of each subcellular fraction was confirmed using specific markers, GAPDH for the cytosolic fraction, calnexin (CANX) for the membrane and organelles fraction, keratin 5 (KRT5) for the cytoskeletal fraction and HDAC2 for the nuclear fraction. mTOR, p‐mTOR, RICTOR, and RAPTOR were identified both in the membranes/organelles and nuclear fractions by western blotting. RICTOR and RAPTOR were also identified in the cytosolic fraction.Western blot bands were scanned and their density measured using Scion image software (Scion Corporation). This experiment indicated that exposure to rapamycin increased the level of nuclear mTOR approximately 1.5‐fold while decreasing the level of cytoplasmic mTOR. Data are presented as the mean ± SD (*n* = 3 biological replicates). *P < 0.05 (two‐tailed Student's *t*‐test).CHO cells engineered to transiently express SNAP‐mTOR, SNAP‐RAPTOR, and SNAP‐RICTOR (Gautier *et al*, 2008) were incubated with the nuclear export inhibitor leptomycin B (LMB; 6.4 ng/ml) for 6 h at 37°C. Cells were labeled with TMR‐Star (1 μM) for 30 min, washed and then imaged. This experiment demonstrated that mTOR, RAPTOR, and RICTOR accumulated in the nucleus after LMB treatment. Scale bars, 10 μm. Data information: The data shown were confirmed in three technical replicates. Source data are available online for this figure.

### Temperatures below 37°C and rapamycin induce mTOR nuclear translocation

Recent studies have revealed that mTOR complexes translocate to the nucleus and directly regulate transcription (Giguère, 2018; Torres & Holz, 2021). Next, we examined the effects of temperature and rapamycin on the translocation of the mTOR complexes. Immunostaining of cultured human keratinocytes with a primary rabbit polyclonal mTOR antibody revealed a nuclear signal for mTOR when the cells were cultured at 36°C for 4 days or exposed to 100 nM rapamycin for 4 h at 37°C. In contrast, the mTOR signal was mostly cytoplasmic in cells maintained at 37°C (Fig 4A). Thus, these experiments suggest that mTOR can translocate to the nucleus, as suggested for other mammalian cells (Zhang *et al*, 2002). Therefore, we performed western blot analysis of the subcellular fractions of cultured human keratinocytes with or without rapamycin treatment. These experiments confirmed that mTOR is present in the nuclear extracts of cultivated human keratinocyte stem cells (Fig 4B). Raptor and Rictor, the major subunits of mTORC1 and mTORC2, respectively, were also detected in the nuclear fraction, indicating that other members of the mTOR complex translocate to the nucleus. Quantification of the bands revealed that the amount of nuclear mTOR increased approximately 1.5‐fold after rapamycin treatment, whereas it decreased by the same proportion in the membrane/organelle fraction (Fig 4C). Next, we used SNAP‐tag technology optimized for Chinese hamster ovary (CHO) cells to demonstrate further the presence of mTOR and some of its associated proteins in the nucleus of mammalian cells (Keppler *et al*, 2003). CHO cells were transiently transfected with constructs encoding SNAP‐tag human proteins mTOR, Raptor, and Rictor and then treated with leptomycin B to inhibit nuclear export. As expected, mTOR was clearly observed in the nucleus. Interestingly, Raptor was almost exclusively observed in the nucleus, whereas Rictor remained largely cytoplasmic. The accumulation of mTOR and Raptor in the nucleus indicated that these two proteins could shuttle between the cytoplasmic/lysosomal surface and the nucleus (Fig 4D). Together, these results further support the observation that mTOR and some of its associated proteins can translocate to the nucleus (Zhang *et al*, 2002; Rosner & Hengstschläger, 2008; Vazquez‐Martin *et al*, 2011; Yadav *et al*, 2013).

### Low temperature and rapamycin similarly impact gene expression in cultured normal human keratinocytes

**Figure 5:**
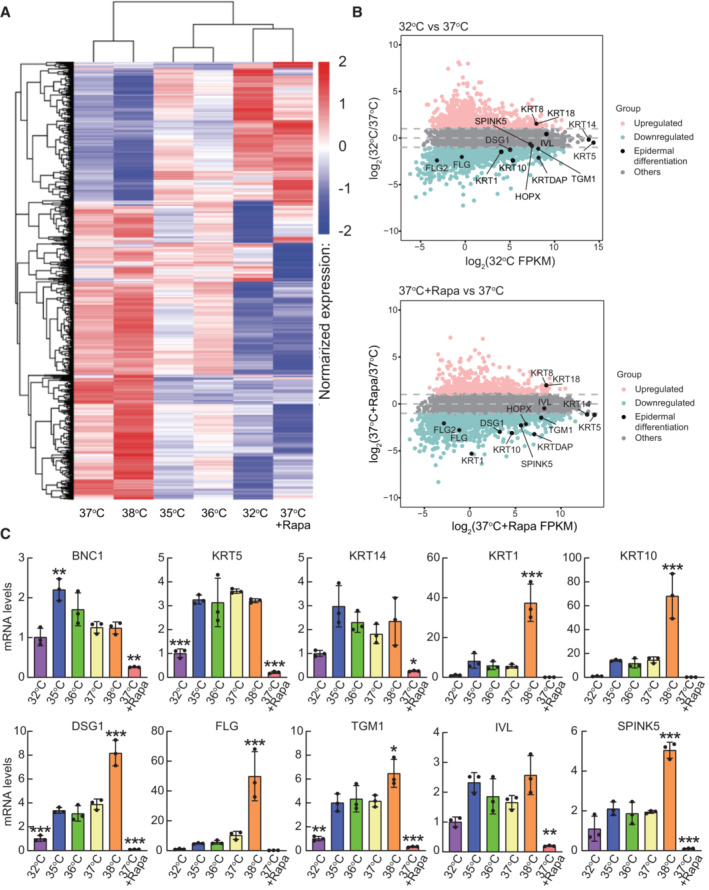
Temperature and rapamycin impact gene expression in cultured human keratinocytes A genome‐wide expression screen was performed in parallel on normal human epidermal keratinocytes (YF29) cultured for 10 days at 32, 35, 36, 37, 38, and 37°C in the presence of rapamycin (100 nM).
Cluster analysis of gene expression differences is shown by the log10 (FPKM+1) value. Red denotes genes with high expression levels, and blue denotes genes with low expression levels. The color range from blue to red represents the log10 (FPKM+1) value from small to large.MA plot shows all the genes without low expression or log_2_FC = 0. Genes indicated in pink: upregulated (≥ 1 log_2_(FC)), blue: downregulated (≤ −1 log_2_(FC)), gray: others (−1 < log_2_(FC) < 1) and black: epidermal differentiation genes. Log_2_(FC) values were calculated using the edgeR package.Quantitative PCR analysis of epidermal differentiation‐associated genes. Data are presented as the mean ± SD (*n* = 3 biological replicates). *P < 0.05, **P < 0.01, ***P < 0.001 (one‐way ANOVA with Dunnett's multiple comparisons test vs 37°C).

Next, we performed a comprehensive mRNA and miRNA expression analysis using RNA‐seq analysis to determine whether the observed nuclear translocation of mTOR could affect gene expression. 5 × 10^4^ normal human epidermal keratinocytes (YF29) were cultured for 10 days in T25 cm^2^ culture flasks with culture medium in individual incubators with temperatures set at 32, 35, 36, 37, and 38°C. 100 nM rapamycin was added to some of the flasks cultured at 37°C. The temperature in each incubator was constantly monitored using a data logger from either Gain Express Holdings Ltd. or AZ INSTRUMENT CORP, and the temperature in each incubator remained constant for the entire duration of the experiment. RNA was extracted from each culture condition using an RNeasy Mini Kit (QIAGEN). Library preparation and bioinformatic analyses were performed using Novogene‐AIT. A dataset accessible through the NCBI GEO Series (miRNA: GSE198696; mRNA: GSE199522) was subsequently obtained for each condition using the Illumina NovoHiSeq™ 6000 platform. The cluster analysis of gene expression revealed that one‐degree Celsius difference in temperature impacted global mRNA expression and that the addition of rapamycin to cells cultured at 37°C had a similar effect to that at 32°C (Fig 5A). Remarkably, differential gene expression analysis using DEGseq revealed that epidermal differentiation‐associated genes such as *KRTDAP*, *KRT1*, *KRT10*, *FLG*, *FLG2*, *SPINK5*, and *HOPX* were downregulated, whereas *KRT8* and *KRT18* which are expressed early during embryonic development of the epidermis were highly expressed at 32 and 37°C + rapamycin compared with those to 37°C (fold change (log_2_) > 1; Fig 5B). Quantitative PCR analysis confirmed that epidermal differentiation‐associated genes including *KRT1*, *KRT10*, *DSG1*, *FLG*, *TGM1*, *IVL*, and *SPINK5* were downregulated in cells cultured at 32 and 37°C + rapamycin and upregulated in cells cultured at 38°C (Fig 5C). Human keratinocyte cultures contain basal and stratified keratinocytes and also include keratinocytes expressing both basal and differentiation markers (Barrandon & Green, 1987; Nanba *et al*, 2013). This type of keratinocytes exhibits a growth‐arrested phenotype and is spontaneously generated under cell culture conditions (Roshan *et al*, 2016; Nanba *et al*, 2021). Expression of both basal cell markers including *KRT5* and *KRT14* and epidermal differentiation genes in keratinocytes cultured at 38°C indicates that higher temperatures significantly increased postmitotic basal keratinocytes.

**Figure EV5:**
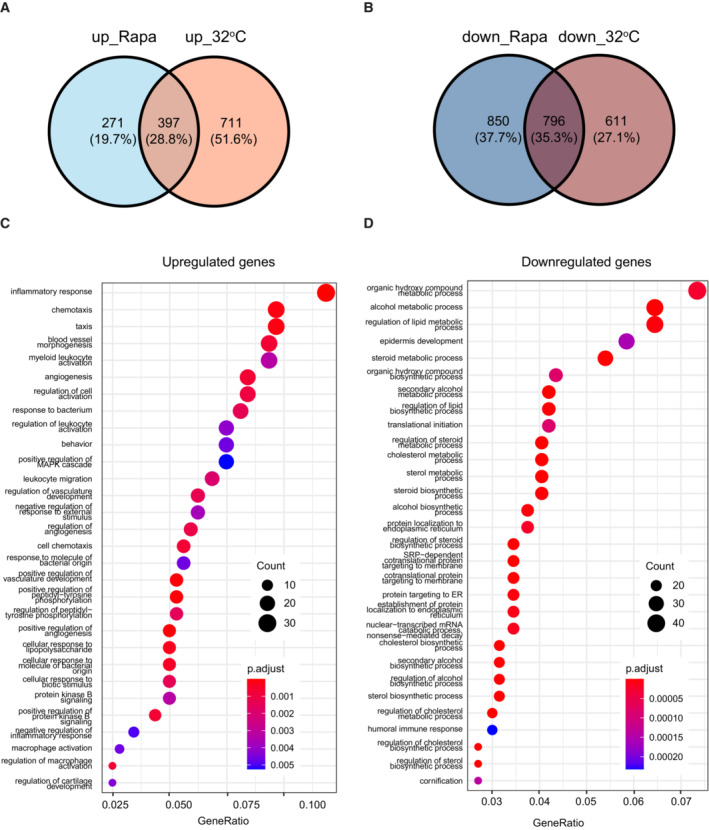
Analysis of mRNA expression in cultured human keratinocytes A, BVenn diagrams of upregulated (A) or downregulated (B) genes in keratinocytes cultured at 32°C or treated with 100 nM rapamycin compared with gene expression in the control culture (37°C).C, DGene ontology (GO) analysis of genes upregulated (C) or downregulated (D) genes under both conditions in keratinocytes cultured at 32°C or treated with 100 nM rapamycin compared with gene expression in the control culture (37°C).

RNA‐seq analysis also revealed that rapamycin and the 32°C environment increased the expression of 668 and 1,108 genes, respectively, compared with that of the control culture (37°C). Among them, 397 genes were upregulated under both conditions (Fig EV5A). Conversely, 1,646 and 1,407 genes were downregulated by rapamycin treatment and culture at 32°C, respectively, and 796 genes were downregulated under both conditions (Fig EV5B). We further performed gene ontology (GO) analysis of genes upregulated or downregulated by both rapamycin treatment and culturing at 32°C compared with the control culture (37°C). Although GO analysis did not indicate any particular GO terms related to stem cell maintenance among these upregulated genes (Fig EV5C), rapamycin treatment and the 32°C culture both downregulated the same genes related to many types of metabolic processes and protein translation (Fig EV5D), which are known to be regulated by mTORC1 signaling (Liu & Sabatini, 2020). GO analysis also confirmed that genes associated with “epidermal development” and “cornification” were downregulated by rapamycin treatment and culturing at 32°C (Fig EV5D). These results are consistent with a previous report that rapamycin suppresses human keratinocyte differentiation (DeTemple *et al*, 2016).

**Figure 6:**
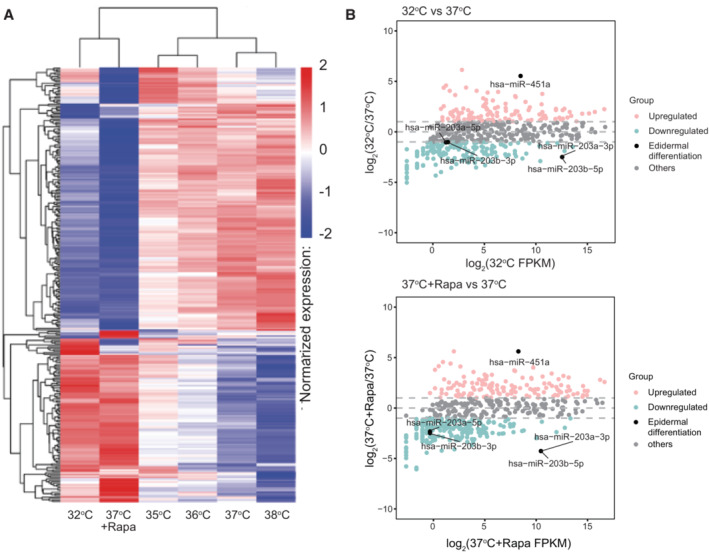
Temperature and rapamycin impact miRNA expression in cultured human keratinocytes Comprehensive miRNA expression was performed in parallel on normal human epidermal keratinocytes (YF29) cultured for 10 days at 32, 35, 36, 37, 38 and at 37°C in the presence of rapamycin (100 nM). miRNA expression levels were estimated by transcript per million (TPM) using the following criteria (Zhou *et al*, 2010): Normalization formula: normalized expression = mapped read count/total reads × 1,000,000.
Cluster analysis of miRNA expression differences using the log10 (TPM + 1) value. Red denotes miRNAs with high expression levels, and blue denotes miRNAs with low expression levels. The color range from blue to red represents the log10 (TPM + 1) value from small to large.The MA plot showing all miRNAs without low expression or log_2_FC = 0. miRNAs indicated in pink: upregulated (≥ 1 log_2_(FC)), blue: downregulated (≤ −1 log_2_(FC)), gray: others (−1 < log_2_(FC) < 1) and black: miRNAs related to epidermal differentiation. log_2_(FC) values are calculated using the edgeR package.

### Low temperature and rapamycin similarly impact miRNA expression in cultured normal human keratinocytes

One‐degree Celsius difference in temperature also affected miRNA expression and the cluster analysis demonstrated that the miRNA expression pattern in keratinocytes cultured at 32°C was a similar to that of keratinocytes cultured at 37°C with rapamycin (Fig 6A). Additionally, the analysis of miRNA expression with DEGseq of cells cultured at 32, 38, or 37°C + rapamycin revealed that miR‐451, which suppresses tumor cell growth through G1 cell cycle arrest (Sun & Jiang, 2018), was highly expressed at 32 and 37°C + rapamycin. In contrast, epidermal differentiation‐associated miRNAs such as miR‐203, which are highly expressed in the upper basal layer and contribute to skin re‐epithelization (Viticchiè *et al*, 2012), were upregulated at warmer temperatures (37 and 38°C, fold change (log_2_) > 1) (Fig 6B). These results strongly support the notion that rapamycin maintains a basal and stem cell‐like phenotype, while repressing stratification. Taken together, these results support the idea that a decrease in temperature to 32°C affected gene expression, similar to continuous exposure to rapamycin.

### Continuous inhibition of mTORC1 delays clonal conversion

**Figure 7:**
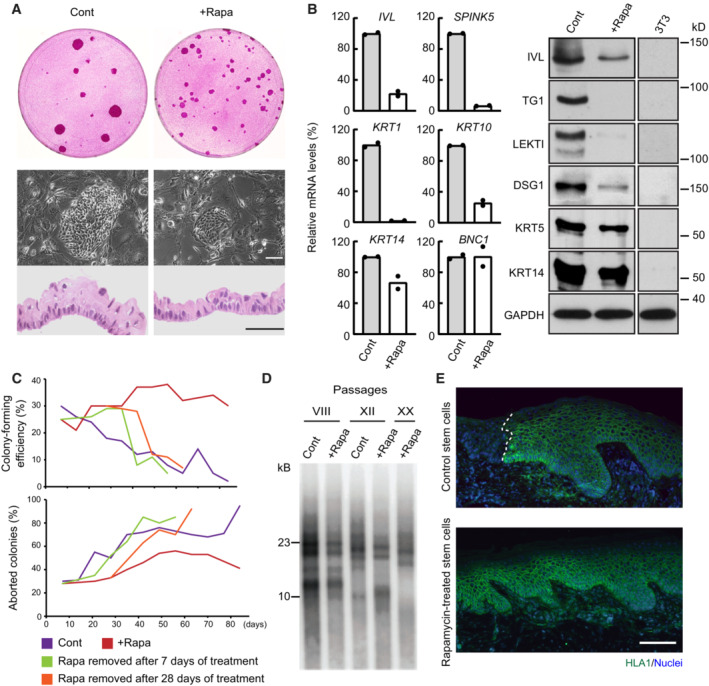
Inhibition of mTORC1 favors the maintenance of a stem cell phenotype in culture Cultured human keratinocytes (YF29 passage VII) treated with rapamycin (100 nM) formed smaller colonies and a thinner epithelium when confluent. Scale bars, 50 μm.Cultured human keratinocytes (YF29 passage III) treated with rapamycin maintained a basal phenotype as shown by the expression of several basal (*KRT14*, *BNC1*) and terminal differentiation markers (*SPINK5*, *IVL*, *KRT1*, *and KRT10*) using quantitative PCR (left panel) and western blotting (right panel). Data are presented as the mean (*n* = 2 biological replicates).A holoclone (Barrandon & Green, 1987) was isolated from YF29 strain and serially passaged once a week for more than 13 weeks in the presence or absence of rapamycin (100 nM). A colony‐forming efficiency was performed at each passage for each condition to determine the number of growing and aborted colonies. Rapamycin significantly increased the number of progressively growing colonies while decreasing the number of terminally differentiated (aborted) colonies, demonstrating a drastic reduction in clonal conversion. Nevertheless, clonal conversion resumed when rapamycin was withdrawn after 7 or 28 days of culture.Telomere length was analyzed on DNA extracted from YF29 keratinocytes with or without rapamycin treatment at passages VIII, XII, and XX (keratinocytes grown without rapamycin did not reach passage XX because of clonal conversion—see panel C). Telomere shortening could be observed through passages even if keratinocytes were continuously treated with rapamycin.Rapamycin‐treated keratinocytes (YF29 passage IV) can fully regenerate an epidermis when transplanted onto SCID mice; grafts were harvested after 71 days and immunostained with an HLA‐1 antibody to identify the human cells (white dotted line indicates the boundary between mouse and human epidermis). Scale bar, 100 μm. Data information: The data shown were confirmed in two technical replicates. Source data are available online for this figure.

Cultured human keratinocyte stem cells (holoclones) can spontaneously convert into cells with restricted growth potential (meroclones and paraclones), a phenomenon known as clonal conversion, which is independent of telomere‐linked senescence and is favored by inappropriate culture conditions or an inadequate microenvironment (Barrandon & Green, 1987; Barrandon *et al*, 2012; Nanba *et al*, 2013). Hence, the capacity to maintain stem cells by reducing clonal conversion to a minimum is critical for successful cell and *ex vivo* gene therapies (Mavilio *et al*, 2006; Droz‐Georget Lathion *et al*, 2015; Hirsch *et al*, 2017). In this study, we found that lower temperatures and mTORC1 inhibition maintained human keratinocyte stem cells *ex vivo*. To apply our findings to regenerative medicine, we further assessed the effects of decreased mTORC1 signaling with rapamycin on human keratinocyte stem cell behavior, as rapamycin is already used in clinics worldwide as an immunosuppressive drug (MacDonald, 2001), and the conventional cell culture systems present a difficulty for precise temperature control. YF29 cells were cultured in the presence of 100 nM rapamycin for various time periods. Rapamycin treatment did not affect cell attachment and colony formation but had a striking effect on colony growth, confirming previous observations (Javier *et al*, 1997). Microscopically, rapamycin‐treated cells formed smaller, more transparent, and less stratified colonies than the control cultures (Fig 7A). Human keratinocyte stem cells generate a stratified epithelial sheet similar to the epidermis when the culture becomes confluent and can be used for autologous transplantation of cultured epidermal sheets when detached from the culture vessel with dispase (Green *et al*, 1979). We also found that confluent rapamycin‐treated keratinocytes formed a thinner epithelium than untreated cells, suggesting an effect on differentiation (Fig 7A). This hypothesis was confirmed by the decreased expression of several terminal differentiation markers in the epidermis (Fig 7B). Since rapamycin‐treated keratinocytes maintained a basal phenotype did not stratify and did not express epidermal terminal differentiation markers, we speculated that mTORC1 inhibition could impact stem cell maintenance and clonal conversion. Thus, we isolated holoclones from YF29 culture (passage III), as described by Barrandon & Green (1987). Holoclones were serially passaged once a week for more than 13 weeks while exposed to 100 nM rapamycin during each feeding (every 3–4 days). Rapamycin‐treated keratinocytes maintained a high and constant colony‐forming efficiency (CFE; approximately 40%), whereas the CFE of untreated cells decreased with each passage (Fig 7C upper panel). Furthermore, rapamycin‐treated keratinocytes maintained a high ratio of progressively growing colonies to terminally aborted colonies for the entire duration of the experiment (13 weeks; Fig 7C lower panel). Remarkably, the CFE and the ratio of growing colonies quickly decreased following rapamycin withdrawal to reach the levels of control keratinocytes. Therefore, we assumed that rapamycin did not affect the overall lifespan of the cells, which was confirmed by the fact that telomeres shortened at a similar rate in rapamycin and non‐rapamycin‐treated cells (Fig 7D). These results indicate that continuous inhibition of mTORC1 signaling favors the maintenance of the stem cell phenotype by reducing clonal conversion without stopping telomere‐linked stem cell aging. Next, we transplanted cultured epithelial grafts into immunodeficient mice (Droz‐Georget Lathion *et al*, 2015) to evaluate the capacity of stem cells grown in the presence or absence of rapamycin to generate a self‐renewing epidermis. Long‐term exposure of keratinocyte stem cells to rapamycin did not affect the capacity of the cells to generate an epidermis (Fig 7E), indicating that rapamycin‐treated keratinocyte stem cells can also be used for autologous transplantation of cultured epidermal sheets. Taken together, these results support the notion that inhibition of mTOR signaling delays clonal conversion of epidermal stem cells without affecting their ability to form an epidermis when transplanted.

## Discussion

Of all the tissue stem cells, keratinocyte stem/progenitor cells of the skin are the most exposed to external temperature fluctuations. Our results demonstrate that temperature is part of the keratinocyte stem cell niche and that human keratinocyte stem/progenitor cells sense minor variations in temperature through thermo‐sensitive cationic channels connected to mTORC1 signaling.

Although the anatomical niche for interfollicular epidermal keratinocyte stem cells is still debated (Potten, 1974; Ghazizadeh & Taichman, 2001, 2005; Clayton *et al*, 2007; Mascre *et al*, 2012; Rompolas *et al*, 2016; Sada *et al*, 2016), the expression of molecules associated with epidermal stemness, including integrin α2, α3, and β1 subunits, and type XVII collagen, is observed in basal cells where the dermis is closest to the skin surface, known as the dermal papillae in human skin (Jones *et al*, 1995; Wang *et al*, 2020). Hence, basal keratinocytes are more sensitive to environmental temperature changes than keratinocytes located in other regions of the basal layer or epidermal appendages. Interestingly, a temperature decrease from 37 to 32°C is sufficient for human keratinocyte stem/progenitor cells to retain a basal phenotype and decrease differentiation, strongly indicating that temperature is an intrinsic part of the physical niche for epidermal stem cells. This may also explain why mouse cells can be cultured at 32–33°C together with a low calcium medium to retain a basal phenotype (Betz *et al*, 1992; Caldelari *et al*, 2000; Jensen *et al*, 2010).

mTOR signaling integrates information on the cellular environment and determines cellular behavior (Liu & Sabatini, 2020). Recently, the TRPV1‐mTORC1 signaling axis through calcium influx has been shown to be involved in muscle hypertrophy (Ito *et al*, 2013). Here, we have demonstrated that keratinocyte stem cells can sense temperature changes through the thermoTRP channels‐mTORC1 signaling axis and change their behavior. TRP channels regulate neurons through changes in membrane excitability (Moran *et al*, 2011). Electrical signals also control keratinocyte behavior through PI3K signaling (Zhao *et al*, 2006). PI3K signaling is located upstream of mTOR signaling, which can also explain the thermoTRP channels‐mTORC1 signaling axis. In this study, we demonstrated the nuclear translocation of mTOR in a cooler environment and rapamycin treatment. RNA‐sequence analysis has also revealed similarities in global gene expression patterns between cooler environments and rapamycin treatment. This finding suggests that inhibition of mTORC1 signaling induces nuclear translocation of mTOR complexes that directly regulate transcription (Giguère, 2018). However, it remains unclear whether nuclear mTOR is involved in regulating keratinocyte behavior, and further investigations are necessary.

mTORC1 signaling regulates the behavior of hematopoietic stem cells (Yilmaz *et al*, 2006; Zhang *et al*, 2006; Kharas *et al*, 2010), intestinal stem cells (Yilmaz *et al*, 2012), satellite cells (Rodgers *et al*, 2014; Yue *et al*, 2016, 2017), and hair follicle stem cells (Castilho *et al*, 2009). mTOR is also essential for skin morphogenesis and epidermal barrier function (Ding *et al*, 2016). mTORC1 inhibition results in small cell size in mammalian cells (Fingar *et al*, 2002), and mTORC1 activity is also associated with epidermal and oral mucosa keratinocyte size (Kim *et al*, 2006; Izumi *et al*, 2009). In this study, we demonstrated that the culturing keratinocytes at cooler temperatures decreased cell size, similar to treatment with rapamycin. Importantly, small keratinocytes are highly clonogenic (Barrandon & Green, 1985). Therefore, the inhibition of mTORC1 signaling by a cooler environment or rapamycin treatment favors stem cell maintenance in human keratinocyte cultures. We have demonstrated here that rapamycin treatment prevented clonal conversion without modulating telomerase activity and that keratinocyte stem cells treated with rapamycin possess tissue regenerative capacity. Hence, an artificial niche for cultured keratinocyte stem cells may be designed by controlling mTOR signaling. Furthermore, during cell transplantation, keratinocyte stem/progenitor cells abruptly switch from a pampered cell culture environment to a hostile grafting bed, possibly with deleterious consequences. Manipulating mTOR signaling may facilitate the adaptation of stem cells to a new microenvironment and, consequently, enhance engraftment. This can have major clinical implications since keratinocyte stem cells are used to transplant extensive burn wounds (Gallico *et al*, 1984; Pellegrini *et al*, 1999; Ronfard *et al*, 2000) or to treat patient whose vision is severely impaired by corneal stem cell deficiency (Pellegrini *et al*, 1997).

### Conclusions

mTOR appears to be a key modulator of the response of stem cells to a variety of physical or chemical signals (temperature, microwaves, pH, hypoxia, varying levels of nutrients and growth factors), together with its regulatory role in translation and capacity to potentially fine‐tune the transcription of a vast number of developmentally important genes. One can envision the develop of new therapeutic approaches by designing new drug that interfere with mTORC1 signaling. This is of paramount importance given the great potential of stem cell therapy and the implication of mTOR signaling in a variety of diseases, such as cancer, diabetes, cardiovascular diseases, neurodegenerative diseases, and skin and ocular diseases.

## Materials and Methods

### Temperature monitoring in animals

IPTT‐300 transponders (BioMedic Data Systems) were implanted subcutaneously into the backs of mice and rats using sterile trocars provided by the manufacturer. Temperature monitoring was initiated 2 weeks after implantation to avoid measuring wound healing‐related temperature variations. The subcutaneous temperature was recorded every 30 min for 24 h periods using a DAS‐7006/7 s reader system (BioMedic Data Systems).

### Temperature monitoring in cultures

Cells were cultured in individual Thermo Fisher Scientific Heracell 150 or Nuaire 8700E incubators. Importantly, the doors of the incubators were not opened for the entire duration of the experiments (7–10 days). The temperature in each incubator was monitored using thermal probes connected to an SK‐L200 Datalogger instrument (Sato, Japan) and remained constant for the duration of the experiments. Alternatively, the temperature was recorded using a data logger from Gain Express Holdings Ltd or AZ INSTRUMENT CORP (reference 88160 Temp.) with comparable results. To measure the temperature directly in the culture media, we implemented a miniature low‐power wireless temperature sensor comprising an MSP430F2274 microcontroller (http://www.ti.com/product/msp430f2274), and a TMS37157 PaLFI (passive low‐frequency interface) (http://www.ti.com/product/tms37157) from Texas Instruments. The RFID base station (ADR2) reader included in the eZ430‐TMS37157 demo kit (http://www.ti.com/tool/ez430‐tms37157) provided power for the wireless sensor and MSP430F2274. The temperature was measured using a constant positive temperature coefficient (PTC) sensor integrated into an MSP430F2274 Analog‐to‐Digital Converter (ADC) and powered by a constant current from the internal reference. As gain (mV/°C) and offset (mV@0°C) varied across different individual MSPs, a calibration was needed for each unit. After 1‐point calibration, the typical accuracy of the sensor was ± 0.3°C.

### Cell culture

Human keratinocytes were isolated from the foreskin of a newborn (YF29) or the groin of a 42‐year‐old woman (OR‐CA) and cultured on a feeder layer of irradiated 3T3‐J2 cells, as described previously (Rochat *et al*, 1994; Ronfard *et al*, 2000). Cells were used between passages III and XII and cultured in individual Thermo Fisher Scientific Heracell 150 or Nuaire 8700E incubators. Single keratinocytes were isolated under an inverted microscope using a Pasteur pipette as described previously (Barrandon & Green, 1987), and clones were subcultured once a week (Rochat *et al*, 1994). To evaluate the colony‐forming ability of cultured keratinocytes, 100–200 cells were seeded onto irradiated 3T3‐J2 cells in 100 mm Petri dishes. The cultures were fixed and stained after 12 days, and the number of progressively growing and aborted colonies were counted under a binocular microscope. 3T3‐J2 cells were cultured in DMEM supplemented with 10% bovine serum.

### Chemicals used in cell culture

Rapamycin (Calbiochem or Sigma‐Aldrich), the TRPV3 agonists 2‐APB (2‐aminoethoxydiphenyl borate), and menthol (Sigma‐Aldrich) were diluted in 70% ethanol. The TRPV3 antagonist RuR (Ruthenium Red—Calbiochem) was diluted in distilled water. Leptomycin B (Sigma‐Aldrich) was diluted in methanol. MG132 (Sigma‐Aldrich) was diluted in dimethyl sulfoxide (DMSO). The calcium indicator Fluo‐4 Direct™ (Invitrogen) was diluted in the buffer provided by the manufacturer.

### Transplantation of cultured epithelium

A fibrin‐based matrix containing RDEB fibroblasts was prepared as described previously (Larcher *et al*, 2007). Next, 10^5^ keratinocytes were seeded on top of the fibrin gel, grown to confluence in culture medium supplemented with 150 IU/ml aprotinin (Trasylol, Bayer) and transplanted onto the back of 8–10‐week‐old Fox‐Chase SCID mice (Charles River Laboratories) as described previously (Droz‐Georget Lathion *et al*, 2015). The mice were handled according to the Canton de Vaud veterinarian guidelines (authorization 2033). The grafts were harvested after 10 weeks and processed for histology and immunocytochemistry. To confirm the human origin of the regenerated epidermis, sections were immunostained for human HLA class I (1:500—SM2012P Acris) using standard protocols.

### Immunostaining

For immunohistochemistry (IHC), the entire tissues were fixed in 4% paraformaldehyde (PFA) for 2 h at room temperature (RT), embedded in OCT compound (Tissue‐Tek) and frozen in cold methyl‐butane. Ten micrometers of tissue sections were cut with a Leica CM3000 cryostat. For immunocytochemistry (ICC), human keratinocytes were cultured on glass coverslips seeded with lethally irradiated 3T3‐J2 in 12‐well culture plates and fixed after 7–10 days in 4% PFA or ice‐cold methanol, depending on the manufacturer's instructions. For all procedures, nonspecific sites were blocked for 45 min at RT with 5% goat serum/PBS solution or with 5% bovine serum albumin (BSA)/ PBS solution with or without 0.5% Triton X‐100 (blocking buffer). Tissue sections and cells were incubated overnight with primary antibodies (Appendix Table S1) diluted in blocking buffer at 4°C. After washing with PBS, the bolts were incubated for 1 h at RT with secondary antibodies Alexa‐Fluor568 or Alexa‐Fluor488 conjugated (Molecular Probes, Invitrogen) diluted 1:500 in PBS. Nuclear counterstaining was performed with Hoechst 33342 (Molecular Probes).

### Western blotting

After 7 days of culture, the dishes were placed on ice, and the cells were washed twice with ice‐cold PBS. The cells were then mechanically scraped, collected into microtubes, and centrifuged at 1,500 *g* for 5 min at 4°C. Cell pellets were lysed in 1% Triton X‐100, 5 mM EDTA, and a cocktail of protease and phosphatase inhibitors (Roche) in PBS for 45 min on ice. Samples were then centrifuged at 15,000 *g* for 5 min at 4°C, and the supernatant was collected on ice. The total protein concentration in each sample was determined using the Pierce BCA Protein Assay Kit (Thermo Scientific). Laemmli buffer containing 10% 2‐mercaptoethanol was then added to each sample (v/v), which was fractionated by SDS‐polyacrylamide gel electrophoresis (PAGE) before being transferred to a nitrocellulose membrane (Whatman). The membranes were then incubated with primary antibodies and probed with the respective secondary antibodies (Appendix Table S1).

### Transfection of siRNAs into normal human epidermal keratinocytes

siRNAs were transfected into human keratinocytes using Lipofectamine RNAiMAX reagent (Invitrogen) as described previously (Nanba *et al*, 2021). Briefly, the prepared RNAiMAX liposome solution containing 2 μl of RNAiMAX and 2 μl of 10 μM siRNA in 500 μl Opti‐MEM solution (Gibco) was immediately added into a well of a 6‐well plate after inoculation. The cells were then analyzed ≥ 24 h after the transfection. The siRNAs used in this study were COL17A1 siRNA (human; Santa Cruz Biotechnology; sc‐44170) and MISSION siRNA universal negative control #1 (Sigma‐Aldrich; SIC‐001).

### Detection of mTOR, RAPTOR, and RICTOR in fractioned cell lysates

YF29 cells were cultivated on a feeder layer of irradiated 3T3‐J2 cells for 3 days with or without 100 nM rapamycin before they were quickly scraped from the culture dish in a small volume of PBS. The cells were then fractionated using the ProteoExtract™ Subcellular Proteome Extraction kit (Calbiochem). Ten micrograms of protein from each subcellular fraction was dissolved in SDS sample buffer and separated by 7% SDS–PAGE before being transferred to nitrocellulose membranes (Schleicher & Schuell). After blocking with 5% skim milk in PBS, the membranes were immunoblotted overnight at 4°C with primary antibodies (Appendix Table S1). Membranes were then washed with 0.05% Tween‐20 in PBS and incubated with horseradish peroxidase (HRP)‐conjugated anti‐rabbit or anti‐mouse antibodies (Jackson ImmunoResearch, ref. 115‐035‐003 or 111‐035‐0035) for 1 h at room temperature. The membranes were then washed with 0.05% Tween‐20 in PBS, treated with an enhanced chemiluminescence substrate for HRP detection (Pierce) for 5 min, and exposed to Kodak BioMAX MR films. Bands were scanned, and their density was measured using Scion Image software (Scion Corporation). The values (mean ± SD) were determined from triplicate experiments. *P*‐values were obtained using Student's *t*‐test.

### Calcium imaging

Calcium levels were evaluated with the calcium indicator Fluo‐4 Direct™ (Invitrogen) using either a 7900 RT–PCR real‐time PCR machine (Life Technologies—Applied Biosystems) as described previously (Reubish *et al*, 2009) or with a custom‐made real‐time imaging device (Fig EV2).

#### Real‐time PCR setup

Human keratinocytes (YF29 and OR‐CA strains) were cultured in T25 flasks for 7–10 days before being dissociated with a mixture of 0.05% trypsin and 0.1% EDTA for 5–10 min at 37°C. The cells were then centrifuged and suspended in Fluo‐4 loading solution according to the manufacturer's guidelines (Invitrogen). The reagent volumes were calculated to obtain a density of 1 × 10^6^ cells/ml. Fifty‐microliter aliquots of the cell/Fluo‐4 mix were then distributed into 96‐well plates and incubated for 1 h at 37°C. The amplitude and duration of each temperature cycle were adjusted using SDS 4.0 software (Life Technologies, Applied Biosystems), and the amount of fluorescence was then measured using the FAM (6‐carboxyfluorescein) channel of an ABI 7900 RT–PCR machine (Life Technologies—Applied Biosystems).

#### Real‐time imaging device

Human keratinocytes (YF29 and OR‐CA strains) were cultured in 100 mm Petri dishes for 7–10 days before they were loaded with Fluo‐4 according to the manufacturer's guidelines (Invitrogen). The cells were incubated for 1 h at 37°C before the custom‐made device was placed on top, and the fluorescent cells were imaged under a Zeiss Axioplan microscope. The heating/cooling system is described in Fig EV2.

### SNAP‐tag

mTOR, RICTOR, and RAPTOR SNAP‐tag plasmids were designed and transfected into CHO cells as described previously (Gautier *et al*, 2008). To insert SgfI and PmeI sites at the C terminus of SNAP, SNAP‐p53 in pSEMS1‐SNAP‐p53 was replaced with SNAP cDNA containing SgfI and PmeI sites via EcoRI and XhoI. mTOR cDNA cut out from pF1KB1123 (Kazusa DNA institute, Japan) was further inserted into this intermediate vector via the SgfI and PmeI sites, yielding pSEMS1‐SNAP‐mTOR. SNAP cDNA was inserted into prk5‐HA‐RAPTOR (Addgene, USA) via the EcoRI and SalI sites, to generate prk5‐SNAP‐RAPTOR. To insert the SalI and NotI sites at C terminus of SNAP, myc‐RICTOR in prk5‐myc‐RICTOR (Addgene, USA) was replaced with SNAP cDNA containing the SalI and NotI sites via the EcoRI and NotI sites. RICTOR cDNA cut out from prk5‐myc‐RICTOR was further inserted into this intermediate vector via the SalI and NotI sites, yielding prk5‐SNAP‐RICTOR. Chinese hamster ovary (CHO) cells were cultured in DMEM F12 (Cambrex) supplemented with 5% fetal bovine serum (Cambrex) in a humidified atmosphere under 5% CO_2_. Cells were seeded on a 35 mm micro dish (Ibidi) to a density of 8 × 10^4^ cells per dish, 24 h before transfection. Transient transfections were performed using the FuGENE‐6 transfection reagent (Roche) following the manufacturer's instructions. After 24 h of transfection, the cells were treated with 6.4 ng/ml leptomycin B (LMB; Sigma‐Aldrich), a specific inhibitor of nuclear export receptor Crm1, for 7 h. To visualize SNAP‐tagged proteins, TMR‐Star, a fluorescent substrate for SNAP‐tag (NEB, USA), was added to a final concentration of 1 mM, and cells were incubated for 15 min and washed with medium (5 min × 3, 20 min). The localization of SNAP‐tagged proteins was examined under a Zeiss Axiovert 200 inverted microscope, equipped with an objective lens LD Plan‐Neofluar 63×/0.75 corr Ph2, an AxioCamMR digital camera (Zeiss), and Zeiss filter sets (excitation BP 545/25; emission BP 605/70).

### Quantitative PCR

cDNAs were synthesized from 5 ng of total RNA using SuperScript III reverse transcriptase (Invitrogen) and hexamer random primers (Promega), according to the manufacturers' instructions. cDNAs were adjusted to equal levels by PCR amplification using primers for 18S and GAPDH. Primers listed in Appendix Tables S2 and S3 were designed to ensure the uniqueness of each gene using LightCycler Probe Design 1.0 software and NCBI BLAST (http://www.ncbi.nlm.nih.gov/BLAST/). Quantitative PCR was performed using the LightCycler FastStart DNA Master SYBR Green I reagents and a LightCycler System piloted by a LightCycler 3.5 software (Roche Diagnostics). Differences between the experimental samples and controls were calculated using the 2^−ΔΔCT^ method (Nicolas *et al*, 2003).

### RNA‐seq analysis for mRNA

Library preparation and bioinformatic analysis were performed using Novogene‐AIT. mRNA sequencing of conditioned YF29 was performed using an Illumina NovaSeq™ 6000 platform. HTSeq v0.6.1 was used for data analysis to count the read numbers mapped to each gene. Fragments Per Kilobase of transcript sequence per million base pairs (FPKM) of each gene was calculated based on the length of the gene and read counts mapped to this gene. FPKM, the expected number of Fragments Per Kilobase of transcript sequence per million base pairs sequenced, considers the effect of sequencing depth and gene length for the read count at the same time and is currently the most commonly used method for estimating gene expression levels (Trapnell *et al*, 2010). The log_10_ (FPKM+1) value was used for the cluster analysis of gene expression differences. Red denotes genes with high expression levels, and blue denotes genes with low expression levels. The color range from blue to red represents the log_10_ (FPKM+1) value from small to large. Before differential gene expression analysis, the read counts were adjusted using the edgeR program package through one scaling normalized factor, for each sequenced library. Differential expression analysis under the two conditions was performed using the DEGseq R package (1.20.0). *P*‐values were adjusted using the Benjamini–Hochberg method. Corrected *P*‐values of 0.005 and log_2_ (fold change) of 1 were set as the thresholds for significantly differential expression. Genes upregulated (397) or downregulated (796) genes in cells under both rapamycin treatment and culture at 32°C were obtained using differentially expressed genes extracted by the R package edgeR (version 3.32.1) with default settings except for dispersion value (bcv) = 0.2 following the package instructions for the case when biological replication experiments were not performed. The number of genes was displayed using the R package ggvenn (version 0.1.9). For these genes upregulated or downregulated under both conditions, GO analysis was performed by R package clusterProfiler (version 3.18.1), with pvalueCutoff = 0.01, qvalueCutoff = 0.05 and ont (ontology term) = “Biological Process.”

### RNA‐seq analysis for miRNA

Library preparation and bioinformatic analysis were performed using Novogene‐AIT. miRNA sequencing of conditioned YF29 was performed using an Illumina NovaSeq™ 6000 platform. miRNA expression levels were estimated by transcript per million (TPM) using the following criteria (Zhou *et al*, 2010): Normalization formula: normalized expression = mapped read count/Total reads × 1,000,000. The log_10_ (TPM + 1) value was used for cluster analysis of miRNA expression differences. Red denotes miRNAs with high expression levels, and blue denotes miRNAs with low expression levels. The color range in from blue to red represents the log_10_ (TPM + 1) value from small to large. Differential expression analysis of the two samples was performed using DEGseq (2010) R package. *P*‐values were adjusted using the q‐value (Storey, 2003). Q‐value < 0.01 and |log_2_ (foldchange)| > 1 was set as the threshold for significant differential expression by default.

### Preparation of RNA samples and procedure for comprehensive mRNA expression screen

Total RNA was extracted from cultured YF29 cells using the RNeasy® Mini Kit (QIAGEN) and treated with DNase I (RNase‐free DNase Set; QIAGEN) according to the manufacturer's recommendations. For RNA quantification and qualification, degradation and contamination were monitored on 1% agarose gels. RNA purity was assessed using a NanoPhotometer^®^ spectrophotometer (IMPLEN, CA, USA). RNA integrity and quantification were assessed using the RNA Nano 6000 Assay Kit of the Bioanalyzer 2100 system (Agilent Technologies, CA, USA). After checking the RNA quality, to prepare the library for transcriptome sequencing, a total amount of 1 μg RNA per sample was used as input material for RNA sample preparation. Following the manufacturer's recommendations, sequencing libraries were generated using the NEBNext^®^ Ultra™ RNA Library Prep Kit for Illumina^®^ (NEB, USA), and index codes were added to attribute the sequences to each sample. Briefly, mRNA was purified from the total RNA using poly‐T oligo‐attached magnetic beads. Fragmentation was performed using divalent cations under elevated temperatures in NEBNext First‐strand Synthesis Reaction Buffer (5×). First‐strand cDNA was synthesized using random hexamer primer and M‐MuLV Reverse Transcriptase (RNase H). Second‐strand cDNA synthesis was subsequently performed using DNA Polymerase I and RNase H. Remaining overhangs were converted into blunt ends via exonuclease/polymerase activity. After adenylation of the 3′ ends of the DNA fragments, NEBNext Adaptor with a hairpin loop structure was ligated to prepare for hybridization. To preferentially select cDNA fragments of 150–200 bp in length, the library fragments were purified with the AMPure XP system (Beckman Coulter, Beverly, USA). Then, 3 μl USER Enzyme (NEB, USA) was used with size‐selected, adaptor‐ligated cDNA at 37°C for 15 min followed by 5 min at 95°C before PCR. PCR was performed using Phusion High‐Fidelity DNA polymerase, Universal PCR primers and Index (X) Primer. Finally, PCR products were purified (AMPure XP system), and library quality was assessed using the Agilent Bioanalyzer 2100 system. After the library preparation, clustering and sequencing were performed. The clustering of the index‐coded samples was performed on a cBot Cluster Generation System using the PE Cluster Kit cBot‐HS (Illumina) according to the manufacturer's instructions. After cluster generation, the library preparations were sequenced on an Illumina platform and 125/150 bp paired‐end reads were generated. Except RNA extraction, sample preparation, sequencing, and data analysis were performed at the Novogene Experimental Department.

### Preparation of RNA samples and procedure for comprehensive miRNA expression screen

Total RNA was extracted from cultured YF29 cells using the RNeasy® Mini Kit (QIAGEN) and treated with DNase I (RNase‐free DNase Set; QIAGEN) according to the manufacturer's recommendations. For RNA quantification and qualification, degradation and contamination were monitored on 1% agarose gels. RNA purity was assessed using a NanoPhotometer^®^ spectrophotometer (IMPLEN, CA, USA). RNA integrity and quantification were assessed using the RNA Nano 6000 Assay Kit of the Bioanalyzer 2100 system (Agilent Technologies, CA, USA). After checking the RNA quality, to prepare the library for small RNA sequencing, a total amount of 3 μg total RNA per sample was used as input material for the small RNA library. Following the manufacturer's recommendations, sequencing libraries were generated using NEBNext^®^ Multiplex Small RNA Library Prep Set for Illumina^®^ (NEB, USA.), and index codes were added to attribute the sequences to each sample. Briefly, the NEB 3′ SR Adaptor was directly and specifically ligated to the 3′ end of miRNA, siRNA, and piRNA. After the 3′ ligation reaction, the SR RT Primer was hybridized to an excess of 3′ SR Adaptor (which remained free after the 3′ ligation reaction), transforming the single‐stranded DNA adaptor into a double‐stranded DNA molecule. This step is important to prevent adaptor‐dimer formation as dsDNAs are not substrates for ligation mediated by T4 RNA Ligase 1 and therefore do not ligate to the 5′ SR Adaptor in the subsequent ligation step. The 5′ end adapter was ligated to the 5′ ends of the miRNAs, siRNA, and piRNA. First‐strand cDNA was synthesized using M‐MuLV Reverse Transcriptase (RNase H‐). PCR amplification was performed using LongAmp Taq 2X Master Mix, SR Primer for Illumina, and index (X) primer. The PCR products were purified on an 8% polyacrylamide gel (100 V, 80 min). DNA fragments corresponding to 140–160 bp (the length of small noncoding RNA plus the 3′ and 5′ adaptors) were recovered and dissolved in 8 μl of elution buffer. Finally, library quality was assessed using the Agilent Bioanalyzer 2100 system with DNA High Sensitivity Chips. After library preparation, clustering and sequencing were performed. Clustering of the index‐coded samples was performed on a cBot Cluster Generation System using the TruSeq SR Cluster Kit v3‐cBot‐HS (Illumina) according to the manufacturer's instructions. After cluster generation, the library preparations were sequenced on an Illumina platform and 50 bp single‐end reads were generated. Except for RNA extraction, the sample preparation, sequencing, and data analysis were performed at the Novogene Experimental Department.

### RNA‐sequence data analysis

For the MA plot, genes with low expression and the exact negative binomial test were filtered using the R Bioconductor package edgeR (version 3.32.1).

## Author contributions

**Daisuke Nanba:** Conceptualization; data curation; supervision; funding acquisition; validation; investigation; visualization; writing – original draft; writing – review and editing. **Jun‐Ichi Sakabe:** Conceptualization; data curation; formal analysis; validation; investigation; visualization; writing – original draft. **Johannes Mosig:** Data curation; formal analysis; investigation; visualization; writing – original draft. **Michel Brouard:** Conceptualization; investigation; visualization. **Fujio Toki:** Data curation; formal analysis; investigation. **Mariko Shimokawa:** Data curation; formal analysis; writing – review and editing. **Mako Kamiya:** Investigation; methodology. **Thomas Braschler:** Investigation. **Fahd Azzabi:** Investigation. **Stéphanie Droz‐Georget Lathion:** Investigation; methodology. **Kai Johnsson:** Supervision; methodology. **Keya Roy:** Investigation. **Christoph D Schmid:** Formal analysis. **Jean‐Baptiste Bureau:** Investigation. **Ariane Rochat:** Investigation; visualization. **Yann Barrandon:** Conceptualization; supervision; funding acquisition; writing – original draft; project administration; writing – review and editing.

## Disclosure and competing interests statement

The authors declare that they have no conflict of interest.

## Supporting information



Appendix

Expanded View Figures PDF

Source Data for Expanded View

PDF+

Source Data for Figure 2

Source Data for Figure 3

Source Data for Figure 4

Source Data for Figure 7

## Data Availability

The GEO accession number for RNA‐seq analysis datasets are GSE199522 for mRNA (https://www.ncbi.nlm.nih.gov/geo/query/acc.cgi?acc=GSE199522) and GSE198696 for miRNA (https://www.ncbi.nlm.nih.gov/geo/query/acc.cgi?acc=GSE198696).
