# Supplementary material for: Low temperature and mTOR inhibition favor stem cell maintenance in human keratinocyte cultures
Source: EMBO Rep. 2023 May 4;24(6):e55439. doi: 10.15252/embr.202255439 (PMC10240198; doi:10.15252/embr.202255439)
Supplement: Supplementary file 1 — Appendix [file EMBR-24-e55439-s009.pdf]

Appendix

A table of content

Appendix Table S1. List of antibodies for immunocytochemistry and Western blot (p2)

Appendix Table S2. List of primers used for quantitative PCR (p3)

Appendix Table S3. TaqMan probes for real time quantitative PCR (p4)

Appendix Table S1. List of antibodies for immunocytochemistry and Western blot

Protein	Manufacture	Catalog number
4E-BP1	Cell Signaling	9644
Phospho-4E-BP1 (T37/T46)	Cell Signaling	2855
AMPK	Cell Signaling	5831
Phospho-AMPK (T172)	Cell Signaling	2535
Akt	Cell Signaling	9272
Phospho-Akt (Ser473)	Cell Signaling	4058
Calnexin	BD Biosciences	610523
Desmoglein1	Santa Cruz	SC-20114
Erk1/2	Cell Signaling	9102
Phospho-Erk1/1(T202/Y204)	Cell Signaling	9101
GAPDH	Abcam	ab8245
HDAC2	Abcam	ab7029
Involucrin	gift from Dr. F. Watt	SY-5
Keratin5	Abcam	ab24647
TGM1	gift from Dr. F. Watt	BC1
Keratin14	Covance	PRB155P
LEKTI	Zymed	39-0500
mTOR	Sigma-aldrich	T2949
Phospho-mTOR (Ser2448)	Biosource	44-1125G
S6K1	Cell Signaling	9202
Phospho-S6K1 (Thr389)	Cell Signaling	9206
S6	Cell Signaling	2217
Phospho-S6 (Ser235/236)	Cell Signaling	4858
RAPTOR	Abcam	ab26264
RICTOR	Abcam	ab32892
TRPV3	Abcam	ab32734
$\alpha-$Tubulin	Sigma-aldrich	T6199

Appendix Table S2. List of primers used for quantitative PCR

Gene	Forward primer (5'-3')	Reverse primer (5'-3')
BNC1	GGAGCAGAGACAGACAC	CTGCCCATTCGACTTG
IVL	AGGTCCAAGACATTCAAC	CCCTTGTATGAGACGAT
KRT1	GCCCTACTTTGAGTCAT	GTCGAGACTGCGGTTG
KRT10	TCAGATCGACAATGCC	ACCAGTGGACACATTT
KRT14	CTTCCGCACCAAGTAT	ATACTGGTCACGCATC
S6K1	ATTTATTGGCAGCCCACGAACACC	TCCACAGGTGTCTGAGGATTTGCT
SPINK5	CAAGATGGAAGACTCGG	CACTCCTCACTTGGTT
TBP1	ATAATCCCAAGCGGTT	ATAATCCCAAGCGGTT
TRPV1	TTCTTCCAGTGTCTGCCTGA	CATCATCAACGAAGACCCG
TRPV2	CGAAGCCGAAAAGGAAGAC	TCCAGCACACAGGCATCTAC
TRPV3	GCTTGGAGAAGACAGGAGGA	GAGATCACCCCCACAAAGAA
TRPV4	TGAAGACTTTGAGGATGGGG	CGTCACCACTCCAGTGACAA
TRPM4	TTCCCCTGGACTACAACTACTC	TCTCTATTCGCGTCAACATCTTC
TRPM5	AGCATTTCTCTTGGGAGGACA	GCTCGAAGTCATACACGGTGA
TRPM8	GTGAAAGCGACTTGGTGAATTTT	GTGGCCTTGGAATCTTTGGTAA
TRPA1	CCAGGGCGTTGTCTATGAGG	CCAGGGCGTTGTCTATGAGG

Appendix Table S3. TaqMan probes for real-time quantitative PCR

Gene name	Catalog number
BNC1	Hs00231074_m1
KRT5	Hs00361185_m1
KRT14	Hs00265033_m1
KRT1	Hs00196158_m1
KRT10	Hs00166289_m1
DSG1	Hs00355084_m1
FLG	Hs00856927_g1
TGM1	Hs00165929_m1
IVL	Hs00846307_s1
SPINK5	Hs00928570_m1
GAPDH	Hs02786624_g1
