# Supplementary material for: Low temperature and mTOR inhibition favor stem cell maintenance in human keratinocyte cultures
Source: EMBO Rep. 2023 May 4;24(6):e55439. doi: 10.15252/embr.202255439 (PMC10240198; doi:10.15252/embr.202255439)
Supplement: Supplementary file 2 — Expanded View Figures PDF [file EMBR-24-e55439-s007.pdf]

Expanded View Figures

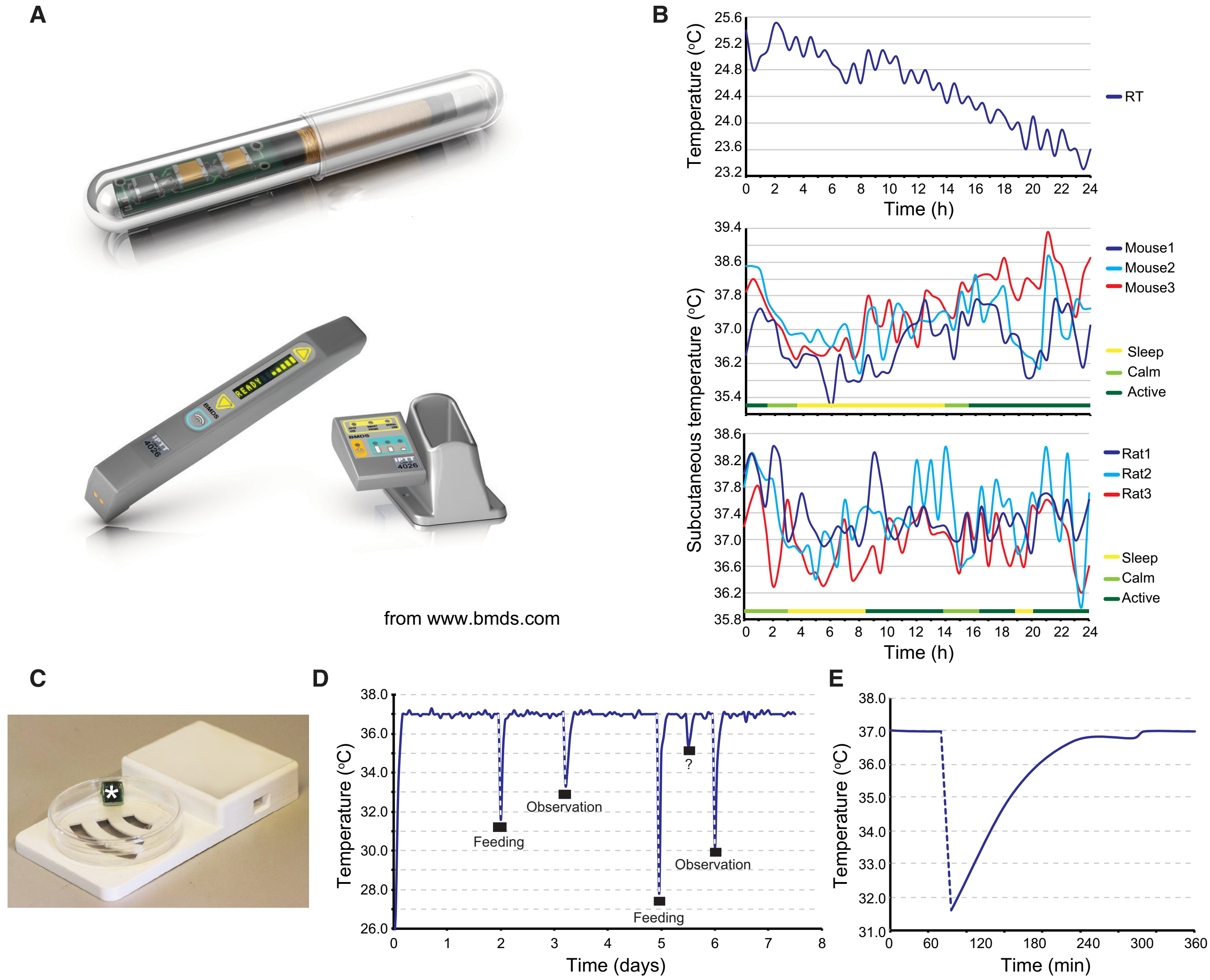

Figure EV1. Keratinocyte stem cells are constantly exposed to temperature variations *in vivo* and *in vitro*.

A Implantable IPTT-300 transponder and its DAS-7006/7 s reader system from BioMedic Data Systems, Seaford DE, USA (www.bmds.com). Actual size of the implantable transponder: length 14 mm and diameter 2 mm.

B Subcutaneous temperature of mice and rats constantly fluctuates. Three mice and three rats were each implanted subcutaneously with an IPTT-300 transponder (BioMedic Data Systems); the temperature and the behavior (active, calm, sleep) of each animal were then monitored over a 24 h. Room temperature (RT) was simultaneously monitored with a nonimplanted transponder.

C Picture of an EPFL-designed submersible RFID temperature sensor and its reading base station (Laboratory of Microengineering for Manufacturing). The culture vessel was a 100 mm size Petri dish.

D Cultured human keratinocytes were subjected to sharp temperature fluctuations when removal from the culture incubator (Nuaire 8700E) for medium change (feeding) or microscopic observation. Temperature of the culture medium was recorded over a week using immersed temperature sensors. Note that an unknown event (opening of the incubator door?) occurred between the fifth and sixth day of culture.

E Zoom of the temperature curve at the first medium change shown in B demonstrates that recovery to the set temperature (37°C) takes more than 2 h.

 © 2023 The Authors

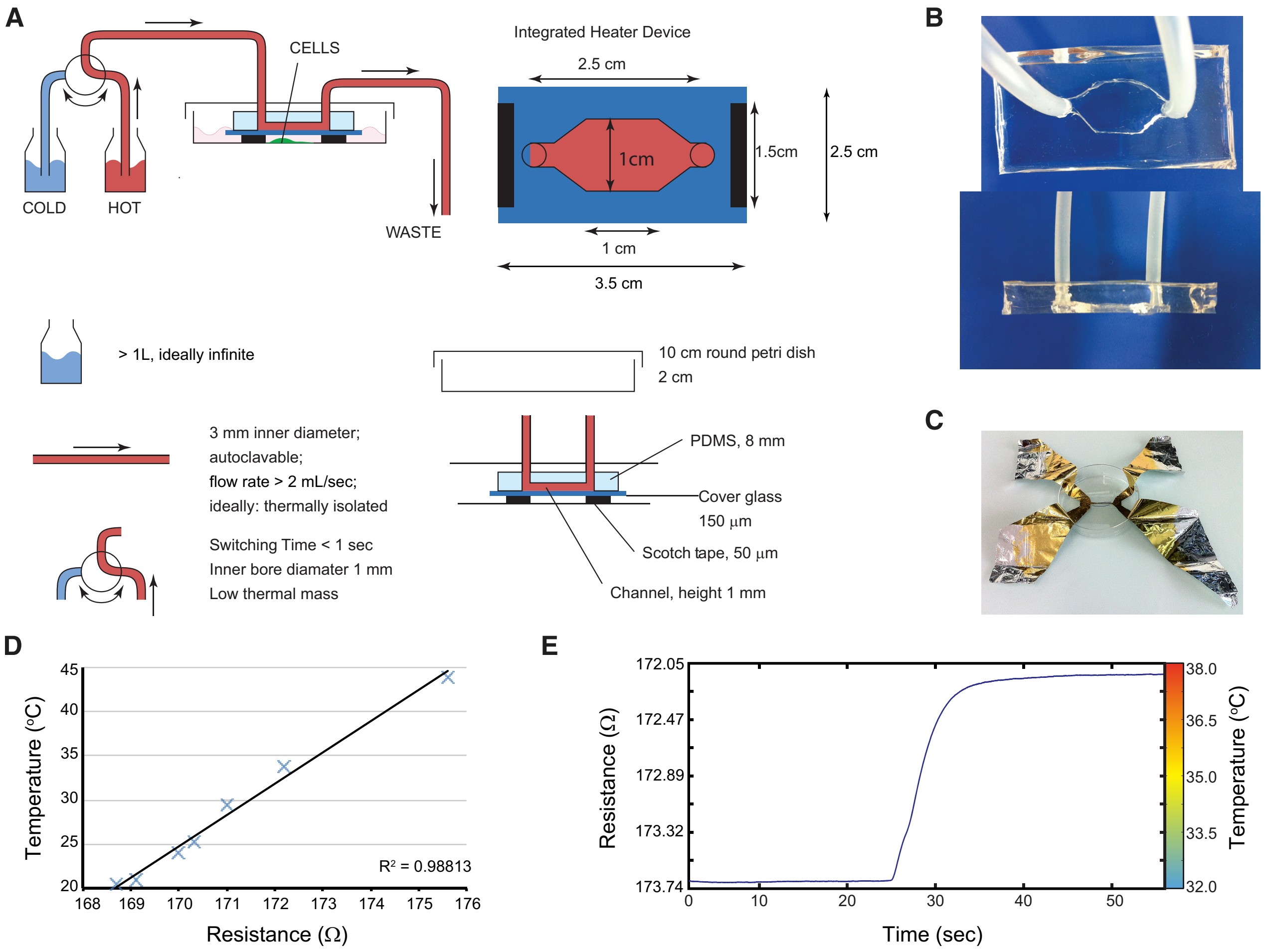

Figure EV2. Custom-made in-house device to control temperature while imaging cultured keratinocytes in real-time.

- A Schematic representation of the heating/cooling system. The device was designed to perfectly fit a 100 mm Petri dish and consists of a transparent polydimethylsiloxane (PDMS) chamber cast on a microscope slide cover glass. Two holes are pierced at each extremity of the chamber to allow connection of the chamber with the help of tubing to two separate bottles filled with water, immersed in individual water baths set at different temperatures. An external valve allows temperature variation in the chamber by switching the water flow between water baths.
- B Top and side views of the PDMS chamber.
- C Four-point measurement of the electrical resistance of a thin aluminum foil was used to calibrate the device.
- D The relation between temperature and resistance was always linear in the experimental temperature range.
- E Example of the calibration curve of the device used for the imaging experiments is presented in Fig 2D and E.

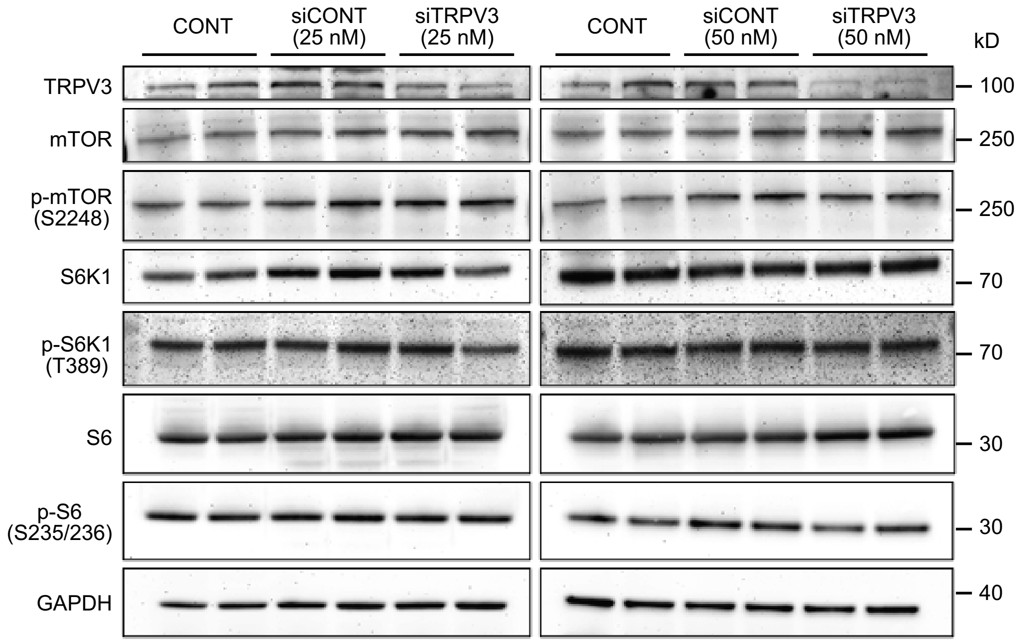

Figure EV3. Downregulation of *TRPV3* gene expression does not affect mTORC1 kinase activity.

Human keratinocytes were treated with siRNA targeting *TRPV3* or control siRNA, and the expression and phosphorylation of mTOR, S6K1, and S6 were examined by western blotting. Keratinocytes were independently transfected with 25 or 50 nM siRNA targeting *TRPV3* and decreased expression of TRPV3 was also confirmed by western blotting. Notably, decreased expression of TRPV3 did not affect the mTORC1 kinase activity.

Source data are available online for this figure.

 © 2023 The Authors

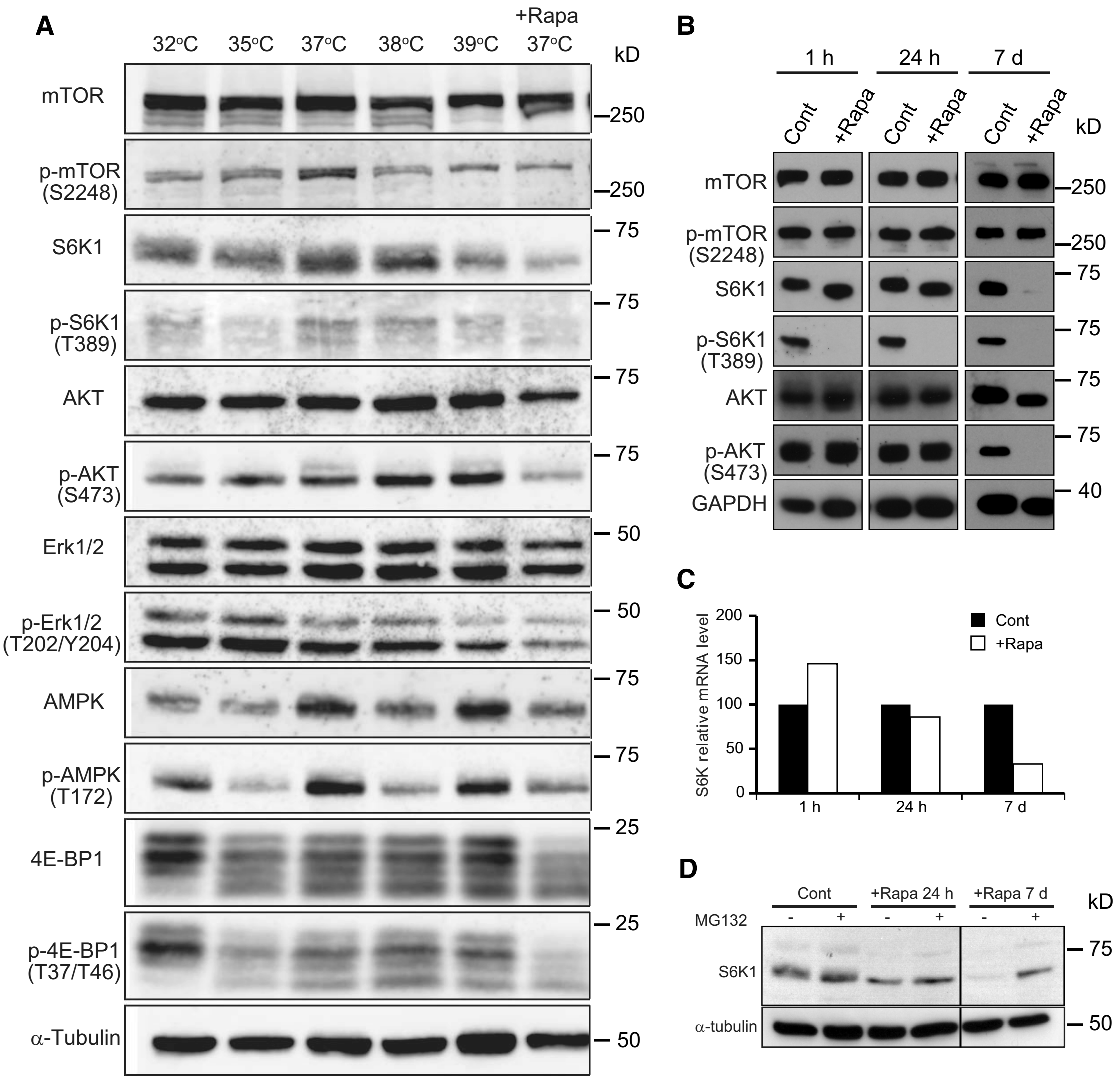

Figure EV4. Long-term exposure of human keratinocytes to temperatures below 37°C decreases mTORC1 kinase activity.

A YF29 keratinocytes were cultured for 7 days at 32, 35, 37, and 37°C in the presence of rapamycin (100 nM). Note that phosphorylation levels of ERK are completely independent of temperature, demonstrating that the decrease in phosphorylated S6K1 is not linked to a general effect of temperature on enzyme activity. Thermo Scientific Heracell 150 incubators with temperatures set at 32, 35, 36, 37, and 38°C.

B–D YF29 keratinocytes were cultured for 7 days before proteins were extracted and expression of S6K1 was analyzed by western blotting and qPCR. Rapamycin (100 nM) was added either for the entire duration of the culture (7 days) or the last 24 h, or for the last 1 h. (B) As expected, a short rapamycin exposure inhibited S6K1 phosphorylation without affecting S6K1, whereas a long rapamycin exposure (7 days) significantly affected the detection of S6K1. (C) qPCR experiments indicate that the level of expression of S6K1 mRNAs decreases with long rapamycin exposure. (D) MG132 (1 μM), a specific proteasome inhibitor, was added to YF29 cells grown in the absence for the presence of rapamycin for a day (24 h) or for 7 days (7 d) before proteins were extracted. These experiments demonstrate that a decrease in expression and increase in protein degradation are responsible for the low levels of S6K1 after 7 days of rapamycin exposure to rapamycin.

Source data are available online for this figure.

© 2023 The Authors

Figure EV5. Analysis of mRNA expression in cultured human keratinocytes.

A, B Venn diagrams of upregulated (A) or downregulated (B) genes in keratinocytes cultured at 32°C or treated with 100 nM rapamycin compared with gene expression in the control culture (37°C).

C, D Gene ontology (GO) analysis of genes upregulated (C) or downregulated (D) genes under both conditions in keratinocytes cultured at 32°C or treated with 100 nM rapamycin compared with gene expression in the control culture (37°C).

© 2023 The Authors

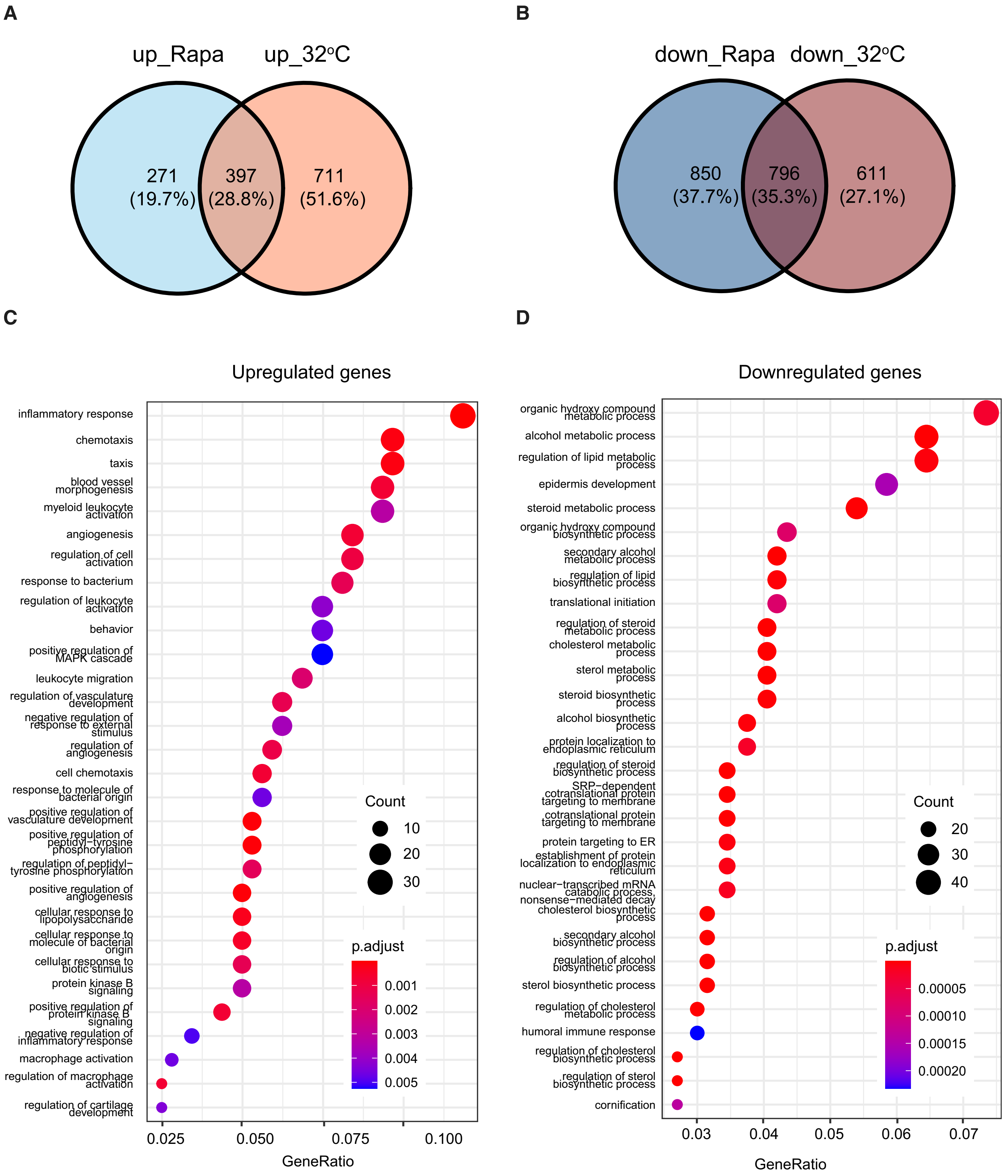
A
up_Rapa
up_32°C
271
(19.7%)
397
(28.8%)
711
(51.6%)
B
down_Rapa
down_32°C
850
(37.7%)
796
(35.3%)
611
(27.1%)
C
Upregulated genes
inflammatory response
chemotaxis
taxis
blood vessel morphogenesis
myeloid leukocyte activation
angiogenesis
regulation of cell activation
response to bacterium
regulation of leukocyte activation
behavior
positive regulation of MAPK cascade
leukocyte migration
regulation of vasculature development
negative regulation of response to external stimulus
regulation of angiogenesis
cell chemotaxis
response to molecule of bacterial origin
positive regulation of vasculature development
positive regulation of peptidyl−tyrosine phosphorylation
regulation of peptidyl−tyrosine phosphorylation
positive regulation of angiogenesis
cellular response to lipopolysaccharide
cellular response to molecule of bacterial origin
cellular response to biotic stimulus
protein kinase B signaling
positive regulation of protein kinase B signaling
negative regulation of inflammatory response
macrophage activation
regulation of macrophage activation
regulation of cartilage development
Count
10
20
30
p.adjust
0.001
0.002
0.003
0.004
0.005
0.025
0.050
0.075
0.100
GeneRatio
D
Downregulated genes
organic hydroxy compound metabolic process
alcohol metabolic process
regulation of lipid metabolic process
epidermis development
steroid metabolic process
organic hydroxy compound biosynthetic process
secondary alcohol metabolic process
regulation of lipid biosynthetic process
translational initiation
regulation of steroid metabolic process
cholesterol metabolic process
sterol metabolic process
steroid biosynthetic process
alcohol biosynthetic process
protein localization to endoplasmic reticulum
regulation of steroid biosynthetic process
SRP−dependent cotranslational protein targeting to membrane
cotranslational protein targeting to membrane
protein targeting to ER
establishment of protein localization to endoplasmic reticulum
nuclear−transcribed mRNA catabolic process, nonsense−mediated decay
cholesterol biosynthetic process
secondary alcohol biosynthetic process
regulation of alcohol biosynthetic process
sterol biosynthetic process
regulation of cholesterol metabolic process
humoral immune response
regulation of cholesterol biosynthetic process
regulation of sterol biosynthetic process
cornification
Count
20
30
40
p.adjust
0.00005
0.00010
0.00015
0.00020
0.03
0.04
0.05
0.06
0.07
GeneRatio

Figure EV5.
