# Supplementary figures and images for: Low temperature and mTOR inhibition favor stem cell maintenance in human keratinocyte cultures
Source: EMBO Rep. 2023 May 4;24(6):e55439. doi: 10.15252/embr.202255439 (PMC10240198; doi:10.15252/embr.202255439)

**A**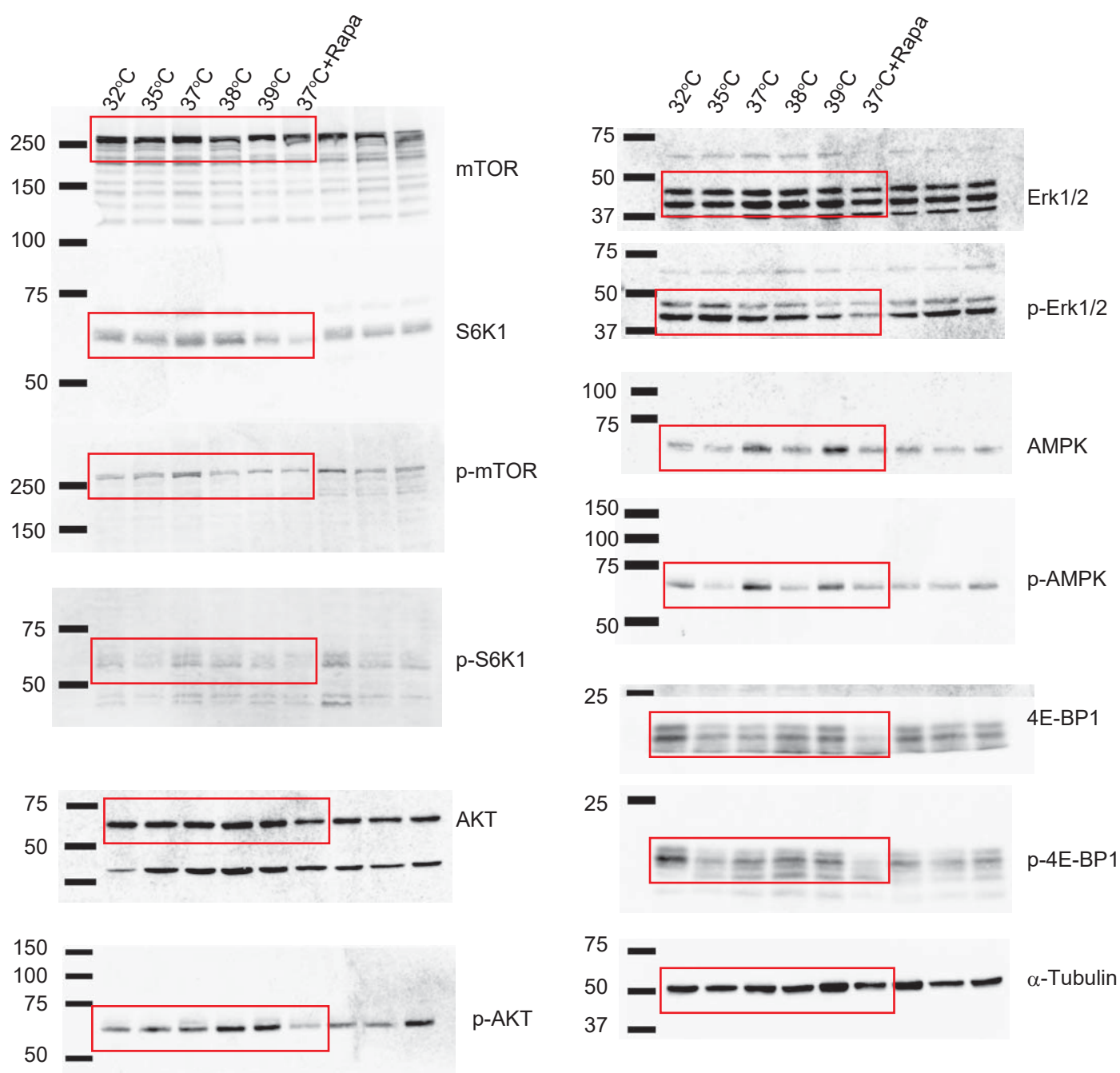

## B (1 h & 24 h)

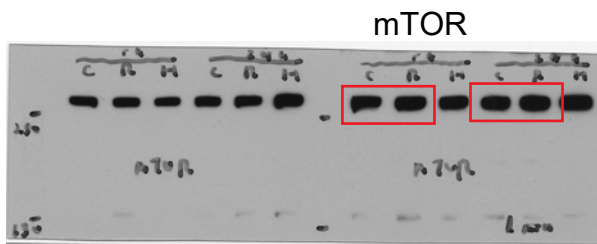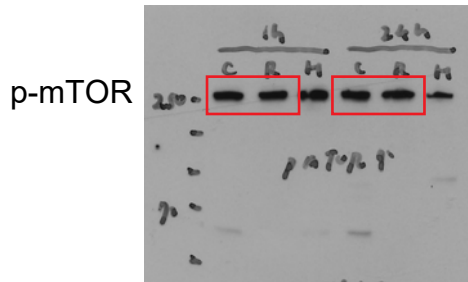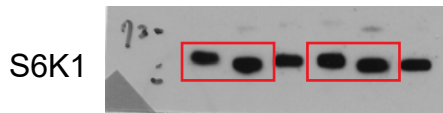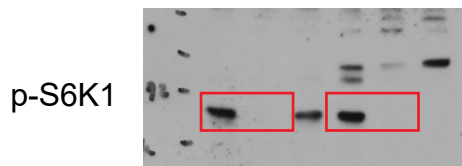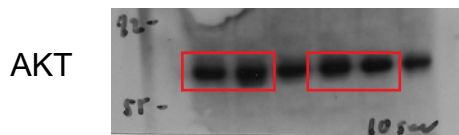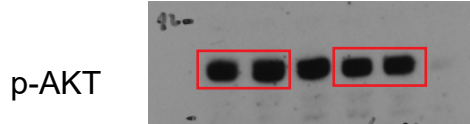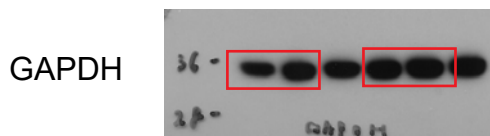

## B (7 d)

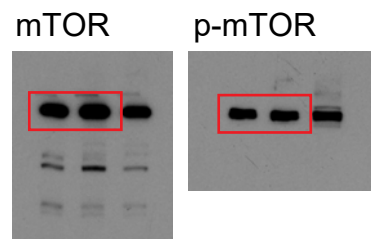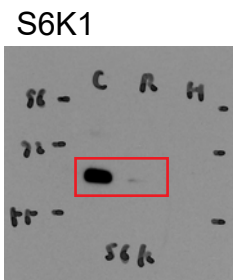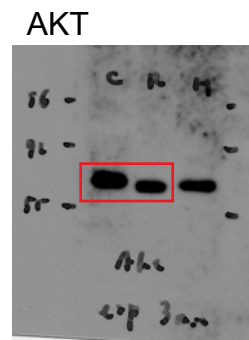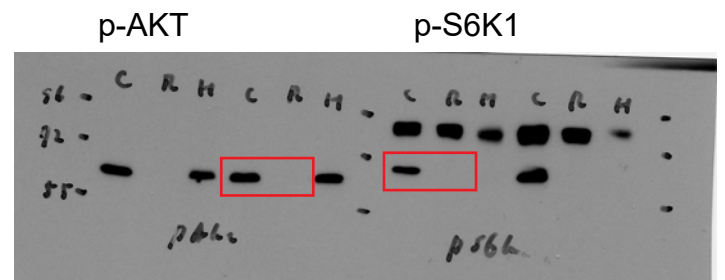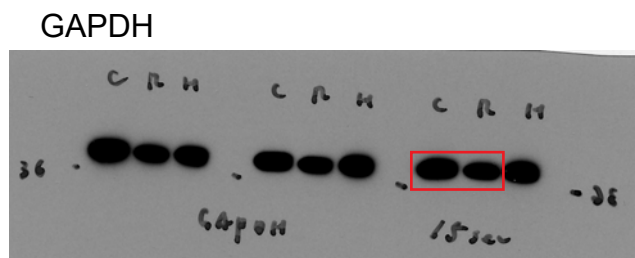

## D

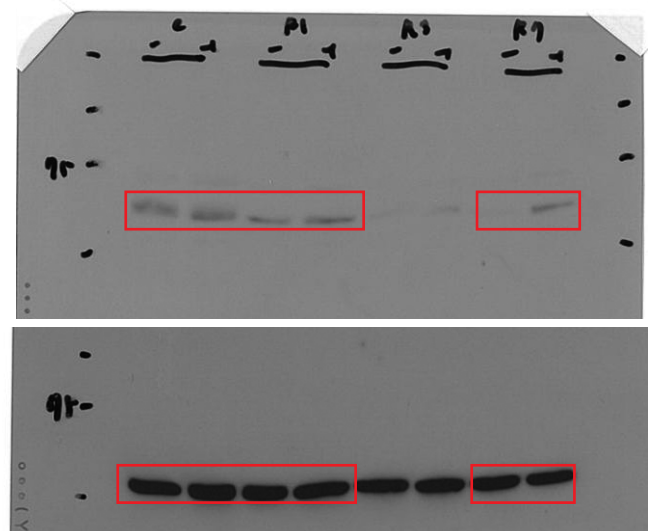

Supplement: Supplementary file 3 — Source Data for Expanded View [file EMBR-24-e55439-s001.zip › Source_Data_for_EV_Figures/EMBOR-2022-55439V3-Figure_EV4_Source_Data-sd.pdf]

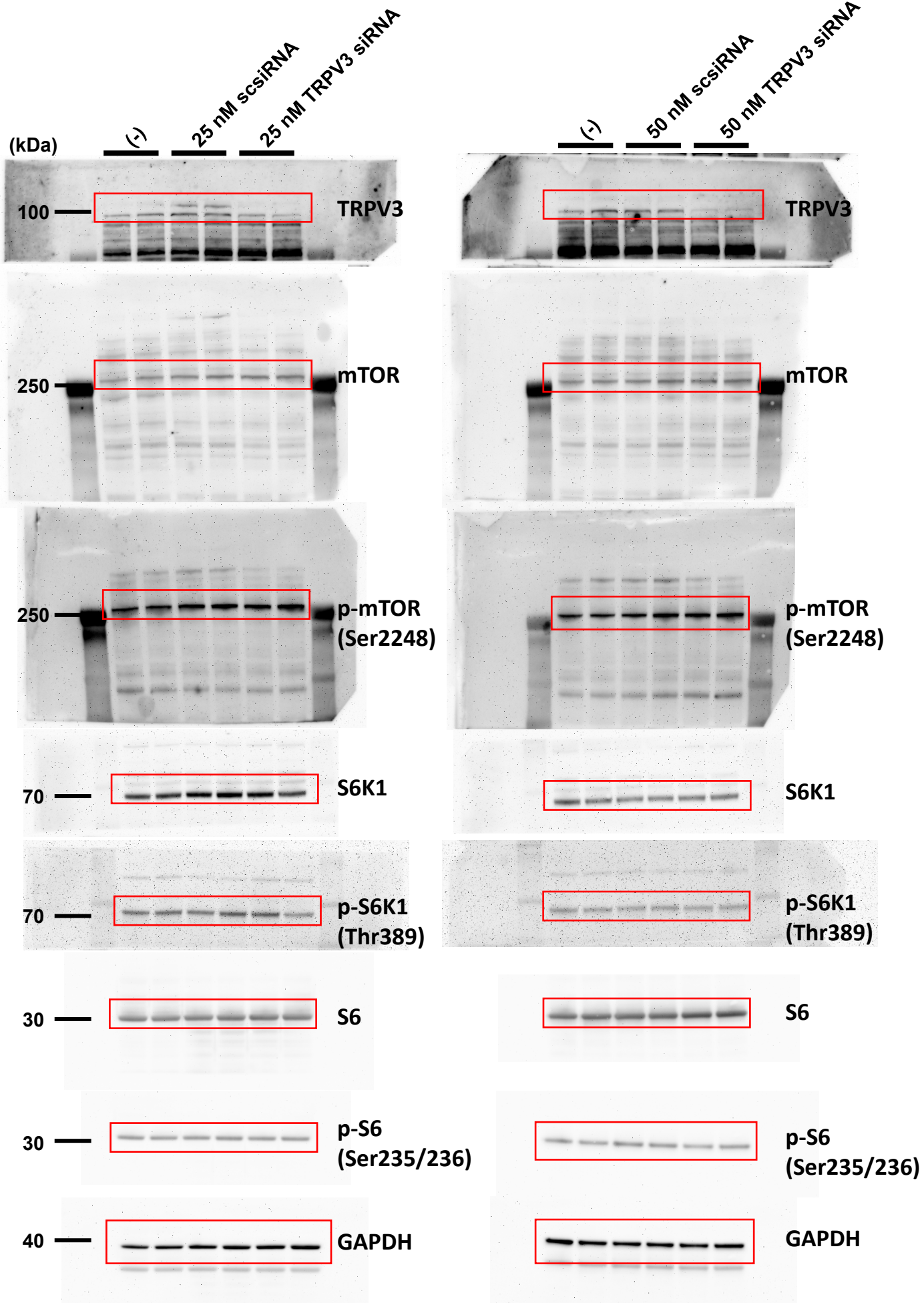

Supplement: Supplementary file 3 — Source Data for Expanded View [file EMBR-24-e55439-s001.zip › Source_Data_for_EV_Figures/EMBOR-2022-55439V3-Figure_EV3_Source_Data-sd.pdf]

F

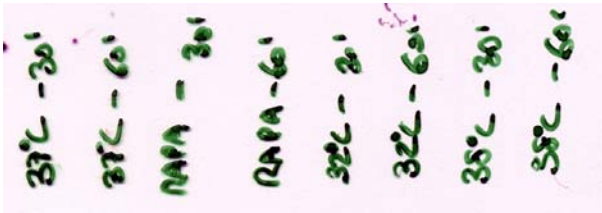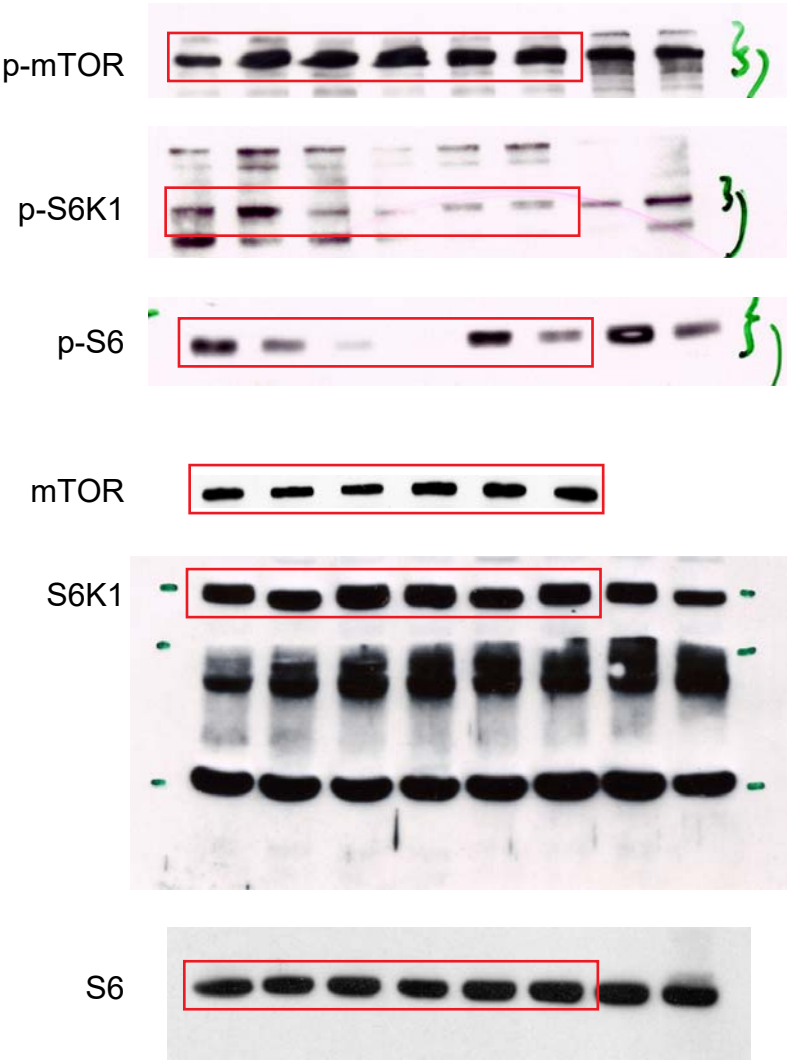

G

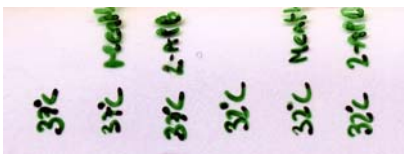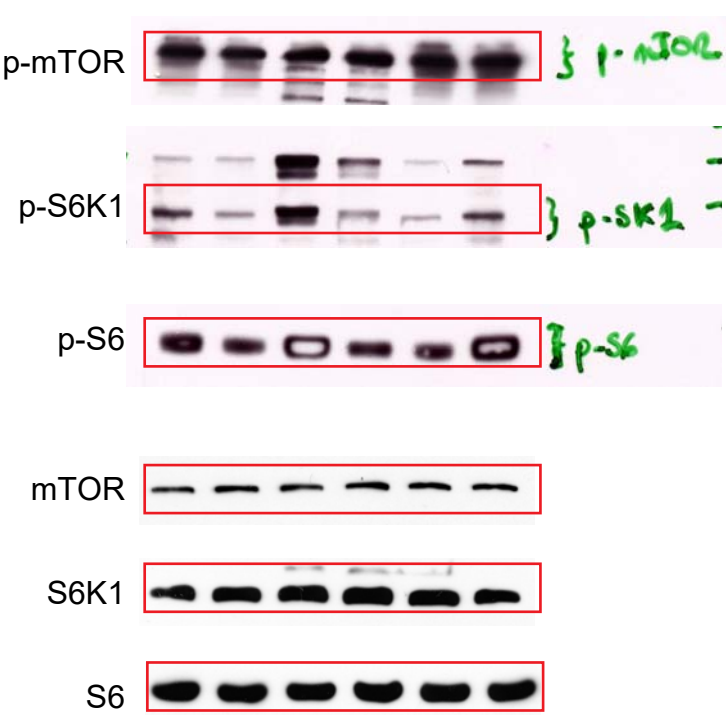

Supplement: Supplementary file 5 — Source Data for Figure 2 [file EMBR-24-e55439-s002.pdf]

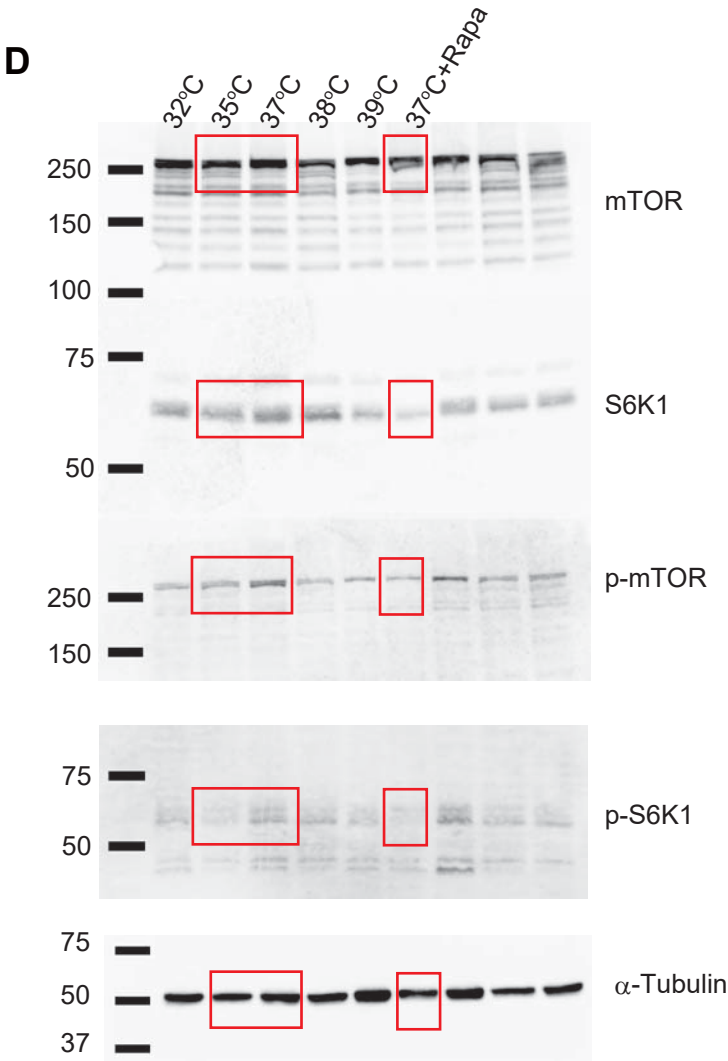

Supplement: Supplementary file 6 — Source Data for Figure 3 [file EMBR-24-e55439-s003.pdf]

**B**

mTOR

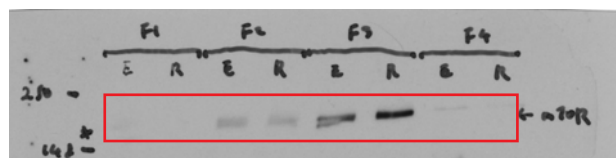

CANX & HDAC2

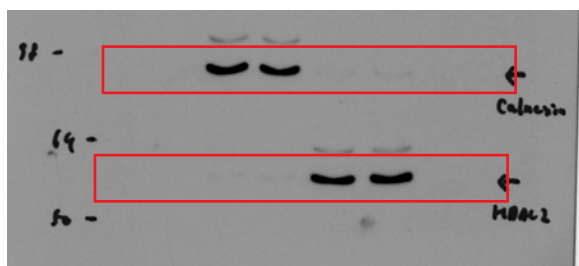

GAPDH

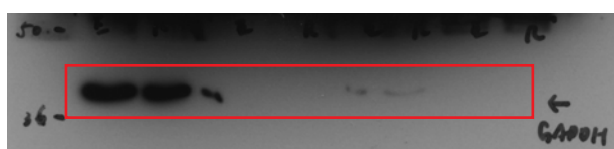

KRT5

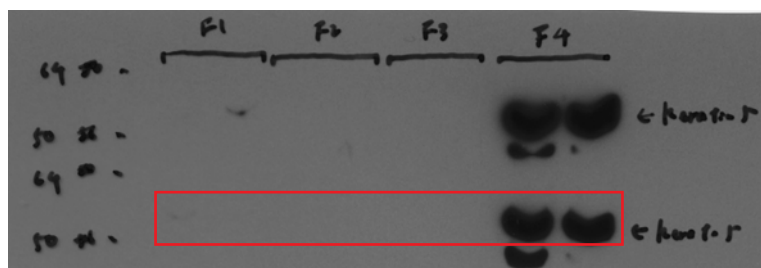

p-mTOR

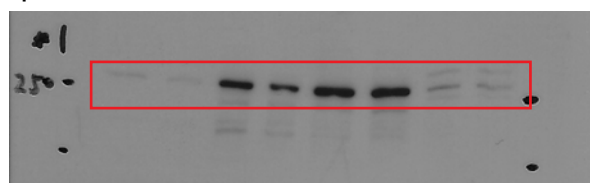

Raptor

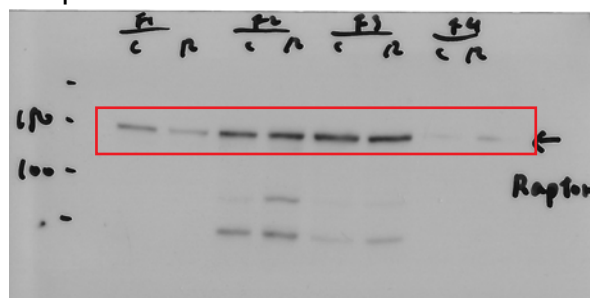

Rictor

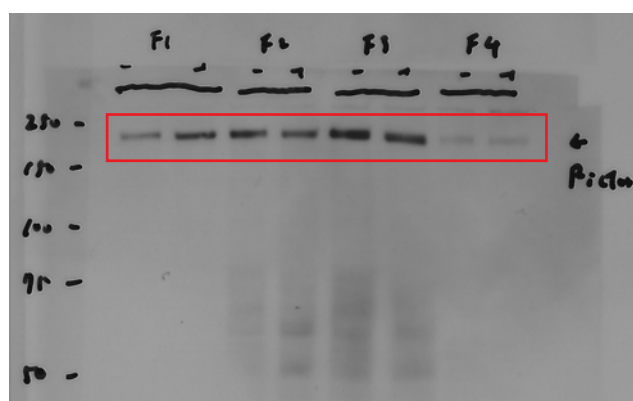

Supplement: Supplementary file 7 — Source Data for Figure 4 [file EMBR-24-e55439-s004.pdf]

B

Original data for Fig 7

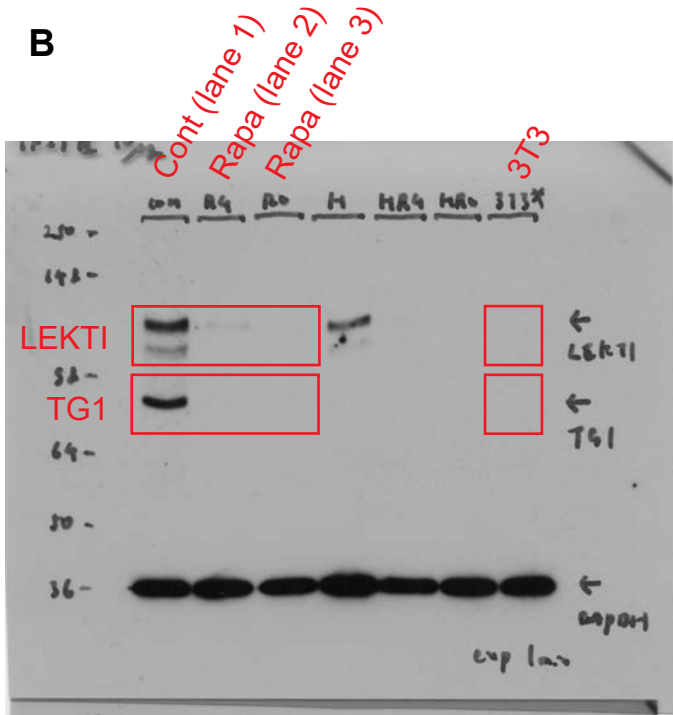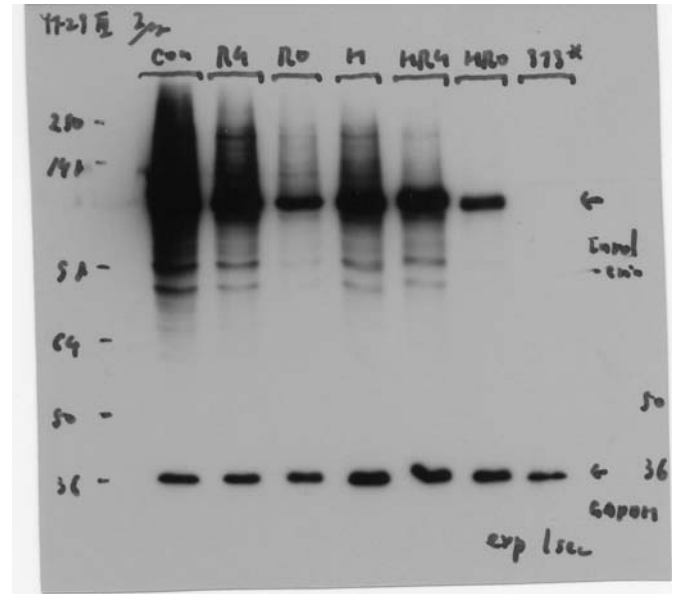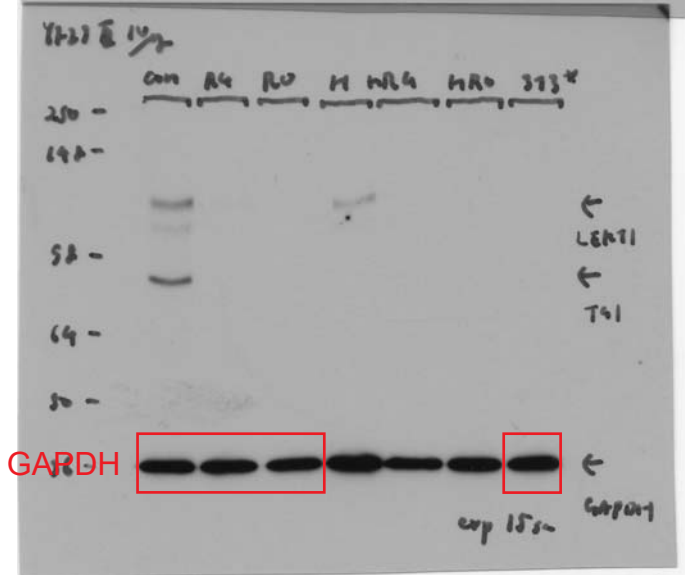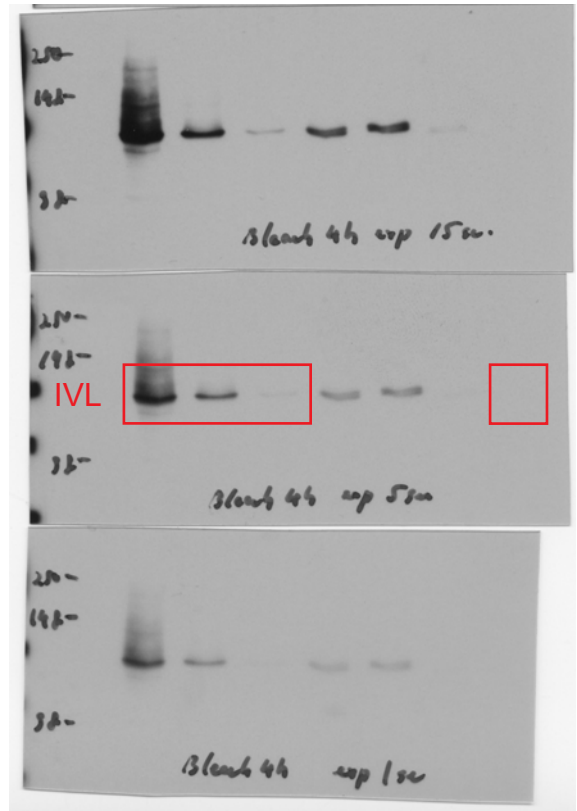

YF29 III

YF29 IV

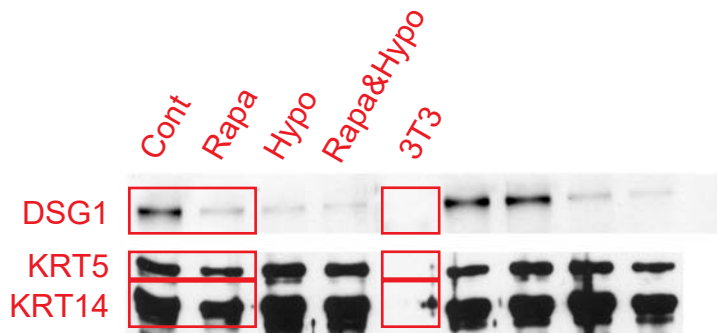

Supplement: Supplementary file 8 — Source Data for Figure 7 [file EMBR-24-e55439-s006.pdf]
