# Supplementary material for: Low temperature and mTOR inhibition favor stem cell maintenance in human keratinocyte cultures
Source: EMBO Rep. 2023 May 4;24(6):e55439. doi: 10.15252/embr.202255439 (PMC10240198; doi:10.15252/embr.202255439)
Supplement: Supplementary file 9 [file EMBR-24-e55439-s008.pdf]

EMBO reports

# Low temperature and mTOR inhibition favor stem cell maintenance in human keratinocyte cultures

Daisuke Nanba, Jun-Ichi Sakabe, Johannes Mosig, Michel Brouard, Fujio Toki, Mariko Shimokawa, Mako Kamiya, Thomas Braschler, Fahd Azzabi, Stéphanie Droz-Georget Lathion, Kai Johnsson, Keya Roy, Christoph Schmid, Jean-Baptiste Bureau, Ariane Rochat, and Yann Barrandon
**DOI: 10.15252/embr.202255439**

*Corresponding author(s): Daisuke Nanba (nanba@g.ecc.u-tokyo.ac.jp)*



## Review Timeline:

| | |
|---|---|
| Submission Date: | 18th May 22 |
| Editorial Decision: | 24th Jun 22 |
| Revision Received: | 22nd Nov 22 |
| Editorial Decision: | 17th Feb 23 |
| Revision Received: | 26th Mar 23 |
| Accepted: | 14th Apr 23 |

*Editor: Achim Breiling*


## Transaction Report:

(Note: With the exception of the correction of typographical or spelling errors that could be a source of ambiguity, letters and reports are not edited. Depending on transfer agreements, referee reports obtained elsewhere may or may not be included in this compilation. Referee reports are anonymous unless the Referee chooses to sign their reports.)

Dear Dr. Nanba,

Thank you for the transfer of your research manuscript to EMBO reports. I have now received the reports from the three referees that were asked to evaluate your study, which can be found at the end of this email.

As you will see, the referees think that these findings are of interest. However, all referees have several comments, concerns, and suggestions, indicating that a major revision of the manuscript is necessary to allow publication of the study in EMBO reports. As the reports are below, and all their points need to be addressed, I will not detail them here. Referee #1 seems rather suggest rejection, but after cross-commenting with the other referees, and also considering the interesting topic, I decided to proceed.

Referee #2 suggested during cross-commenting:
'Doing colony forming assays in virally transduced primary cells can be technically very difficult, but siRNA for TRPV3 could be perhaps used to give a short term (48-96 hour) read out of the effects of knockdown on temperature/TRPV3 agonists on MTOR signalling to meet reviewer 1's request. This would firm up the case for a direct linkage between TRPV3 and the MTOR pathway.'

Given the constructive referee comments, we would like to invite you to revise your manuscript with the understanding that all referee concerns must be addressed in the revised manuscript and in a detailed point-by-point response. Acceptance of your manuscript will depend on a positive outcome of a second round of review. It is EMBO reports policy to allow a single round of revision only and acceptance of the manuscript will therefore depend on the completeness of your responses included in the next, final version of the manuscript.

Revised manuscripts should be submitted within three months of a request for revision. Please contact me to discuss the revision (also by video chat) if you have questions or comments regarding the revision, or should you need additional time.

When submitting your revised manuscript, please also carefully review the instructions that follow below.

PLEASE NOTE THAT upon resubmission revised manuscripts are subjected to an initial quality control prior to exposition to re-review. Upon failure in the initial quality control, the manuscripts are sent back to the authors, which may lead to delays. Frequent reasons for such a failure are the lack of the data availability section (please see below) and the presence of statistics based on n=2 (the authors are then asked to present scatter plots or provide more data points).

When submitting your revised manuscript, we will require:

1) a .docx formatted version of the final manuscript text (including legends for main figures, EV figures and tables), but without the figures included. Figure legends should be compiled at the end of the manuscript text.

2) individual production quality figure files as .eps, .tif, .jpg (one file per figure), of main figures and EV figures. Please upload these as separate, individual files upon re-submission.

The Expanded View format, which will be displayed in the main HTML of the paper in a collapsible format, has replaced the Supplementary information. You can submit up to 5 images as Expanded View. Please follow the nomenclature Figure EV1, Figure EV2 etc. The figure legend for these should be included in the main manuscript document file in a section called Expanded View Figure Legends after the main Figure Legends section. Additional Supplementary material should be supplied as a single pdf file labeled Appendix. The Appendix should have page numbers and needs to include a table of content on the first page (with page numbers) and legends for all content. Please follow the nomenclature Appendix Figure Sx, Appendix Table Sx etc. throughout the text, and also label the figures and tables according to this nomenclature.

For more details, please refer to our guide to authors:
http://www.embopress.org/page/journal/14693178/authorguide#manuscriptpreparation

Please consult our guide for figure preparation:
http://wol-prod-cdn.literatumonline.com/pb-assets/embo-site/EMBOPress_Figure_Guidelines_061115-1561436025777.pdf

See also the guidelines for figure legend preparation:
https://www.embopress.org/page/journal/14693178/authorguide#figureformat

3) a complete author checklist, which you can download from our author guidelines (https://www.embopress.org/page/journal/14693178/authorguide). Please insert page numbers in the checklist to indicate where the requested information can be found in the manuscript. The completed author checklist will also be part of the RPF.

Please also follow our guidelines for the use of living organisms, and the respective reporting guidelines: http://www.embopress.org/page/journal/14693178/authorguide#livingorganisms

4) that primary datasets produced in this study (e.g. RNA-seq, ChIP-seq, structural and array data) are deposited in an appropriate public database. If no primary datasets have been deposited, please also state this in a dedicated section (e.g. 'No primary datasets have been generated and deposited'), see below.

See also: http://embor.embopress.org/authorguide#datadeposition

Please remember to provide a reviewer password if the datasets are not yet public.

The accession numbers and database should be listed in a formal "Data Availability" section (placed after Materials & Methods) that follows the model below. This is now mandatory (like the COI statement). Please note that the Data Availability Section is restricted to new primary data that are part of this study. This section is mandatory. As indicated above, if no primary datasets have been deposited, please state this in this section

# Data availability

The datasets produced in this study are available in the following databases:

- RNA-Seq data: Gene Expression Omnibus GSE46843 (https://www.ncbi.nlm.nih.gov/geo/query/acc.cgi?acc=GSE46843)
- [data type]: [name of the resource] [accession number/identifier/doi] ([URL or identifiers.org/DATABASE:ACCESSION])

*** Note - All links should resolve to a page where the data can be accessed. ***

Moreover, I have these editorial requests:

6) We strongly encourage the publication of original source data with the aim of making primary data more accessible and transparent to the reader. The source data will be published in a separate source data file online along with the accepted manuscript and will be linked to the relevant figure. If you would like to use this opportunity, please submit the source data (for example scans of entire gels or blots, data points of graphs in an excel sheet, additional images, etc.) of your key experiments together with the revised manuscript. If you want to provide source data, please include size markers for scans of entire gels, label the scans with figure and panel number, and send one PDF file per figure.

7) Our journal encourages inclusion of *data citations in the reference list* to directly cite datasets that were re-used and obtained from public databases. Data citations in the article text are distinct from normal bibliographical citations and should directly link to the database records from which the data can be accessed. In the main text, data citations are formatted as follows: "Data ref: Smith et al, 2001" or "Data ref: NCBI Sequence Read Archive PRJNA342805, 2017". In the Reference list, data citations must be labeled with "[DATASET]". A data reference must provide the database name, accession number/identifiers and a resolvable link to the landing page from which the data can be accessed at the end of the reference. Further instructions are available at: http://www.embopress.org/page/journal/14693178/authorguide#referencesformat

8) Regarding data quantification and statistics, please make sure that the number "n" for how many independent experiments were performed, their nature (biological versus technical replicates), the bars and error bars (e.g. SEM, SD) and the test used to calculate p-values is indicated in the respective figure legends (also for potential EV figures and all those in the final Appendix). Please also check that all the p-values are explained in the legend, and that these fit to those shown in the figure. Please provide statistical testing where applicable. Please avoid the phrase 'independent experiment', but clearly state if these were biological or technical replicates. See also: http://www.embopress.org/page/journal/14693178/authorguide#statisticalanalysis

9) Please also note our reference format: http://www.embopress.org/page/journal/14693178/authorguide#referencesformat

10) Please add scale bars of similar style and thickness to all the microscopic images (main and EV figures), using clearly visible black or white bars (depending on the background). Please place these in the lower right corner of the images. Please do not write on or near the bars in the image but define the size in the respective figure legend.

11) We updated our journal's competing interests policy in January 2022 and request authors to consider both actual and perceived competing interests. Please review the policy https://www.embopress.org/competing-interests and update your competing interests if necessary. Please name this section 'Disclosure and Competing Interests Statement' and put it after the Acknowledgements section.

I look forward to seeing a revised version of your manuscript when it is ready. Please let me know if you have questions or comments regarding the revision.

Please use this link to submit your revision: https://embor.msubmit.net/cgi-bin/main.plex

Yours sincerely,

Achim Breiling
Senior Editor
EMBO Reports

-------------
Referee #1:

This study attempts to describe the role of temperature in regulating stemness in keratinocytes via controlling the ThermoTRPV3 of mTOR signaling, which is proposed to lead to inhibition of differentiation genes. While the idea overall is interesting, unfortunately the supporting data is not sufficient in this shape to back-up the overall model to a level required by the rigorous publications in EMBO. Given the extent of the revisions and the gaps in the experimental approach (see point-by-point below), this study would probably be best submitted for consideration elsewhere.

• Targeting genetically TRPV3 (perhaps by siRNA) and evaluating the effect on mTOR and keratinocyte behavior would be helpful to examine direct instead of correlative effects.

• A rescue experiment in which opposing temperature vs mTOR effects can compensate each other would greatly strengthen this study to demonstrate that temperature effects are really mediated via mTOR.

• What was the rational and interpretation of treatment with RuR (Fig, 1D) and menthol? It was not explained in text.

• Figure S3 B-E - how many times were these experiments done? Quantification? Statistics?

• The S6k1 and p-S6k1 western blots look great in S3C, but not convincing in S3A. This data should be probably removed or replaced by better western blots.

• The rational of the inhibition experiments and their interpretation should be spelled out and clarified in text for the non-mTOR specialist in the section "ThermoTRP channels activation is wired to mTOR signaling"

• Experiments in Figures 1-2 and associated supplement demonstrate a decrease in mTOR signaling associated with lower temperatures. This is correlated with smaller colonies and smaller cell size, which are consistent with decrease mTOR signaling. The conclusion at the end of the paragraph that "these experiments demonstrated that a variation in temperature as small as one or two degrees Celsius impacted stem cell behavior through mTOR signaling" is not yet justified here based on data in figure 1&2. The cell behavior itself was not directly linked to mTOR by this point; the observations are just correlative up to this point in the paper.

• The association of rapamycin-induced changes with those changes induced by temperature in S4A is very striking and should be moved to main figures. Also "mRNA expression of epidermal differentiation-associated molecules (Figure 3A)". Fig 3A does not show where the epidermal differentiation genes are on this heat plot. Instead, it does show a nice association of the mRNAs changed at 32C with genes from rapamycin treated cells. This should be clearly stated in text.

• With that above said, the genomic analysis of cells cultured for 10 days in the different conditions simply reflects differences in the % of cells that are differentiated vs proliferating at that stage. Because the data is collected late, the difference in mRNA or miRNA expression are a consequence not a cause of direct effects of temperature or mTOR inhibition on the expression of these genes and miRNAs. The genomic analysis should be repeated at early time points, as soon as possible after temperature switch or after rapamycin treatment -within probably 24 hrs or so - and before the phenotype of the cell cultures (reduced colony size and cell size and decrease differentiation in the treated group) is already onset. Thus, early time points will capture the causative instead of currently the consequential changes.

• It is not clear what is the relevance of experiments in figure 4 to the story of this paper until the Discussion. This should be stated clearly at the beginning and end of the section.

• What are the H&E-stained samples in Figure 5A, bottom panel? - is that a stratification assay? this should be mentioned in text

• The telomerase assay in Figure 5A requires a positive and negative control and is not very credible in current form

• The transplantation experiments in 5E should also be done with temperature treated keratinocytes. With that said it is not clear what does the data in figure 5E really show. How were the rapamycin treated stem cells better than control? It is not clear from that one picture shown.

• The effect of temperature on keratinocyte phenotypes and in skin reconstitution assays (rather than that of rapamycin) is especially important to strongly document experimentally here, given the rapamycin effect on keratinocytes is already known since 1997. (Javier AF, Bata-Csorgo Z, Ellis CN, Kang S, Voorhees JJ, Cooper KD (1997) Rapamycin (sirolimus) inhibits proliferating cell nuclear antigen expression and blocks cell cycle in the G1 phase in human keratinocyte stem cells. J Clin Invest 99: 2094-2099)

-------------
Referee #2:

In this manuscript, the authors describe that low temperature and mTOR inhibition favour stem cell maintenance in human keratinocyte cultures. It is very interesting manuscript, where the authors have shown how human keratinocytes adjust to environmental changes through mTOR signaling. They have also reported that long term inhibition of mTORC1 reduces clonal conversion and favours stemness maintenance. These observations have important implications for translational and regenerative research. There are few points that need to be addressed to strengthen the study.

Major comments:
1. The authors mentioned on page no. 11 in last 3 lines that "These results strongly supported the notion that rapamycin exposure resulted in the maintenance of a basal and stem cell-like phenotype while repressing stratification." These conclusions are just based on the RNA seq analysis where they have shown upregulation of Krt8 and Krt18 and downregulation of Krt1, Krt10, FLG at lower temperature (Figure 3B). Here, they need to explain about how many genes are upregulated, what are the functions of those genes apart from Krt8 and Krt18. How these genes can be important for the maintenance of stem cell-like phenotype?

2. In Figure 3C and Page no. 11 the authors mentioned that "Quantitative PCR analysis confirmed that epidermal differentiation associated genes were down regulated in cells cultured at 32oC and 37oC + rapamycin and up regulated in cells cultured at 38oC". While at 38oC data shows genes upregulated including Krt5, Krt14 and also epidermal differentiation genes like Krt1, Krt10, FLG, IVL etc. compared to lower temperature. The Authors need to explain why at 38oC these cells are expressing both basal markers Krt5, Krt14 as well as differentiation markers Krt1, Krt10, FLG as in Figure 2B-D it has been shown that they form larger colonies at higher temperature.

Minor comments:
1. Figure 1E, Control image for Fluo-4 is not good quality if compare with the Figure1D Control image at 37oC, need to improve the quality of the image to conclude strong calcium influx after treating with menthol.
2. Figure 1,2 and 3, need to mention about statistical test used
3. Figure 2E, western blot for p-S6K1 quality need to be improved.

-------------
Referee #3:

This work examines the relationship between temperature responsive signaling and the behavior of human epidermal stem cells in culture. This is of relevance as the skin is directly exposed to extremes of temperature and subject to greater fluctuation in temperature than internal organs. Understanding how such environmental changes may alter proliferation is interesting, and may have implications for physiology and for other stem cell systems.

The authors begin by establishing that subcutaneous temperatures in rodents fluctuate by 2-3oC between sleep and activity. This reviewer is not qualified to assess the methods used, but the results form a strong motivation for the work presented. The observation that media change and observing cells in culture causes a prolonged lowering of temperature of up to 3 hours are striking and are a potential confounding factor in culture experiments in general.

Keratinocytes are shown to express the temperature responsive channel TRPV3. Improvised approaches with a PCR machine and a custom-made device are used to show changes in calcium with temperature which are blocked by a TRPV inhibitor. Time points should be given in Figure 1D. Western blotting and inhibitors are used to argue that whilst Mtor phosphorylation is unchanged, the levels of S6K1 vary with the activity of TRPV3, at either 37 or 32 C. This argues for regulation beyond the decrease in kinase activity that results from the 5C drop in temperature.

Clonal growth assays are then performed, over the range 35-38C. As expected colonies are larger at higher temperatures. Oddly different temperatures are used for blotting studies. Why this is done needs to be clarified, as it is impossible to correlate the changes in cell behavior with signaling. The paper would be stronger with the same temperature range (down to 32C) used for the clonal assays as the other experiments. In Fig 2E, is the reduction in kinase activity simply due to the decrease in temperature?

The RNAseq in Figure 3 is interesting, showing a disruption to the normal differentiation program at low temperatures or with Rapamycin treatment. The analysis of this data is superficial, for example were there any transcriptional hallmarks of Mtor signaling or other pathways? It would be helpful to validate the differentiation phenotype at the protein level. It would be worth discussing literature such as PMID: 27194247 that report that rapalogs block differentiation.

Figure 4 shows MTOR translocates to the nucleus, at 36C and with rapamycin treatment. The data look robust but its functional significance is unclear, so this work seems to add little to the paper.

Figure 5 describes the effects of Rapamycin on long term culture. This is a missed opportunity, as it would be interesting to know what long term culture at 32 or 35C does to cell behavior.

The discussion is quite long and could be better focused.

**Point-by-point responses to the reviewers**

**Referee #1:**

***This study attempts to describe the role of temperature in regulating stemness in keratinocytes via controlling the ThermoTRPV3 of mTOR signaling, which is proposed to lead to inhibition of differentiation genes. While the idea overall is interesting, unfortunately the supporting data is not sufficient in this shape to back-up the overall model to a level required by the rigorous publications in EMBO. Given the extent of the revisions and the gaps in the experimental approach (see point-by-point below), this study would probably be best submitted for consideration elsewhere.***

**We appreciate your valuable comments and suggestions. We have performed additional experiments and revised the manuscript, as suggested by the reviewers.**

• ***Targeting genetically TRPV3 (perhaps by siRNA) and evaluating the effect on mTOR and keratinocyte behavior would be helpful to examine direct instead of correlative effects.***

Based on your suggestion, we knocked down TRPV3 gene expression in human keratinocytes with siRNA (**new Fig EV3**). However, the phosphorylation levels of S6K and S6 did not change, even though TRPV3 expression was significantly decreased by siRNA transfection. This result strongly suggests that TRPV3 activity is not the main player in the temperature-dependent regulation of mTORC1 signaling. Together with the experiments with temperature shift (37 °C to 32 °C) and chemicals (RuR, menthol, and 2-APB) (**new Fig 2F and G**), we concluded that the temperature drop sensed through several types of thermoTRP channels inhibited mTORC1 signaling, similarly to rapamycin treatment. On page 10, lines 22-31, we have added the following sentences to indicate that TRPV3 activity dose not mainly contribute to the temperature-dependent regulation of mTORC1 signaling.

*"Next, we examined whether the activity of TRPV3 is directly involved in mTORC1 signaling as TRPV3 is activated at 33–39 °C (Venkatachalam & Montell, 2007). Human keratinocytes were treated with siRNA targeting TRPV3 and cultured at 37°C, and the mTORC1 signaling activity was examined by analyzing the phosphorylation levels of S6K1 and S6. Western blotting analysis revealed no changes in the phosphorylation levels of these proteins, although the expression of TRPV3 was significantly decreased (**Fig EV3**). This result strongly suggests that TRPV3 activity does not mainly contribute to the temperature-dependent regulation of mTORC1 signaling and that the temperature drop cooperatively sensed through several types of thermoTRP channels inhibits mTORC1 signaling similar to rapamycin treatment."*

We have changed the structure of the manuscript accordingly. In the previous version of the manuscript, the readers could have misunderstood that temperature regulates keratinocyte stem cells through the TRPV3-mTORC1 signaling axis. In the revised manuscript, we started the Results section with the effects of temperature on human keratinocyte stem cells (**new Fig 1**) and described that TRPV3 as one of the activated thermoTRP channels expressed in human keratinocytes at approximately 37 °C, the temperature of normal cell cultures, as follows (page 8, lines 25-30).

*"All thermoTRP channels were transcribed to a certain extent by cultured human keratinocytes (YF29 and OR-CA) when analyzed by quantitative PCR (Fig 2A). Additionally, we confirmed the expression of TRPV3 in cultured human keratinocytes by western blotting and immunocytochemistry (Fig 2B) TRPV3 is activated at approximately 37 °C, the temperature of standard cell cultures (Moqrich et al, 2005; Peier et al, 2002; Venkatachalam & Montell, 2007)."*

***• A rescue experiment in which opposing temperature vs mTOR effects can compensate each other would greatly strengthen this study to demonstrate that temperature effects are really mediated via mTOR.***

Thank you for your valuable comments. Based on your suggestion, we performed a colony-forming assay of human keratinocytes under 35 °C, 37 °C, and 37 °C + rapamycin conditions (**new Fig 3A**). We also evaluated the number and size of the cells under these experimental conditions (**new Fig 3B and C**). Culture at 37 °C increased keratinocyte colony size and the number and size of keratinocytes compared to the culture at 35 °C. However, rapamycin treatment decreased keratinocyte colony size and the number and size of keratinocytes, even when cells were cultured at 37 °C. This result suggests that the effects of high temperature (37 °C) on human keratinocyte behavior can be compensated by inhibiting mTORC1 signaling by rapamycin.

We also demonstrated that the reduction of mTORC1 kinase activity was observed with a short-term (30 and 60 min) temperature drop (37 °C to 32 °C), which was compensated with 2-APB, a TRPVs agonist (**new Fig 2G**). Therefore, we concluded that temperature-regulated mTORC1 activity through thermo-sensitive TRP channels. We added the following sentence to emphasize the importance of temperature in mTORC1 kinase activity on page 10, lines 16-20.

*"The finding that treatment with 2-APB increased mTORC1 kinase activity even at 32 °C indicates that lower temperatures do not reduce the activity of chemical reactions but regulate mTORC1 activity through thermoTRP channels. Therefore, these experiments support our hypothesis that thermoTRP channels are connected to the mTOR signaling pathway through calcium influx."*

***• What was the rational and interpretation of treatment with RuR (Fig, 1D) and menthol? It was not explained in text.***

Thank you for your comments. Based on your suggestion, we have described the interpretation of the results of RuR (**new Fig 2D**) and menthol (**new Fig 2E**) treatment on calcium influx in human keratinocytes in the text (page 9, lines 20-22) as follows:

*"These experiments confirmed that cultured human keratinocytes express functional thermo-sensitive TRP channels and that modulation of the activity of these channels causes changes in the intracellular calcium concentration."*

***• Figure S3 B-E - how many times were these experiments done? Quantification? Statistics?***

Based on your suggestion, we added the statistical information in the Figure legends.

***• The S6k1 and p-S6k1 western blots look great in S3C, but not convincing in S3A. This data should be probably removed or replaced by better western blots.***

Based on your suggestion, we removed Figure S3A.

***• The rational of the inhibition experiments and their interpretation should be spelled out and clarified in text for the non-mTOR specialist in the section "ThermoTRP channels activation is wired to mTOR signaling"***

Thank you for your valuable comments. We have added detailed information about S6 kinase 1 (S6K1) and ribosomal protein S6 (S6) as downstream targets of mTORC1, and menthol and 2-APB as thermoTRP channel agonists in the text (page 10, lines 3-14).

***• Experiments in Figures 1-2 and associated supplement demonstrate a decrease in mTOR signaling associated with lower temperatures. This is correlated with smaller colonies and smaller cell size, which are consistent with decrease mTOR signaling. The conclusion at the end of the paragraph that "these experiments demonstrated that a variation in temperature as small as one or two degrees Celsius impacted stem cell behavior through mTOR signaling" is not yet justified here based on data in figure 1&2. The cell behavior itself was not directly linked to mTOR by this point; the observations are just correlative up to this point in the paper.***

Thank you for your important comment. As you pointed out, **Fig 1 and 2** in the first version were insufficient to justify the conclusion that "these experiments demonstrated that a variation in temperature as small as one or two degrees Celsius impacted stem cell behavior through mTOR signaling." Based on your suggestion, we have added **new Fig 3** as described above. As the experiments in the **new Fig 1-3** can justify this conclusion, we have maintained this sentence at the end of the paragraph that explains the results of **new Fig 3** on page 11, lines 18-20.

***• The association of rapamycin-induced changes with those changes induced by temperature in S4A is very striking and should be moved to main figures. Also "mRNA expression of epidermal differentiation-associated molecules (Figure 3A)". Fig 3A does not show where the epidermal differentiation genes are on this heat plot. Instead, it does show a nice association of the mRNAs changed at 32C with genes from rapamycin treated cells. This should be clearly stated in text.***

Thank you for your valuable comments. Based on your suggestion, we have moved the results of miRNA expression to the main figure (**new Fig 6**). As you pointed out, **Fig 3A** (**new Fig 5A**) did not show the expression of epidermal differentiation-associated molecules. Based on your suggestion, we have added the following sentence to indicate a strong association between global mRNA expression at 32 °C and rapamycin treatment on page 12, line 34, and page 13, lines 1-2.

*"The cluster analysis of gene expression revealed that one-degree Celsius difference in temperature impacted global mRNA expression and that the addition of rapamycin to cells cultured at 37 °C had a similar effect to that at 32 °C (**Fig 5A**)."*

***• With that above said, the genomic analysis of cells cultured for 10 days in the different conditions simply reflects differences in the % of cells that are differentiated vs proliferating at that stage. Because the data is collected late, the difference in mRNA or miRNA expression are a consequence not a cause of direct effects of temperature or mTOR inhibition on the expression of these genes and miRNAs. The genomic analysis should be repeated at early time points, as soon as possible after temperature switch or after rapamycin treatment -within probably 24 hrs or so - and before the phenotype of the cell cultures (reduced colony size and cell size and decrease differentiation in the treated group) is already onset. Thus, early time points will capture the causative instead of currently the consequential changes.***

Thank you for your helpful comments. Based on your suggestion, we performed qPCR experiments to investigate the changes in the expression of genes regulated by long-term temperature switch and rapamycin treatment for 24 h, as shown below. [Figures for referees not shown.]

Surprisingly, the expression of most of the genes examined, except *KRT1* and *KRT10,* did not show significant changes within 24 h of treatment. These data suggest that the thermoTRP channels-mTORC1 signaling axis does not directly regulate epidermal differentiation-associated genes and that master regulator genes of epidermal differentiation might be involved in these processes. This finding raises the important question of how temperature and mTORC1 inhibition regulate the expression of epidermal differentiation-associated genes. Further investigations are required to unveil the detailed mechanisms of gene regulation, and we would like to use these data in the next project.

***• It is not clear what is the relevance of experiments in figure 4 to the story of this paper until the Discussion. This should be stated clearly at the beginning and end of the section.***

Thank you for your comments. We could not reveal the functional significance of nuclear mTOR in keratinocytes, but the finding strongly suggests that mTOR complexes directly regulate transcription, as discussed recently (Giguére, FEBS J, 2018; Torres and Holz, BBA Mol Cell Res 2021). To emphasize the importance of the nuclear localization of mTOR in response to temperature shift and rapamycin treatment, we have added the following sentence to explain why we analyzed the localization of mTOR and associated molecules on page 11, lines 23-26. We also described the importance of the nuclear localization of mTOR and its nuclear localization in keratinocytes before presenting the RNA-seq results to improve the flow of the manuscript.

*"Recent studies have revealed that mTOR complexes translocate to the nucleus and directly regulate transcription (Giguère, 2018; Torres & Holz, 2021). Next, we examined the effects of temperature and rapamycin on the translocation of the mTOR complexes."*

***• What are the H&E-stained samples in Figure 5A, bottom panel? - is that a stratification assay? this should be mentioned in text***

Human keratinocyte stem cells generate a stratified epithelial sheet similar to the epidermis when the culture becomes confluent. H&E-stained samples in **new Fig 7A** are confluent cultures of normal and rapamycin-treated keratinocytes. To explain the results of confluent cultured sheets more precisely, we have added the following sentence on page 15, lines 6-11.

*"Human keratinocyte stem cells generate a stratified epithelial sheet similar to the epidermis when the culture becomes confluent, and can be used for autologous transplantation of cultured epidermal sheets when detached from the culture vessel with dispase (Green et al, 1979). We also found that confluent rapamycin-treated keratinocytes formed a thinner epithelium than untreated cells, suggesting an effect on differentiation (Fig 7A)."*

***• The telomerase assay in Figure 5A requires a positive and negative control and is not very credible in current form***

Thank you for your comments. Progressive telomere shortening in serial cultivation of human keratinocytes under standard culture conditions (passages VIII and XII) was used as a control experiment.

Telomere shortening in keratinocytes treated with rapamycin (**new Fig 7D**) also explains why the reduction in colony-forming efficiency and the ratio of growing colonies rapidly decreased after the removal of rapamycin from the culture (**new Fig 7C**). These two experiments indicated that the reduction in clonal conversion with rapamycin was not due to the transformation or immortalization of human keratinocyte stem cells. This is important for medical applications. Therefore, we have retained the result of the telomere assay in **new Fig 7**.

***• The transplantation experiments in 5E should also be done with temperature treated keratinocytes. With that said it is not clear what does the data in figure 5E really show.***

***How were the rapamycin treated stem cells better than control? It is not clear from that one picture shown.***

Thank you for your comment. It takes a long time to prepare epidermal sheets at low temperatures and transplant them into immunodeficient mice. Based on your suggestion, we performed short-term serial cultures of human keratinocytes at 35 °C. We confirmed that colony-forming efficiency is maintained at this temperature even in later passages, similar to rapamycin treatment, as shown below. [Figures for referees not shown.]

However, the aim of this study was to identify the mechanisms involved in stem cell maintenance and to define culture conditions to maintain stemness and improve stem cell-based regenerative medicine. To apply our findings to regenerative medicine, treatment of rapamycin to stem cell cultures is easier than developing large-scale cell culture devices with precise temperature control since rapamycin has been used in clinics worldwide for a long time as an immunosuppressive drug. To emphasize the importance of rapamycin treatment in human keratinocyte cultures and regenerative medicine, we added the following sentence to explain why we intensively analyzed the effects of rapamycin on human keratinocyte stem cell maintenance ex vivo on page 14, lines 29-34, and page 15, line 1.

*"In this study, we found that lower temperatures and mTORC1 inhibition maintained human keratinocyte stem cells ex vivo. To apply our findings to regenerative medicine, we further assessed the effects of decreased mTORC1 signaling with rapamycin on human keratinocyte stem cell behavior, as rapamycin is already used in clinics worldwide as an immunosuppressive drug (MacDonald, 2001), and the conventional cell culture systems present a difficulty for precise temperature control."*

In transplantation experiments, we demonstrated that rapamycin-treated human keratinocyte stem cells could generate functional epidermal sheets after transplantation as effectively as keratinocyte stem cells cultured under normal conditions. This point is crucial for applying our findings to regenerative medicine. To emphasize this point, we have added the following sentence on page 15, lines 33-34, and page 16, llines1-2.

*"Long-term exposure of keratinocyte stem cells to rapamycin did not affect capacity of the cells to generate an epidermis (Fig 7E), indicating that rapamycin-treated keratinocyte stem cells can also be used for autologous transplantation of cultured epidermal sheets."*

***• The effect of temperature on keratinocyte phenotypes and in skin reconstitution assays (rather than that of rapamycin) is especially important to strongly document experimentally here, given the rapamycin effect on keratinocytes is already known since 1997. (Javier AF, Bata-Csorgo Z, Ellis CN, Kang S, Voorhees JJ, Cooper KD (1997) Rapamycin (sirolimus) inhibits proliferating cell nuclear antigen expression and blocks cell cycle in the G1 phase in human keratinocyte stem cells. J Clin Invest 99: 2094-2099)***

Thank you for your helpful comments. As you pointed out, the effects of rapamycin on human keratinocyte stem cells have already been reported by Javier et al, J Clin Invest 1997, and we cited this article as a previous report. Here, we demonstrated that rapamycin delays the clonal conversion of human keratinocyte stem cells and can be applied to regenerative medicine. Our study has originally expanded the previous study (Janvier et al, 1997) into medical applications.

**Thank you again for your valuable comments and constructive feedback.**

**Referee #2:**

***In this manuscript, the authors describe that low temperature and mTOR inhibition favour stem cell maintenance in human keratinocyte cultures. It is very interesting manuscript, where the authors have shown how human keratinocytes adjust to environmental changes through mTOR signaling. They have also reported that long term inhibition of mTORC1 reduces clonal conversion and favours stemness maintenance. These observations have important implications for translational and regenerative research. There are few points that need to be addressed to strengthen the study.***

**We appreciate your positive comments and suggestions. We have performed additional experiments and revised the manuscript, as suggested by the reviewers.**

***Major comments:***
***1. The authors mentioned on page no. 11 in last 3 lines that "These results strongly supported the notion that rapamycin exposure resulted in the maintenance of a basal and stem cell-like phenotype while repressing stratification." These conclusions are just based on the RNA seq analysis where they have shown upregulation of Krt8 and Krt18 and downregulation of Krt1, Krt10, FLG at lower temperature (Figure 3B). Here, they need to explain about how many genes are upregulated, what are the functions of those genes apart from Krt8 and Krt18. How these genes can be important for the maintenance of stem cell-like phenotype?***

Based on your suggestion, we performed further gene expression analyses to clarify how many genes are upregulated or downregulated in both low-temperature and rapamycin-treated conditions. As shown in **new Fig EV5A and B**, rapamycin and the 32 ºC environment changed gene expression of 668 and 1,108 genes, respectively, compared with a control culture (37 ºC). Among them, 397 genes were upregulated (**Fig EV5A**). Conversely, 1,646 and 1,407 genes were downregulated by rapamycin treatment and culture at 32 ºC, respectively, and 796 genes were downregulated under both conditions(**Fig EV5B**). Moreover, we performed gene ontology (GO) analysis of genes upregulated or downregulated by both rapamycin treatment and culture at 32 ºC compared with the control culture (37 ºC) in **new Fig EV5C and D**. Although GO analysis did not indicate any particular GO terms related to stem cell maintenance among these upregulated genes (**Fig EV5C**), both rapamycin treatment and 32 ºC culture downregulated genes related to metabolic processes and protein translation (**Fig EV5D**), which are known to be regulated by mTORC1 signaling. GO analysis also confirmed that genes associated with “epidermal development” and “cornification” were downregulated by rapamycin treatment and culture at 32 ºC (**Fig EV5D**).

***2. In Figure 3C and Page no. 11 the authors mentioned that "Quantitative PCR analysis confirmed that epidermal differentiation associated genes were down regulated in cells cultured at 32oC and 37oC + rapamycin and up regulated in cells cultured at 38oC". While at 38oC data shows genes upregulated including Krt5, Krt14 and also epidermal differentiation genes like Krt1, Krt10, FLG, IVL etc. compared to lower temperature. The Authors need to explain why at 38oC these cells are expressing both basal markers Krt5, Krt14 as well as differentiation markers Krt1, Krt10, FLG as in Figure 2B-D it has been shown that they form larger colonies at higher temperature.***

Thank you for your comment. Human keratinocyte cultures contain keratinocytes with basal and stratified phenotypes expressing both basal and differentiation markers (Barrandon and Green, Proc. Natl Acad Sci USA, 1987; Nanba et al, EMBO Mol Med 2013). These cells are post-mitotic basal keratinocytes that are spontaneously generated under cell culture conditions (Roshan et al, Nat Cell Biol 2016; Nanba et al, J Cell Biol 2021). Therefore, the culture at 38 ºC significantly increased the number of post-mitotic basal keratinocytes.

Based on your suggestion, we have added the following sentence to explain why keratinocytes expressed both basal and differentiation genes at 38 ºC on page 13, line 8-18.

*"Quantitative PCR analysis confirmed that epidermal differentiation-associated genes, including KRT1, KRT10, DSG1, FLG, TGM1, IVL, and SPINK5, were downregulated in cells cultured at 32 ºC and 37 ºC + rapamycin and upregulated in cells cultured at 38 ºC (Fig 5C). Human keratinocyte cultures contain basal and stratified keratinocytes and also include keratinocytes expressing both basal and differentiation markers (Barrandon & Green, 1987; Nanba et al, 2013). This type of keratinocytes exhibits a growth-arrested phenotype and are spontaneously generated under cell culture conditions (Nanba et al, 2021; Roshan et al, 2016). Expression of both basal cell markers, including KRT5 and KRT14, and epidermal differentiation genes in keratinocytes cultured at 38 ºC indicates that higher temperatures significantly increased post-mitotic basal keratinocytes."*

***Minor comments:***
***1. Figure 1E, Control image for Fluo-4 is not good quality if compare with the Figure1D Control image at 37oC, need to improve the quality of the image to conclude strong calcium influx after treating with menthol.***

Based on your suggestion, we have improved the quality of the images (**new Fig 2D and E**).

***2. Figure 1,2 and 3, need to mention about statistical test used.***

Based on your suggestion, we added the statistical information in Figure legends.

***3. Figure 2E, western blot for p-S6K1 quality need to be improved.***

Based on your suggestion, we improved the quality of the western blot image for p-S6K in **new Fig 3D** and removed the blot for the 32 ºC experiment from the figure, because this figure does not contain the effects of 32 ºC culture on keratinocyte behaviors.

**Thank you again for your positive comments and constructive feedback.**

**Referee #3:**

***This work examines the relationship between temperature responsive signaling and the behavior of human epidermal stem cells in culture. This is of relevance as the skin is directly exposed to extremes of temperature and subject to greater fluctuation in temperature than internal organs. Understanding how such environmental changes may alter proliferation is interesting, and may have implications for physiology and for other stem cell systems.***

***The authors begin by establishing that subcutaneous temperatures in rodents fluctuate by 2-3oC between sleep and activity. This reviewer is not qualified to assess the methods used, but the results form a strong motivation for the work presented. The observation that media change and observing cells in culture causes a prolonged lowering of temperature of up to 3 hours are striking and are a potential confounding factor in culture experiments in general.***

**We appreciate your positive comments and suggestions. We have performed additional experiments and revised the manuscript, as suggested by the reviewers.**

***Keratinocytes are shown to express the temperature responsive channel TRPV3. Improvised approaches with a PCR machine and a custom-made device are used to show changes in calcium with temperature which are blocked by a TRPV inhibitor. Time points should be given in Figure 1D. Western blotting and inhibitors are used to argue that whilst Mtor phosphorylation is unchanged, the levels of S6K1 vary with the activity of TRPV3, at either 37 or 32 C. This argues for regulation beyond the decrease in kinase activity that results from the 5C drop in temperature.***

Thank you for your helpful comments. The temperature was switched between 32 ºC and 37 ºC every 30 s using our custom-made device (**Fig EV2**). Based on your suggestion, we have added this information to **new Fig 2D** and the figure legend for the **new Fig 2D**.

As you pointed out, experiments with temperature shift (37 ºC to 32 ºC) and chemicals (RuR, menthol, and 2-APB) could not explain whether TRPV3 directly regulates mTORC1 kinase activity. Therefore, we performed knockdown of TRPV3 gene expression in human keratinocytes with siRNA (**new Fig EV3**). However, the phosphorylation levels of S6K and S6 did not change, even though TRPV3 expression was significantly decreased by siRNA transfection. This result strongly suggests that TRPV3 activity is not the main player in the temperature-dependent regulation of mTORC1 signaling. Therefore, we conclude that the temperature drop sensed through several types of thermoTRP channels inhibits mTORC1 signaling, similarly to rapamycin treatment. we have added the following sentences to indicate that TRPV3 activity dose not mainly contribute to the temperature-dependent regulation of mTORC1 signaling.

*"Next, we examined whether the activity of TRPV3 is directly involved in mTORC1 signaling as TRPV3 is activated at 33–39 °C (Venkatachalam & Montell, 2007). Human keratinocytes were treated with siRNA targeting TRPV3 and cultured at 37°C, and the mTORC1 signaling activity was examined by analyzing the phosphorylation levels of S6K1 and S6. Western blotting analysis revealed no changes in the phosphorylation levels of these proteins,*

*although the expression of TRPV3 was significantly decreased (**Fig EV3**). This result strongly suggests that TRPV3 activity does not mainly contribute to the temperature-dependent regulation of mTORC1 signaling and that the temperature drop cooperatively sensed through several types of thermoTRP channels inhibits mTORC1 signaling similar to rapamycin treatment."*

We have changed the structure of the manuscript accordingly. In the previous version of the manuscript, the readers could have misunderstood that temperature regulates keratinocyte stem cells through the TRPV3-mTORC1 signaling axis. In the revised manuscript, we started the Results section with the effects of temperature on human keratinocyte stem cells (**new Fig 1**) and described that TRPV3 as one of the activated thermoTRP channels expressed in human keratinocytes at approximately 37 ºC, the temperature of normal cell cultures, as follows (page 8, lines 25-30).

*"All thermoTRP channels were transcribed to a certain extent by cultured human keratinocytes (YF29 and OR-CA) when analyzed by quantitative PCR (Fig 2A). Additionally, we confirmed the expression of TRPV3 in cultured human keratinocytes by western blotting and immunocytochemistry (Fig 2B) TRPV3 is activated at approximately 37 °C, the temperature of standard cell cultures (Moqrich et al., 2005; Peier et al., 2002; Venkatachalam & Montell, 2007)."*

***Clonal growth assays are then performed, over the range 35-38C. As expected colonies are larger at higher temperatures. Oddly different temperatures are used for blotting studies. Why this is done needs to be clarified, as it is impossible to correlate the changes in cell behavior with signaling. The paper would be stronger with the same temperature range (down to 32C) used for the clonal assays as the other experiments. In Fig 2E, is the reduction in kinase activity simply due to the decrease in temperature?***

Thank you for your comments. As you mentioned, we cultured human keratinocytes at 32 ºC in short time (single-cell imaging) and bulk culture (RNA-seq, qPCR, Western blot) experiments. However, colony formation assay at 32 ºC was difficult because colonies could not be visualized with rhodamine B staining. Therefore, we performed colony formation assays at 35 ºC, 36 ºC, 37 ºC, and 38 ºC.

The reduction in mTORC1 kinase activity was confirmed by three independent experiments (**new Fig 2F, new Fig 3D, newEV4A**). In particular, a reduction in mTORC1 kinase activity was observed with a short-term (30 and 60 min) temperature drop (37 ºC to 32 ºC), which was inhibited by 2-APB, a TRPVs agonist. Therefore, we concluded that temperature regulates mTORC1 activity through thermo-sensitive TRP channels. We added the following sentence to emphasize the importance of temperature in mTORC1 kinase activity on page 10, lines 16-20.

*"The finding that treatment with 2-APB increased mTORC1 kinase activity even at 32 °C indicates that lower temperatures do not reduce the activity of chemical reactions but regulate mTORC1 activity through thermoTRP channels. Therefore, these experiments support our hypothesis that thermoTRP channels are connected to the mTOR signaling pathway through calcium influx."*

***The RNAseq in Figure 3 is interesting, showing a disruption to the normal differentiation program at low temperatures or with Rapamycin treatment. The analysis of this data is superficial, for example were there any transcriptional hallmarks of Mtor signaling or other pathways? It would be helpful to validate the differentiation phenotype at the protein level. It would be worth discussing literature such as PMID: 27194247 that report that rapalogs block differentiation.***

Based on your suggestion, we performed further gene ontology (GO) analyses and confirmed that rapamycin treatment and low temperature both downregulated the same genes related to many types of metabolic processes and protein translation (**new Fig EV5D**), which are known to be regulated by mTORC1 signaling. GO analysis also confirmed that genes associated with "epidermal development" and "cornification" were downregulated by rapamycin treatment and culture at 32 ºC (**new Fig EV5D**). We have also discussed that our results are consistent with a previous report (DeTemple et al, Exp Dermal 2016), as you suggested.

As you mentioned, impaired differentiation at low temperatures was not observed at the protein level, but cultures at low temperatures significantly reduced cell size (**new Fig 1D**), which is closely associated with the differentiation and proliferative potential of human keratinocytes (Watt and Green, J Cell Biol 1981; Barrandon and Green, Proc Natl Acad Sci USA 1985). These results strongly suggest that low temperatures inhibit the differentiation of human keratinocytes. We have added the following sentence to emphasize the importance of the decreased size of human keratinocytes on page 8, lines 18-21.

*"Cell size is closely associated with the differentiation and proliferative potential of human keratinocytes (Barrandon & Green, 1985; Watt & Green, 1981), which strongly suggests that temperature impacts human keratinocyte stem cell maintenance."*

***Figure 4 shows MTOR translocates to the nucleus, at 36C and with rapamycin treatment. The data look robust but its functional significance is unclear, so this work seems to add little to the paper.***

Thank you for your comments. We could not reveal the functional significance of nuclear mTOR in keratinocytes, but this finding strongly suggests that mTOR complexes directly regulate transcription, as discussed recently (Giguére, FEBS J, 2018; Torres and Holz, BBA Mol Cell Res 2021).

To emphasize the importance of the nuclear localization of mTOR in response to temperature shift and rapamycin treatment, we have added the following sentence to explain why we analyzed the localization of mTOR and associated molecules on page 11, lines 23-26. We also described the importance of the nuclear localization of mTOR and its nuclear localization in keratinocytes before presenting the RNA-seq results to improve the flow of the manuscript.

*"Recent studies have revealed that mTOR complexes translocate to the nucleus and directly regulate transcription (Giguère, 2018; Torres & Holz, 2021). Next, we examined the effects of temperature and rapamycin on the translocation of the mTOR complexes."*

***Figure 5 describes the effects of Rapamycin on long term culture. This is a missed opportunity, as it would be interesting to know what long term culture at 32 or 35C does to cell behavior.***

Thank you for your comment. It takes a long time to prepare epidermal sheets at low temperatures and transplant them into immunodeficient mice. Based on your suggestion, we performed short-term serial cultures of human keratinocytes at 35 ºC and confirmed that colony-forming efficiency is maintained at this temperature even in later passages, similarly to rapamycin treatment, as shown below. [Figures for referees not shown.]

However, the aim of this study was to identify the mechanisms involved in stem cell maintenance and to define culture conditions to maintain stemness and improve stem cell-based regenerative medicine. To apply our findings to regenerative medicine, the application of rapamycin to stem cell cultures is easier than the development of large-scale cell culture devices with precise temperature control, since rapamycin has been used in clinics worldwide for a long time as an immunosuppressive drug.
To emphasize the importance of rapamycin treatment in human keratinocyte cultures and regenerative medicine, we added the following sentence to explain why we intensively analyzed the effects of rapamycin on human keratinocyte stem cell maintenance *ex vivo* on page 14, lines 29-34, and page 15, line 1.

*"In this study, we found that lower temperatures and mTORC1 inhibition maintained human keratinocyte stem cells ex vivo. To apply our findings to regenerative medicine, we further assessed the effects of decreased mTORC1 signaling with rapamycin on human keratinocyte stem cell behavior, as rapamycin is already used in clinics worldwide as an immunosuppressive drug (MacDonald, 2001), and the conventional cell culture systems present a difficulty for precise temperature control."*

***The discussion is quite long and could be better focused.***

According to your suggestion, we removed cornea and aging section from the discussion and focused thermoTRPVs-mTOR signaling axis on human epidermal keratinocyte stem cell maintenance.

**We thank you again for the positive comments and constructive feedback.**

**Point-by-point responses to the editor**

***During a standard image analysis we detected potential aberrations in the figure set, and we would like to clarify these issues before sending your paper back to referees. We kindly invite you to check the composition of westernblots in Figure 2F and Figure 7B yourself, and to send us the related source data. If you make changes to the figure, please include a point-by-point describing what you have changed and why.***

**We appreciate your valuable comments and suggestions. We have carefully checked the Western blots in Figure 2F and 7B, and found some mistakes to present data. According to suggestions, we changed Figure 2F and 7B, and uploaded the source data.**

1. Figure 2F
In this figure, we found the error in the blot presenting S6K1. We checked the original data, and replaced the S6K1 blot with the new one. All source data for Fig 2F were uploaded.

2. Figure 7B
In this figure, we found the errors in the blot presenting TG1, LEKTI, and GAPDH. These errors occurred by the elimination of lane 2 during preparing the figure (Please see the source data). As the experimental conditions in lane 2 and lane 3 were the almost same, we prepared this figure using only lane 1 and lane 2 without eliminating any lanes, and replaced the blots presenting LEKTI, TG1, and GAPDH with the newly prepared ones. All source data for Fig 7B were uploaded.

Dear Dr. Nanba,

Thank you for the submission of your revised manuscript to our editorial offices. I have already forwarded the reports from the three referees that I asked to re-evaluate your study to you, you will find also again below. Referees #2 and #3 now fully support the publication of your study in EMBO reports, although referee #3 has some suggestions to further improve the manuscript.

In contrast, referee #1 states that the study is still un-suited to be published and indicates that several major claims are not supported by the data also after revision. Going through your preliminary p-b-p-response and after further cross-consultation with referee #1, we feel that the remaining concerns will be adequately addressed after a further revision (as indicated in your rebuttal letter). I would thus invite you to address the remining concerns and suggestions of referees #1 and #3 in a further revised manuscript. Please also provide a final p-b-p-response addressing these remaining points.

Moreover, I have these editorial requests I also ask you to address:

- Please move the keywords below the abstract and reduce them to 5.

- We updated our journal's competing interests policy in January 2022 and request authors to consider both actual and perceived competing interests. Please review the policy https://www.embopress.org/competing-interests and update your competing interests if necessary. Please name this section 'Disclosure and Competing Interests Statement' and put it after the Acknowledgements section.

- Please order the manuscript sections like this, using these names:
Title page - Abstract - Keywords - Introduction - Results - Discussion - Materials and Methods - Data availability section - Acknowledgements - Disclosure and Competing Interests Statement - References - Figure legends - Expanded View Figure legends

- Regarding data quantification and statistics, please make sure that the number "n" for how many independent experiments were performed, their nature (biological versus technical replicates), the bars and error bars (e.g. SEM, SD) and the test used to calculate p-values is indicated in the respective figure legends. Please also check that all the p-values are explained in the legend, and that these fit to those shown in the figure. Please provide statistical testing where applicable. Please avoid the phrase 'independent experiment', but clearly state if these were biological or technical replicates. Please also indicate (e.g. with n.s.) if testing was performed, but the differences are not significant.

In case n=2, please show the data as separate datapoints without error bars and statistics.

See also:
http://www.embopress.org/page/journal/14693178/authorguide#statisticalanalysis

If n<5, please show single datapoints for diagrams.

- I would suggest moving the three tables into an Appendix. Please upload these as a single pdf file labeled Appendix. The Appendix should have page numbers and needs to include a table of content on the first page (with page numbers) and legends for all content. Please use the nomenclature Appendix Table Sx and also label the tables according to this nomenclature. Finally, please update all callouts for these tables in the manuscript text file using this nomenclature.

- Please remove the referee tokens for the GEO deposited datasets from the manuscript text. Please add direct links to these datasets to the data availability section and make sure that these datasets are public latest upon online publication of the manuscript.

- Thanks you for providing the source data for some of the Western blots (WBs). As also the remaining Western blots shown are significantly cropped, could you please provide the source data for all the blots. The source data will be published in a separate source data file online along with the accepted manuscript and will be linked to the relevant figure. Please submit the source data (scans of entire blots) together with the final revised manuscript. Please include size markers for the scans of entire blots, label the scans with figure and panel number, and send one PDF file per figure.

- Finally, please find attached a word file of the manuscript text (provided by our publisher) with changes we ask you to include in your final manuscript text and comments. Please use the attached file as basis for further revisions and provide your final manuscript file with track changes, in order that we can see any modifications done.

In addition, I would need from you:
- a short, two-sentence summary of the manuscript (not more than 35 words).
- two to four short bullet points highlighting the key findings of your study (two lines each).

- a schematic summary figure that provides a sketch of the major findings (not a data image) in jpeg or tiff format (with the exact width of 550 pixels and a height of not more than 400 pixels) that can be used as a visual synopsis on our website.

I look forward to seeing the final revised version of your manuscript when it is ready. Please let me know if you have questions regarding the revision.

Please use this link to submit your revision: https://embor.msubmit.net/cgi-bin/main.plex

Best,

Achim Breiling
Senior Editor
EMBO Reports

------------
Referee #1:

The authors have attempted to address my comments, however their results have been largely negative, confirming the concern that the data is shallow and not supporting the story line. The results from revision confirmed to me that this study is unsuitable for publication in a high quality journal like EMBO.

The authors claim that TRPV3 and other thermoTRP channels, are 'activated' at 37C, thereby contributing to a temperature response, but they only show expression data at 37C (figure 2 A,B). These data became even more critical as they found that <<mTORC1 kinase activity is high at 32 {degree sign}C and 37C thus indicating that lower temperatures do not reduce the activity of chemical reactions but regulate mTORC1 activity through thermoTRP channels. Therefore, they claim these experiments support the hypothesis that thermoTRP channels are connected to the mTOR signaling pathway through calcium influx>>. This would require documentation that these channels are temperature regulated, which is missing in this study.

Second, initially the authors were suggesting that TRPV3 channel may be the specific one important for the temperature-dependent regulation of mTORC1 signaling, thus demonstrating this supposed mechanism through these Ca channels. Upon rigorous siRNA testing requested for the revision, this proved to be wrong, leaving them without a specific mechanism by which the regulation occurs and no evidence that the channels are involved at all. Nevertheless, the authors continue to propose that the channels are involved and important.

Third, they were initially suggesting that mTOR goes to the nucleus to regulate target genes, and that then there are gene expression changes in cultured cells. However, they look 10 days after the perturbations, when cell cultures have changed so much in their composition of proliferative and differentiated cells that the RNA-seq data really probes the composition of differentiated or more proliferative cells in cultures and not the actual changes in gene expression that occur from the perturbation. I asked for revisions to verify changes in gene expressions at short time points such as 24 hrs, prior to major changes in cell types in the cultures. They authors did that in the revision, but as feared by me, in fact they could not see any expression changes. This demonstrates that their original hypothesis that mTOR is going to the nucleus and regulating target genes was in fact wrong. Nevertheless, the authors seem to ignore this result as well, and continue with their claim that mTOR regulates gene expression in temperature shift, for which there is absolutely no evidence, and all figure 5 data is meaningless in that respect.

Same goes for data Figure 6, which has the same caveat above, except now they look at miRNAs and not at gene expression.

Lastly, mTOR was already shown to affect keratinocytes cultures (Javier 1997) but the authors claim that here they now demonstrate this works on stem cells, based on data in Figure 7, because they look what they say is 'long-term'. In our world long-term is 6 months to 1 year and not 80 days. And even if we accept the claim that long-term maintenance is modulated by their manipulations, there is no mechanisms involved since data from figure 1-5 is not speciifc to stem cells, and moreover is not meaningful as discussed above.

In conclusion, I am sorry to say that although there has been some work and effort here the quality of the experimental design makes it difficult to interpret the data, and as such I cannot recommend for publication of this study.

------------
Referee #2:

The authors adequately addressed our comments. The manuscript is suitable for publication.

------------
Referee #3:

The authors have substantially improved the manuscript in response to reviewer comments. The text is much clearer and key criticisms have been addressed.

There are some minor points which could be improved. Are the differences in Fig 3C statistically significant? Are the statistical tests 2 tailed? Fig 3 E legend should be D.

The paper includes correlative observations (Figure 4), but overall the findings linking temperature, MTOR signalling and stem cell fate in the physiologically relevant context of skin biology will be of broad interest.

**Point-by-point responses to the reviewers**

**<u>Referee #1:</u>**

***The authors claim that TRPV3 and other thermoTRP channels, are 'activated' at 37C, thereby contributing to a temperature response, but they only show expression data at 37C (figure 2 A,B). These data became even more critical as they found that <<mTORC1 kinase activity is high at 32C and 37C thus indicating that lower temperatures do not reduce the activity of chemical reactions but regulate mTORC1 activity through thermoTRP channels. Therefore, they claim these experiments support the hypothesis that thermoTRP channels are connected to the mTOR signaling pathway through calcium influx>>. This would require documentation that these channels are temperature regulated, which is missing in this study.***

**We appreciate your crucial comments and suggestions on our paper. According to your comments, we have revised the manuscript.**

As you mentioned, we examined the expression of TRPV3 and other thermoTRP channels at $37^{o}$C. However, this study does not need to consider the expression changes of thermoTRP channels since mTORC1 kinase activity is modified by short-term temperature shifts (30-60 min) in Fig 2F. In Fig 2G, keratinocytes are also treated with menthol and 2-APB for 60 min. Moreover, the research in temperature biology also does not consider the expression changes of thermoTRP channels when cultured keratinocytes are treated with menthol and 2-ABP (Chung et al J Neurosci 2004; Bidaux et al, Proc Natl Acad Sci USA 2015). However, we did not clearly describe the duration of menthol and 2-APB treatment before cell lysis, which makes readers confused. Therefore, we added the following sentences into the Figure legend 2 (page 38).

***(D and E)*** *"RuR and menthol were added into the keratinocyte cultures 1 h before starting the temperature changes."*
***(F and G)*** *"Rapamycin (Rapa), menthol, and 2-APB were added into the keratinocyte cultures 1 h before starting the temperature changes."*

TRP channels are mostly permeable to calcium, so their functionality can be assessed using a fluorescent calcium indicator. In Fig 2D, the addition of RuR, a broad thermoTRP channel antagonist, prevented calcium influx at 37$^{o}$C, which indicates that several types of thermoTRP channels were activated at 37$^{o}$C. In Fig 2E, menthol, a TRPM8 agonist, induced calcium influx at 37$^{o}$C, which indicates that TRPM8 was not activated at 37$^{o}$C. Thus, we demonstrated the temperature-regulated functionality of TRP channels in human keratinocytes. Furthermore, in Fig 2F and 2G, the activities of thermoTRP channels were linked to mTORC1 kinase activity.

The notion that temperature regulates the activity of thermoTRP channels in epidermal keratinocytes is well-established as reviewed in Lumpkin & Caterina 2007, Venkatachalam & Montell 2007, and Blaydon & Kelsell 2014, and we cited these review articles in the "Introduction" section (page 4) as follows. Collectively, we concluded that temperature regulates the mTORC1 kinase activity by modulating the activity of thermoTRP channels in human keratinocytes.

*"There is accumulating evidence that keratinocytes sense their environment through the activation of ion channels with sensory properties (Blaydon & Kelsell, 2014; Lumpkin & Caterina, 2007; Venkatachalam & Montell, 2007), most of which belong to the transient receptor potential (TRP) ion channel superfamily."*

***Second, initially the authors were suggesting that TRPV3 channel may be the specific one important for the temperature-dependent regulation of mTORC1 signaling, thus demonstrating this supposed mechanism through these Ca channels. Upon rigorous siRNA testing requested for the revision, this proved to be wrong, leaving them without a specific mechanism by which the regulation occurs and no evidence that the channels are involved at all. Nevertheless, the authors continue to propose that the channels are involved and important.***

As you mentioned, we could not demonstrate that the activity of TRPV3 is directly involved in mTORC1 signaling (Fig EV3). However, this result does not exclude the possibility that other thermoTRP channels are involved in thermo-sensitive regulation of mTORC1 kinase activity. In Fig 2G, we demonstrated that mTORC1 kinase activity is regulated by menthol, an agonist of TRPM8, and 2-APB, an agonist of TRPV1, 2, and 3. We mentioned the connection between thermoTRP channels and mTORC1 signaling in

the manuscript, but we did not describe the TRPV3-mTORC1 axis in the manuscript. We have described it as follows in the manuscript (page 10).

*"This result strongly suggests that TRPV3 activity does not mainly contribute to the temperature-dependent regulation of mTORC1 signaling and that the temperature drop cooperatively sensed through several types of thermoTRP channels inhibits mTORC1 signaling similar to rapamycin treatment."*

***Third, they were initially suggesting that mTOR goes to the nucleus to regulate target genes, and that then there are gene expression changes in cultured cells. However, they look 10 days after the perturbations, when cell cultures have changed so much in their composition of proliferative and differentiated cells that the RNA-seq data really probes the composition of differentiated or more proliferative cells in cultures and not the actual changes in gene expression that occur from the perturbation. I asked for revisions to verify changes in gene expressions at short time points such as 24 hrs, prior to major changes in cell types in the cultures. They authors did that in the revision, but as feared by me, in fact they could not see any expression changes. This demonstrates that their original hypothesis that mTOR is going to the nucleus and regulating target genes was in fact wrong. Nevertheless, the authors seem to ignore this result as well, and continue with their claim that mTOR regulates gene expression in temperature shift, for which there is absolutely no evidence, and all figure 5 data is meaningless in that respect.***

***Same goes for data Figure 6, which has the same caveat above, except now they look at miRNAs and not at gene expression.***

Fig 5 and 6 show the expression changes of mRNAs and miRNAs in response to temperature shifts. As you pointed out, the 10 days of cultivation changes the composition of proliferative and differentiated cells. However, our data clearly revealed that lower temperatures inhibit the spontaneous differentiation of human keratinocytes in the culture, which has not been demonstrated so far. We believe that this information is not meaningless in the related research communities.

As you mentioned, we could not demonstrate the expression changes of mRNAs associated with epidermal differentiation in a short-term period (24 h). However, the

nuclear translocation of mTOR at the lower temperature and rapamycin treatment in human keratinocytes suggests that some genes are directly regulated by nuclear mTOR as described (Giguére, FEBS J; Torres and Holz, BBA Mol Cell Res 2021), and the expressions of mRNAs associated with epidermal differentiation might be secondary modified by genes that are directly regulated by nuclear mTOR. In the manuscript, we did not describe that the nuclear translocation of mTOR directly regulates the expression of mRNAs associated with epidermal differentiation. We simply mentioned that lower temperature and rapamycin treatment impact gene expression in the manuscript. The nuclear translocation of mTOR in cultured human keratinocytes at 35°C and rapamycin treatment is a very interesting phenomenon and we would like to share this information among research communities by including Fig 4 in this paper as a correlative observation, which will advance this new research field.

However, we did not clearly describe whether nuclear mTOR regulates keratinocyte behaviors, which makes readers confused as you pointed out. Therefore, we added the following sentences to the "Discussion" section (page 17).

*"However, it remains unknown that nuclear mTOR is involved in regulating keratinocyte behaviors, and further investigations should be required."*

***Lastly, mTOR was already shown to affect keratinocytes cultures (Javier 1997) but the authors claim that here they now demonstrate this works on stem cells, based on data in Figure 7, because they look what they say is 'long-term'. In our world long-term is 6 months to 1 year and not 80 days. And even if we accept the claim that long-term maintenance is modulated by their manipulations, there is no mechanisms involved since data from figure 1-5 is not specific to stem cells, and moreover is not meaningful as discussed above.***

We could maintain human keratinocyte stem cells in the culture with rapamycin even if the cultures are passaged more than 10 times (more than 70 days' cultivation) in Fig 7C. Rapamycin-treated keratinocytes were also maintained after 20 passages (Fig 7D). Human keratinocyte stem cells were completely exhausted at passage 20 under normal culture conditions, which is described in Figure legend 7D as follows (page 41).

*"Telomere length was analyzed on DNA extracted from YF29 keratinocytes with or*

*without rapamycin treatment at passages VIII, XII and XX (keratinocytes grown without rapamycin did not reach passage XX because of clonal conversion - see panel C).”*

These data clearly demonstrate that mTORC1 inhibition with rapamycin favors the maintenance of keratinocyte stem cells since the number of keratinocyte stem cells is significantly decreased during serial cultivation (Fig 7C). In addition, we have not used the phrase “long-term maintenance of keratinocyte stem cells” in the text, except for “long-term growth of keratinocyte stem cells” in Figure legend 1. We have used the phrase “long-term” as “long-term inhibition of mTORC1”, “long-term rapamycin treatment”, and “long-term exposure of keratinocyte stem cells to rapamycin”. We have mainly used the phrase “long-term” to explain the experimental condition in the manuscript.

As you pointed out, we used bulk keratinocyte cultures including the stem cells for the experiments in Fig 1-6, but colony-formation assay in Fig 1 and 3 clearly demonstrate that temperature shifts and rapamycin treatment impact the clonal growth of keratinocytes with significant proliferative capacity. Our experiments indicate that lower temperatures and rapamycin treatment inhibit spontaneous differentiation in human keratinocytes including stem cells and limited-proliferative cells as shown in Fig 5 and 7. This effect might be not specific to stem cells, but becomes more evident in stem cells. Furthermore, lower temperatures and rapamycin treatment decreased the keratinocyte size as shown in Fig 1D and 3C. These findings also indicate that the inhibition of mTORC1 signaling by a cooler environment or rapamycin treatment favors stem cell maintenance in human keratinocyte cultures since small keratinocytes are highly clonogenic (Barrandon & Green, Proc Natl Acad Sci USA 1985). Our study provides a new insight into the expansion and maintenance of cultured human keratinocyte stem cells through thermo-sensitive regulation of mTORC1 activity.

**Thank you again for your criticism which makes us realize the limitations and weaknesses of our study. We will start further studies to answer your questions.**

**<u>Referee #2:</u>**

***The authors adequately addressed our comments. The manuscript is suitable for publication.***

**We appreciate your positive comments and suggestions for publication.**

**Referee #3:**

***The authors have substantially improved the manuscript in response to reviewer comments. The text is much clearer and key criticisms have been addressed.***
***There are some minor points which could be improved. Are the differences in Fig 3C statistically significant? Are the statistical tests 2 tailed? Fig 3 E legends should be D.***
***The paper includes correlative observations (Figure 4), but overall the findings linking temperature, MTOR signalling and stem cell fate in the physiologically relevant context of skin biology will be of broad interest.***

**We appreciate your positive comments and suggestions for publication. According to your comments, we have revised the manuscript.**

According to your suggestions, we have checked the all statistical tests and described the statistical significance, the types of statistical tests, and the number of samples, including Figure 3. We have also corrected the Figure legend number (Fig 3E to 3F), as you pointed out.

**Thank you again for your comments for improving the manuscript.**

Dr. Daisuke Nanba
The University of Tokyo
The Institute of Medical Science
4-6-1 Shirokanedai
Minato-ku
Tokyo
Japan

Dear Dr. Nanba,

Thank you for the submission of your further revised manuscript. I now went through this again and your final p-b-p-response and consider the remaining referee points as adequately addressed.

Thus, I am very pleased to accept your manuscript for publication in the next available issue of EMBO reports. Thank you for your contribution to our journal.

At the end of this email I include important information about how to proceed. Please ensure that you take the time to read the information and complete and return the necessary forms to allow us to publish your manuscript as quickly as possible.

As part of the EMBO publication's Transparent Editorial Process, EMBO reports publishes online a Review Process File to accompany accepted manuscripts. As you are aware, this File will be published in conjunction with your paper and will include the referee reports, your point-by-point response and all pertinent correspondence relating to the manuscript.

If you do NOT want this File to be published, please inform the editorial office within 2 days, if you have not done so already, otherwise the File will be published by default [contact: emboreports@embo.org]. If you do opt out, the Review Process File link will point to the following statement: "No Review Process File is available with this article, as the authors have chosen not to make the review process public in this case."

Thank you again for your contribution to EMBO reports and congratulations on a successful publication. Please consider us again in the future for your most exciting work.

Yours sincerely,

Achim Breiling
Editor
EMBO Reports

**********************************************************************************

THINGS TO DO NOW:

Please note that you will be contacted by Wiley Author Services to complete licensing and payment information. The required 'Page Charges Authorization Form' is available here: https://www.embopress.org/pb-assets/embo-site/er_apc.pdf - please download and complete the form and return to embopressproduction@wiley.com

EMBO Press participates in many Publish and Read agreements that allow authors to publish Open Access with reduced/no publication charges. Check your eligibility: https://authorservices.wiley.com/author-resources/Journal-Authors/open-access/affiliation-policies-payments/index.html

You will receive proofs by e-mail approximately 2-3 weeks after all relevant files have been sent to our Production Office; you should return your corrections within 2 days of receiving the proofs.

Please inform us if there is likely to be any difficulty in reaching you at the above address at that time. Failure to meet our deadlines may result in a delay of publication, or publication without your corrections.

All further communications concerning your paper should quote reference number EMBOR-2022-55439V3 and be addressed to emboreports@wiley.com.

Should you be planning a Press Release on your article, please get in contact with emboreports@wiley.com as early as possible, in order to coordinate publication and release dates.

## EMBO Press Author Checklist

| | |
|---|---|
| Corresponding Author Name: Daisuke Nanba | |
| Journal Submitted to: EMBO reports | |
| Manuscript Number: EMBOR-2022-55439V2 | |

**USEFUL LINKS FOR COMPLETING THIS FORM**
The EMBO Journal - Author Guidelines
EMBO Reports - Author Guidelines
Molecular Systems Biology - Author Guidelines
EMBO Molecular Medicine - Author Guidelines

**Reporting Checklist for Life Science Articles (updated January 2022)**
This checklist is adapted from Materials Design Analysis Reporting (MDAR) Checklist for Authors. MDAR establishes a minimum set of requirements in transparent reporting in the life sciences (see Statement of Task: 10.31222/osf.io/9sm4x). Please follow the journal's guidelines in preparing your manuscript.
**Please note that a copy of this checklist will be published alongside your article.**

**Abridged guidelines for figures**
**1. Data**
The data shown in figures should satisfy the following conditions:
- the data were obtained and processed according to the field's best practice and are presented to reflect the results of the experiments in an accurate and unbiased manner.
- ideally, figure panels should include only measurements that are directly comparable to each other and obtained with the same assay.
- plots include clearly labeled error bars for independent experiments and sample sizes. Unless justified, error bars should not be shown for technical replicates.
- if n<5, the individual data points from each experiment should be plotted. Any statistical test employed should be justified.
- Source Data should be included to report the data underlying figures according to the guidelines set out in the authorship guidelines on Data Presentation.

**2. Captions**
Each figure caption should contain the following information, for each panel where they are relevant:
- a specification of the experimental system investigated (eg cell line, species name).
- the assay(s) and method(s) used to carry out the reported observations and measurements.
- an explicit mention of the biological and chemical entity(ies) that are being measured.
- an explicit mention of the biological and chemical entity(ies) that are altered/varied/perturbed in a controlled manner.
- the exact sample size (n) for each experimental group/condition, given as a number, not a range;
- a description of the sample collection allowing the reader to understand whether the samples represent technical or biological replicates (including how many animals, litters, cultures, etc.).
- a statement of how many times the experiment shown was independently replicated in the laboratory.
- definitions of statistical methods and measures:
  - common tests, such as t-test (please specify whether paired vs. unpaired), simple χ2 tests, Wilcoxon and Mann-Whitney tests, can be unambiguously identified by name only, but more complex techniques should be described in the methods section;
  - are tests one-sided or two-sided?
  - are there adjustments for multiple comparisons?
  - exact statistical test results, e.g., P values = x but not P values < x;
  - definition of 'center values' as median or average;
  - definition of error bars as s.d. or s.e.m.

**Please complete ALL of the questions below.**
**Select "Not Applicable" only when the requested information is not relevant for your study.**

**Materials**

| Newly Created Materials | Information included in the manuscript? | In which section is the information available? (Reagents and Tools Table, Materials and Methods, Figures, Data Availability Section) |
|---|---|---|
| New materials and reagents need to be available; do any restrictions apply? | Not Applicable | |

| Antibodies | Information included in the manuscript? | In which section is the information available? (Reagents and Tools Table, Materials and Methods, Figures, Data Availability Section) |
|---|---|---|
| For **antibodies** provide the following information:<br>- Commercial antibodies: RRID (if possible) or supplier name, catalogue number and or/clone number<br>- Non-commercial: RRID or citation | Yes | Table |

| DNA and RNA sequences | Information included in the manuscript? | In which section is the information available? (Reagents and Tools Table, Materials and Methods, Figures, Data Availability Section) |
|---|---|---|
| **Short novel DNA or RNA including primers, probes**: provide the sequences. | Yes | Table |

| Cell materials | Information included in the manuscript? | In which section is the information available? (Reagents and Tools Table, Materials and Methods, Figures, Data Availability Section) |
|---|---|---|
| **Cell lines:** Provide species information, strain. Provide accession number in repository **OR** supplier name, catalog number, clone number, and/**OR** RRID. | Not Applicable | |
| **Primary cultures:** Provide species, strain, sex of origin, genetic modification status. | Yes | Materials and Methods |
| Report if the cell lines were recently **authenticated** (e.g., by STR profiling) and tested for mycoplasma contamination. | Not Applicable | |

| Experimental animals | Information included in the manuscript? | In which section is the information available? (Reagents and Tools Table, Materials and Methods, Figures, Data Availability Section) |
|---|---|---|
| **Laboratory animals or Model organisms:** Provide species, strain, sex, age, genetic modification status. Provide accession number in repository **OR** supplier name, catalog number, clone number, **OR** RRID. | Yes | Materials and Methods |
| **Animal observed in or captured from the field:** Provide species, sex, and age where possible. | Not Applicable | |
| Please detail **housing and husbandry conditions**. | Yes | Materials and Methods |

| Plants and microbes | Information included in the manuscript? | In which section is the information available? (Reagents and Tools Table, Materials and Methods, Figures, Data Availability Section) |
|---|---|---|
| **Plants:** provide species and strain, ecotype and cultivar where relevant, unique accession number if available, and source (including location for collected wild specimens). | Not Applicable | |
| **Microbes:** provide species and strain, unique accession number if available, and source. | Not Applicable | |

| Human research participants | Information included in the manuscript? | In which section is the information available? (Reagents and Tools Table, Materials and Methods, Figures, Data Availability Section) |
|---|---|---|
| If collected and within the bounds of privacy constraints report on age, sex and gender or ethnicity for all study participants. | Not Applicable | |

| Core facilities | Information included in the manuscript? | In which section is the information available? (Reagents and Tools Table, Materials and Methods, Figures, Data Availability Section) |
|---|---|---|
| If your work benefited from core facilities, was their service mentioned in the acknowledgments section? | Yes | Acknowlegements |

**Design**

| Study protocol | Information included in the manuscript? | In which section is the information available? (Reagents and Tools Table, Materials and Methods, Figures, Data Availability Section) |
|---|---|---|
| If study protocol has been **pre-registered, provide DOI in the manuscript**. For clinical trials, provide the trial registration number **OR** cite DOI. | Not Applicable | |
| Report the **clinical trial registration number** (at ClinicalTrials.gov or equivalent), where applicable. | Not Applicable | |

| Laboratory protocol | Information included in the manuscript? | In which section is the information available? (Reagents and Tools Table, Materials and Methods, Figures, Data Availability Section) |
|---|---|---|
| Provide DOI OR other citation details if **external detailed step-by-step protocols** are available. | Not Applicable | |

| Experimental study design and statistics | Information included in the manuscript? | In which section is the information available? (Reagents and Tools Table, Materials and Methods, Figures, Data Availability Section) |
|---|---|---|
| Include a statement about **sample size** estimate even if no statistical methods were used. | Yes | Figure legends |
| Were any steps taken to minimize the effects of subjective bias when allocating animals/samples to treatment (e.g. **randomization procedure**)? If yes, have they been described? | Not Applicable | |
| Include a statement about **blinding** even if no blinding was done. | Not Applicable | |
| Describe **inclusion/exclusion criteria** if samples or animals were excluded from the analysis. Were the criteria pre-established? If sample or data points were omitted from analysis, report if this was due to attrition or intentional exclusion and provide justification. | Not Applicable | |
| For every figure, are **statistical tests** justified as appropriate? Do the data meet the assumptions of the tests (e.g., normal distribution)? Describe any methods used to assess it. Is there an estimate of variation within each group of data? Is the variance similar between the groups that are being statistically compared? | Yes | Figure legends |

| Sample definition and in-laboratory replication | Information included in the manuscript? | In which section is the information available? (Reagents and Tools Table, Materials and Methods, Figures, Data Availability Section) |
|---|---|---|
| In the figure legends: state number of times the experiment was **replicated** in laboratory. | Yes | Figure legends |
| In the figure legends: define whether data describe **technical or biological replicates**. | Yes | Figure legends |

## Ethics

| Ethics | Information included in the manuscript? | In which section is the information available? (Reagents and Tools Table, Materials and Methods, Figures, Data Availability Section) |
|---|---|---|
| Studies involving **human participants**: State details of **authority granting ethics approval** (IRB or equivalent committee(s), provide reference number for approval. | Not Applicable | |
| Studies involving **human participants**: Include a statement confirming that **informed consent** was obtained from all subjects and that the experiments conformed to the principles set out in the WMA Declaration of Helsinki and the Department of Health and Human Services Belmont Report. | Not Applicable | |
| Studies involving **human participants:** For publication of **patient photos**, include a statement confirming that consent to publish was obtained. | Not Applicable | |
| Studies involving experimental **animals**: State details of **authority granting ethics approval** (IRB or equivalent committee(s), provide reference number for approval. Include a statement of compliance with ethical regulations. | Yes | Materials and Methods |
| Studies involving **specimen and field samples:** State if relevant **permits** obtained, provide details of authority approving study; if none were required, explain why. | Not Applicable | |

| Dual Use Research of Concern (DURC) | Information included in the manuscript? | In which section is the information available? (Reagents and Tools Table, Materials and Methods, Figures, Data Availability Section) |
|---|---|---|
| Could your study fall under dual use research restrictions? Please check biosecurity documents and list of **select agents and toxins** (CDC): https://www.selectagents.gov/sat/list.htm | Not Applicable | |
| If you used a select agent, is the security level of the lab appropriate and reported in the manuscript? | Not Applicable | |
| If a study is subject to dual use research of concern regulations, is the name of the **authority granting approval and reference number** for the regulatory approval provided in the manuscript? | Not Applicable | |

## Reporting

The MDAR framework recommends adoption of discipline-specific guidelines, established and endorsed through community initiatives. Journals have their own policy about requiring specific guidelines and recommendations to complement MDAR.

| Adherence to community standards | Information included in the manuscript? | In which section is the information available? (Reagents and Tools Table, Materials and Methods, Figures, Data Availability Section) |
|---|---|---|
| State if relevant guidelines or checklists (e.g., **ICMJE, MIBBI, ARRIVE, PRISMA**) have been followed or provided. | Not Applicable | |
| For **tumor marker prognostic studies,** we recommend that you follow the **REMARK** reporting guidelines (see link list at top right). See author guidelines, under 'Reporting Guidelines'. Please confirm you have followed these guidelines. | Not Applicable | |
| For **phase II and III randomized controlled trials**, please refer to the **CONSORT** flow diagram (see link list at top right) and submit the CONSORT checklist (see link list at top right) with your submission. See author guidelines, under 'Reporting Guidelines'. Please confirm you have submitted this list. | Not Applicable | |

## Data Availability

| Data availability | Information included in the manuscript? | In which section is the information available? (Reagents and Tools Table, Materials and Methods, Figures, Data Availability Section) |
|---|---|---|
| Have **primary datasets** been deposited according to the journal's guidelines (see 'Data Deposition' section) and the respective accession numbers provided in the Data Availability Section? | Yes | Data Availability |
| Were **human clinical and genomic datasets** deposited in a public access-controlled repository in accordance to ethical obligations to the patients and to the applicable consent agreement? | Not Applicable | |
| Are **computational models** that are central and integral to a study available without restrictions in a machine-readable form? Were the relevant accession numbers or links provided? | Not Applicable | |
| If publicly available data were reused, provide the respective **data citations in the reference list**. | Not Applicable | |
